# Target validation and structure–activity analysis of a series of novel PCNA inhibitors

**DOI:** 10.1002/prp2.115

**Published:** 2015-02-02

**Authors:** Kelsey L Dillehay, William L Seibel, Daoli Zhao, Shan Lu, Zhongyun Dong

**Affiliations:** 1 Department of Internal Medicine, University of Cincinnati College of MedicineCincinnati, OH, 45267; 2 Department of Pediatrics, Experimental Hematology and Cancer Biology, Cincinnati Children's Hospital Medical CenterCincinnati, OH, 46119; 3 Department of Chemistry, University of Cincinnati College of MedicineCincinnati, OH, 45219; 4 Department of Pathology and Laboratory Medicine, University of Cincinnati College of MedicineCincinnati, OH, 45219

**Keywords:** Chromatin association, inhibition of tumor cell growth, PCNA, small molecule inhibitors, target validation

## Abstract

Proliferating cell nuclear antigen (PCNA) plays an essential role in DNA replication and repair. Tumor cells express high levels of PCNA, identifying it as a potentially ideal target for cancer therapy. Previously, we identified nine compounds termed PCNA inhibitors (PCNA-Is) that bind directly to PCNA, stabilize PCNA trimer structure, reduce chromatin-associated PCNA, and selectively inhibit tumor cell growth. Of these compounds, PCNA-I1 was most potent. The purpose of this study is to further establish targeting of PCNA by PCNA-I1 and to identify PCNA-I1 analogs with superior potencies. We found that PCNA-I1 does not affect the level of chromatin-associated PCNA harboring point mutations at the predicted binding site of PCNA-I1. Forty-six PCNA-I1 analogs with structures of 1-hydrazonomethyl-2-hydroxy (scaffold A), 2-hydrazonomethyl-1-hydroxy (scaffold B), 2-hydrazonomethyl-3-hydroxy (scaffold C), and 4-pyridyl hydrazine (scaffold D) were analyzed for their effects on cell growth in four tumor cell lines and PCNA trimer stabilization. Compounds in scaffold group A and group B showed the highest trimer stabilization and the most potent cell growth inhibitory activities with a significant potency advantage observed in the Z isomers of scaffold A. The absence of trimer stabilization and growth inhibitory effects in compounds of scaffold group D confirms the essentiality of the hydroxynaphthyl substructure. Compounds structure–activity relationship (SAR)-6 and SAR-24 were analyzed for their effects on and found to reduce chromatin-associated PCNA in tumor cells. This study led to the identification of SAR-24, a compound with superior potencies and potentially improved solubility, which will be used for future development of PCNA-targeting cancer therapies.

## Introduction

Proliferating cell nuclear antigen (PCNA) is an evolutionally well-conserved nononcogenic protein ubiquitously expressed in all types of cells. Human PCNA is a 30-kDa nuclear protein of 261 amino acid residues (Almendral et al. 1987; Naryzhny et al. 2006) with several domains: an interdomain connecting loop (IDCL) linking the N- and C-terminal domains, a center loop, a back side loop, and a C-terminus (Naryzhny 2008). PCNA is present as ring-shaped homotrimers: two monomers are joined together in an antiparallel tail to head interaction through amide-to-carboxyl hydrogen bonds between two *β* sheets, a small hydrophobic core, and putative ion pairs (Krishna et al. 1994; Kelman and O'Donnell 1995; Gulbis et al. 1996; Naryzhny 2008). The majority of PCNA is nonchromatin associated (the free form). To execute most functions, PCNA trimers must be loaded to DNA by the replication factor C (RFC) complex (Waga and Stillman 1998; Sakato et al. 2012; Hedglin et al. 2013). Extensive interactions between RFCs and PCNA homotrimers open the PCNA ring. The engagement of RFC:PCNA complex with the primer-template junctions of DNA results in ATP hydrolysis, closing of the ring, and release of the PCNA sliding clamp on DNA (Fukuda et al. 1995; Bowman et al. 2004; Sakato et al. 2012; Hedglin et al. 2013). The chromatin-associated PCNA encircles and slides along the double-strand DNA (Kelman 1997). PCNA plays crucial roles in numerous cellular processes, such as DNA replication and repair, cell survival, cell cycle control, and chromatin assembly (Kelman and Hurwitz 1998; Moldovan et al. 2007; Naryzhny 2008; Stoimenov and Helleday 2009). It executes these crucial roles through interaction with over 400 protein partners, including DNA polymerase *δ* and *ε* for DNA replication, DNMT1, HDAC1, and p300 for chromatin assembly and gene regulation, DNA mismatch repair protein Msh3 and Msh6 for DNA repair, p21, p15, cyclin D1, and CDK2 for cell cycle control, and ESCO1 and ESCO2 for sister-chromatid cohesion (Maga and Hubscher 2003; Stoimenov and Helleday 2009). These partner proteins interact with different domains of PCNA through the PIP-box (PCNA-interaction protein box), KA-box, AlkB homologue 2 PCNA-interacting motif (APIM), and other motifs (Gilljam et al. 2009; Stoimenov and Helleday 2009). In addition, several recent studies suggest that PCNA may function in the cytoplasm, potentially involved in apoptosis regulation in neutrophils (Witko-Sarsat et al. 2010), inhibition of natural cytotoxicity factor activity (Rosental et al. 2011), and interaction with glycolytic enzymes (Naryzhny and Lee 2010). The critical importance of PCNA for cell growth and survival is underscored by the finding that a homozygous deletion of PCNA is embryonically lethal in mice (Roa et al. 2008).

Previously, we performed docking/screening of a library with 3 × 10^5^ drug-like compound structures (The University of Cincinnati Drug Discovery Center, UC-DDC) against a model derived from an X-ray crystal structure of human PCNA (Protein Data Bank code: 1VYJ). The top 200 hits that potentially bind to the interfaces between two monomers of a PCNA trimer were selected for further evaluation in bioassays and nine PCNA-Is were identified. These PCNA-Is bind directly to and stabilize PCNA trimer structure in vitro and reduce chromatin-associated PCNA in cells (Tan et al. 2012). PCNA-I1, the most potent among the nine compounds, inhibits PCNA-dependent DNA synthesis in vitro (data not published) and DNA replication in tumor cells (Tan et al. 2012). The inhibitory effects of PCNA-Is on cell cycle distribution can be mimicked by knocking down PCNA expression (Tan et al. 2012). Moreover, PCNA-I1 selectively inhibits growth of tumor cells of various tissue origins (Tan et al. 2012). In efforts to identify more potent and/or more soluble compounds and extend the pharmacophoric observations around PCNA-I1, we performed an initial structure–activity relationship (SAR) analysis. A series of PCNA-I1 analogs were obtained from the UC-DDC compound library or commercial sources and evaluated in assays for PCNA trimer stability in vitro, growth inhibitory effects in four cancer cell lines, and the level of chromatin-associated PCNA. Several novel compounds with potencies superior to PCNA-I1 were identified.

## Materials and Methods

### Reagents

The PCNA-I1 analogs derived from SAR analysis were named as SAR compounds. All SAR compounds, except those specified below, were obtained from the UC-DDC. SAR-11 was purchased from Chembridge Co (San Diego, CA). SAR-15 and SAR-16 were purchased from ChemDiv (San Diego, CA). SAR-17, SAR-34, SAR-35, SAR-36, SAR-37, and SAR-38 were purchased from Vitas-M Laboratory (Moscow, Russia). SAR-19 was purchased from TimTec (Newark, DE). The nuclear magnetic resonance (NMR) and mass spectrometry data of the compounds are provided in Data S1. The recombinant His-PCNA (>95% pure) was purchased from Abcam (Cambridge, MA). Antibody against PCNA (PC10), *α*-tubulin, and Histone 3 were purchased from Cell Signaling Technology (Danvers, MA). Lipofectamine 2000 reagent was purchased from Invitrogen (Carlsbad, CA). Protease inhibitor cocktail, deoxyribonuclease I from bovine pancreas, and 3-(4,5-dimethylthiazol-2-yl)-2,5-diphenyltetrazolium bromide (MTT) were purchased from Sigma-Aldrich (St. Louis, MO). The enhanced chemiluminescence Western Blotting Detection System was purchased from Millipore (Billerica, MA).

### Cells and culture

LNCaP, 22Rv1, and PC-3 human prostate cancer cells, and A549 human lung adenocarcinoma cells were obtained from ATCC (Manassas, VA) and maintained at 37°C in 5% CO_2_. LNCaP, 22Rv1, and A549 cells were cultured in RPMI-1640 medium supplemented with 10% fetal bovine serum (FBS). PC-3 cells were cultured in MEM/Earle's Balanced Salts (EBSS) medium supplemented with 5% FBS. Cells in exponential growth phase were harvested by a 1–3 min treatment with a 0.25% trypsin −0.02% Ethylenediaminetetraacetic acid (EDTA) solution and resuspended in the specified medium. Only suspensions of single cell with viability exceeding 95% (ascertained by trypan blue exclusion) were used.

### PCNA trimer stability assay

PCNA trimer stability was assessed as described in our previous study (Tan et al. 2012). Briefly, 0.1 *μ*g of His-PCNA was incubated for 3 h at room temperature with 10 *μ*mol/L SAR compounds or Dimethyl sulfoxide (DMSO) (0.1%, vehicle) in a reaction buffer (40 mmol/L Tris-HCl, pH7.5, 0.2 mg/mL bovine serum albumin, 10 mmol/L MgCl_2_, and 10% glycerol). The reaction was stopped by addition of 2x Laemmli sample buffer without the reducing agent 2-mercaptoethanol. The samples were resolved by sodiumdodecyl sulphate-polyacrylamide gel electrophoresis (SDS-PAGE) without boiling and analyzed by immunoblotting with PCNA antibody. The immunoreactive signals were revealed using the enhanced chemiluminescence method, visualized using the Kodak IS4000MM Digital Imaging System (Carestream Health, Rochester, NY), and analyzed by densitometry.

### Nuclear fractionation and PCNA chromatin association

Chromatin-associated PCNA was analyzed as described previously (Tan et al. 2012). PC-3 cells were treated with PCNA-I1 or the SAR compounds for 8 h and collected by trypsinization. The cells were pelleted (300 g, 5 min, 4°C), washed in PBS with 1 mmol/L phenylmethylsulfonyl fluoride (PMSF), and lysed in buffer A (10 mmol/L Tris-HCl, pH 7.4, 2.5 mmol/L MgCl_2_, 0.5% Nonidet P-40, 1 mmol/L dithiothreitol, 1 mmol/L PMSF, and protease inhibitor cocktail). The lysate was centrifuged for 2 min at 1500*g* (4°C) and the resulting supernatant fraction collected and designated as the NP-40 extractable (NP-E) fraction. The pellet was washed in buffer B (10 mmol/L Tris-HCl, pH 7.4, 150 mmol/L NaCl, 1 mmol/L PMSF, and protease inhibitor cocktail), resuspended and digested in buffer C (10 mmol/L Tris-HCl, pH 7.4, 10 mmol/L NaCl, 5 mmol/L MgCl_2_, 0.2 mmol/L PMSF, and protease inhibitor cocktail) with 200 units/10^7^ cells of DNase I for 30 min with agitation at room temperature. After centrifugation (13,000 g, 5 min, 4°C) the supernatant was collected and designated as the NP-40-resistant (NP-R) fraction. The samples were then resolved by SDS-PAGE and analyzed by immunoblotting with PCNA, Histone 3, and *α*-tubulin antibodies.

### Cell growth assay

Effects of the compounds on cell growth were assessed by MTT staining as described previously (Actis et al. 2013). Growth inhibition (percentage) by the compounds was calculated using the formula: (1-*A*_570_ of treated/*A*_570_ of control) × 100 and the IC_50_ were determined.

### PCNA mutagenesis

The mammalian expression vector pGFP-PCNA (Leonhardt et al. 2000), encoding the green fluorescent protein (GFP)-tagged human PCNA, was kindly provided by Dr. Shao-Chun Wang (Dept of Cancer and Cell Biology, University of Cincinnati). An in vitro site-directed mutagenesis was used to introduce point mutations into pGFP-PCNA to generate mutant pGFP-PCNAs (pGFP-PCNAmu) with the following primers purchased from Eurofins MWG Operon (Huntsville, AL):

D86N-1:5-GCGCCGGCAATGAAAATATCATTACACTAAGGGC-3′, D86N-2:5′-GCCCTTAGTGTAATGATATTTTCATTGCCGGCGC-3′, K110I-1:5′GCACCAAACCAGGAGATAGTTTCAGACTATGAAATG-3′, K110I-2:5′-CATTTCATAGTCTGAAACTATCTCCTGGTTTGGTGC-3′, R146L-1:5′-CTTCTGGTGAATTTGCACTTATATGCCGAGATCTCAG-3′, R146L-2:5′-CTGAGATCTCGGCATATAAGTGCAAATTCACCAGAAG-3′. Mutant PCNA plasmids were generated using the QuikChange II XL Site-Directed Mutagenesis Kit from Agilent Technologies (Santa Clara, CA) following the manufacturer's protocol.

### Transfection of mutant PCNA

PC-3 cells were plated onto 100-cm plate at 1 × 10^6^/plate in antibiotic-free media and allowed to adhere overnight. The next day PC-3 cells were transfected with 12 *μ*g of wild-type pGFP-PCNA and pGFP-PCNAmu plasmids using Lipofectamine 2000 following the manufacturer's instructions. After 6 h the media was changed and the cells were observed for GFP expression the following day under a fluorescence microscope and used in experiments analyzing chromatin-associated PCNA.

### Statistical analysis

Data shown are the mean ± standard deviation. Correlation between IC_50_ and trimer stability amongst the four cells lines were determined by linear regression analysis using GraphPad Prism version 4.0 (San Diego, CA).

## Results

### Effects of PCNA-I1 on mutant PCNA association with chromatin

**Figure 1:**
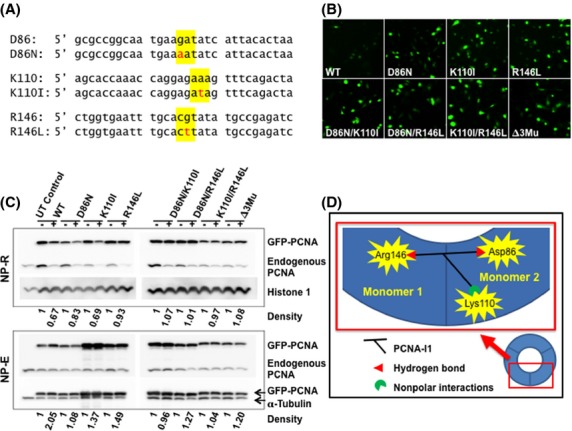
Effects of PCNA-I1 on mutant PCNA association with chromatin. (A) Point mutations introduced into the wild-type pGFP-PCNA expression vector to generate single, double, and triple mutant GFP-PCNA. (B) Wild-type and mutant GFP-PCNA localizes to the nucleus following transfection into PC-3 cells. Δ3Mu = Triple mutant (D86N/K110I/R146L). (C) PC-3 cells transfected with wild-type and mutant GFP-PCNA were treated for 8 h with 1 *μ*mol/L PCNA-I1 before separation of free (NP-E) and chromatin-associated (NP-R) pools of PCNA by nuclear fractionation. The samples were resolved by SDS-PAGE and analyzed by immunoblotting with antibodies to PCNA, Histone 3 and *α*-tubulin. Optical density of GFP-PCNA is normalized to respective loading control. (D) A schematic representation of the “linker” that is formed by PCNA-I1 at the interface of two PCNA monomers. Mutations interrupting the binding of PCNA-I1 to one monomer will interrupt the “linker” and prevent PCNA-I1-mediated reduction in PCNA association with chromatin.

The PCNA inhibitors identified in our previous study bind directly to PCNA trimer and render resistance of the trimer to separation by SDS-PAGE (Tan et al. 2012). This trimer stabilization is likely the cause of impaired loading of PCNA on to chromatin by RFC, effectively reducing PCNA association with chromatin (Tan et al. 2012). The in silico docking analysis predicted that PCNA-I1 binds at the interface of two PCNA monomers on the surface inside PCNA trimers (Tan et al. 2012). More specifically, PCNA-I1 is predicted to bind to Arg146 through and O–N hydrogen bond of one PCNA monomer and to Asp86 through a N–O hydrogen bond of the adjacent monomer. There is also a predicted strong nonpolar interaction between the lipophilic aroyl hydrazone of a naphthol on PCNA-I1 and carbons dominated by Lys110 of the adjacent monomer (Tan et al. 2012). To determine whether the predicted binding of PCNA-I1 to these amino acid residues is necessary for the effects of PCNA-I1 on chromatin association, we assessed the affects of PCNA-I1 on chromatin association of mutant PCNA. A GFP-PCNA fusion protein expression vector (GFP-PCNA-WT) was used to generate GFP-PCNA with mutations (Fig.1A), including (1) the single mutants GFP-PCNA-D86N, GFP-PCNA-K110I, and GFP-PCNA-R146L, (2) double mutants GFP-PCNA-D86N-K110L, GFP-PCNA-D86N-R146L, and GFP-PCNA-K110I-R146L, and (3) triple mutant GFP-PCNA-D86N-K110L-R146L. Twenty-four hours after transfection, the expression of GFP-PCNAs in PC-3 cells was confirmed under a fluorescent microscope, followed by treatment for 8 h with 1 *μ*mol/L PCNA-I1. Both wild-type and mutated GFP-PCNAs can localize to nucleus **(**Fig.1B**)** and associate to chromatin, which is revealed by their presence in the NP-40 extraction-resistant fraction (NP-R, GFP-PCNA in the upper panel of Figure1C**)**. Treatment with PCNA-I1 did not significantly alter expression levels of free form PCNA (both endogenous PCNA, Endo-PCNA, and engineered, GFP-PCNA), which is in the NP-E fraction **(**the lower panel of Fig.1C**)**. The chromatin-associated endogenous PCNA (Endo-PCNA, upper panel, Fig.1C**)**, wild-type GFP-PCNA, and GFP-PCNA with a single mutation at either D86 (D86N) or K110 (K110I) **(**Fig.1C, upper panel**)** is reduced by treatment with PCNA-I1, confirming our previous finding (Tan et al. 2012). In sharp contrast, the chromatin-associated GFP-PCNA with mutation at R146 (R146L) and those with double and triple mutations are not affected by the compound **(**Fig.1C, upper panel**)**. These results suggest that PCNA-I1 is very likely to serve as a “linker” at the monomer–monomer interface (Fig.1D). PCNA-I1 still can bind to two PCNA molecules and serve as the “linker” when either K110 or D86 (located in the same monomer) is mutated. When R146 or both K110 and D86 are mutated, PCNA-I1 can bind to only one monomer but not the adjacent one, and PCNA-I1 cannot bind triple-mutated GFP-PCNA. Therefore, the association with chromatin of these mutated PCNA is not affected by PCNA-I1.

### Selection of SAR compounds for further development

**Figure 2:**
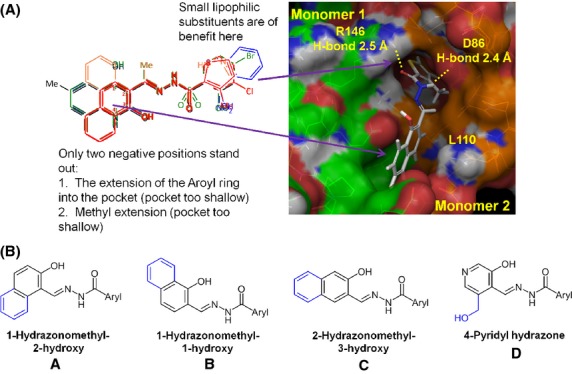
Selection of SAR compounds based on PCNA-Is structures. A. Overlay of nine PCNA-Is previously identified are color-coded from most active to least active, red → orange → green → blue → black. Areas of red/orange associated with better potency while areas of blue/black associated with less potency. Docking of PCNA-I1 at PCNA monomer interface and predicted interaction sites are also shown. B. SAR compound scaffold groups (A–D) which vary by the orientation of the naphthyl ring.

Having demonstrated that the predicted binding site is essential for the effects of PCNA-I1 on chromatin association, we performed an initial SAR analysis based on structure and activity of PCNA-I1 and other PCNA-Is reported in our previous manuscript (Tan et al. 2012). The image in Figure2A show the potential correlations between the structures of the compounds and their potencies in stabilizing PCNA trimer structure and in suppressing tumor cell growth reported previously (Tan et al. 2012). The potencies were color-coded from Red → Orange → Green → Blue → Black (most active to least active) and then overlaid with the hydrazones. This analysis suggested the essentiality of the hydroxynaphthyl and hydrazone subunits. To further SAR analysis and to identify compounds with properties superior to PCNA-I1 in PCNA trimer stabilization, cytotoxicity against cancer cells, and solubility, we selected 46 PCNA-I1 analogs for further investigation (Table1). As shown in Figure2B, the majority of analogs evaluated contained these substructures (scaffold A: 1-hydrazonomethyl-2-hydroxy; scaffold B: 2-hydrazonomethyl-1-hydroxy; scaffold C: 2-hydrazonomethyl-3-hydroxy; scaffold D: 4-pyridyl hydrazine) and sought to explore variations within isomeric presentations of these scaffolds and variations of the aroyl moiety.

**Table 1:**
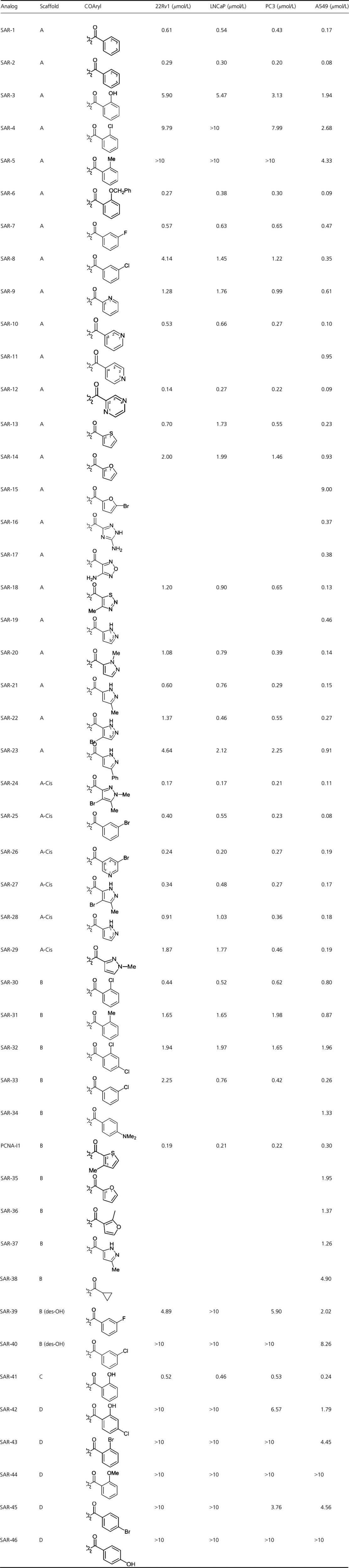
Classification of SAR compounds by scaffold group illustrating CoAryl and indicating IC_50_ in 22Rv1, LNCaP, PC-3, and A549 cells.

### Effects of SAR compounds on tumor cell growth and PCNA trimer stability

The growth inhibitory effects of the compounds were tested in four human cancer lines (three prostate cancer cell lines: LNCaP, 22Rv1, and PC-3 and one lung cancer cell line: A549). PCNA-I1 was included as a control for comparison of the potency. As shown in Table1, the four cell lines exhibit a fairly consistent and parallel sensitivity toward the SAR compounds, wherein IC_50_ values of growth inhibition are generally 22Rv1 >  LNCaP > PC-3 > A549 cells, and A549 cells are usually about one to threefold more sensitive than the other cell lines to the SAR compounds. Overall, the compounds with the scaffold 1-hydrazonomethyl-2hydroxy (Fig.2B, scaffold A,) show most potent inhibitory effects of tumor cell growth and 12 of 29 compounds in this group inhibit tumor cell growth with IC_50_ values around or below 0.3 *μ*mol/L (Table1). The SAR-24 is the most potent with IC_50_ of 0.165 ± 0.041 *μ*mol/L for the four cell lines.

**Figure 3:**
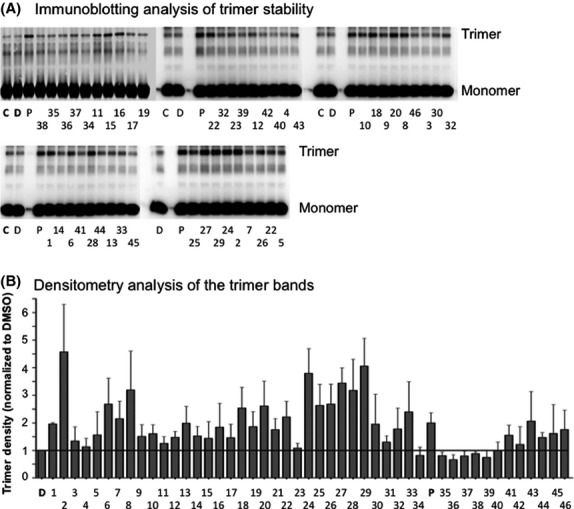
Effects of SAR compounds on PCNA trimer stability. (A) Stability of PCNA trimers upon treatment with SAR compounds. Purified recombinant His-PCNA was treated with 10 *μ*mol/L SAR compounds for 3 h at room temperature. The reaction was stopped by the addition of 2x Laemmli sample buffer without the reducing agent 2-mercaptoethanol. The samples were resolved by SDS-PAGE without boiling and analyzed by immunoblotting with PCNA antibody. B. Densitometry analysis of PCNA trimers from (A). Data shown are the average of three experiments ± standard deviation. Notes: C, buffer control; D, DMSO control; P, PCNA-I1; the numerical numbers under the figures represent SAR compound numbers.

Next we determined the effects of the SAR compounds on the resistance of PCNA trimers to separation to monomers by SDS-PAGE, using PCNA-I1 and DMSO as positive and negative controls, respectively. As shown in Figure3A and B, the SAR compounds from scaffold group A demonstrated variable effects on PCNA trimer stability. Notably, treatment with SAR-1, SAR-2, SAR-6, SAR-7, SAR-8, SAR-18, SAR-20, SAR-22, and SAR-24 showed more potent effects on PCNA trimer stability compared to other SAR compounds within scaffold group A. Overall, treatment of PCNA with SAR compounds from scaffold group A-cis resulted in significantly increased PCNA trimer stabilization. Of the eight SAR compounds in scaffold group B, SAR-32, SAR-33, and PCNA-I1 considerably increased the stability of the PCNA trimers. Several SAR compounds in scaffold groups B (des-OH) had no significant effects on PCNA trimer stability. Overall, SAR compounds from scaffold group C and D did not significantly increase trimer stability.

### Effects of SAR compounds of PCNA association with chromatin

**Figure 4:**
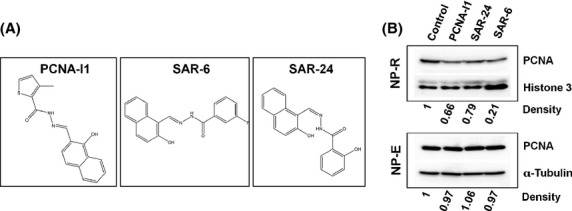
Selected SAR compounds reduced chromatin-associated PCNA. (A) The chemical structure of PCNA-I1, SAR-6, and SAR-24. (B) The effects of PCNA-I1, SAR-6, and SAR-24 on chromatin-associated PCNA. PC-3 cells were treated for 8 h with 1 *μ*mol/L PCNA-I1, SAR-6, and SAR-24 before separation of free (NP-E) and chromatin-associated (NP-R) pools of PCNA by nuclear fractionation. The samples were resolved by SDS-PAGE and analyzed by immunoblotting with antibodies against PCNA, Histone 3 and *α*-tubulin. Optical density of PCNA is normalized to respective loading control.

To validate that inhibition of cell growth by the SAR compounds is associated with their effects on PCNA in cells, the effects of SAR-6 and SAR-24 on PCNA association with chromatin were investigated. These compounds, SAR-6 and SAR-24, were selected based on their potent inhibitory effects on tumor cell growth, PCNA trimer stabilization (Table1 and Fig.3), and potentially improved solubility (cLogP). PC-3 cells were treated with 1 *μ*mol/L SAR-6 and SAR-24 for 8 h. Cells treated with 1 *μ*mol/L PCNA-I1 were used as a control. As shown in Figure4, PCNA-I1 reduced the chromatin-associated PCNA in PC-3 cells, confirming our previous observation (Tan et al. 2012). Similarly, SAR-6 and SAR-24 also reduced chromatin-associated PCNA in PC-3 cells (Fig.4).

### Correlation between the effects of the SAR compounds on PCNA trimer stability and growth inhibition

**Figure 5:**
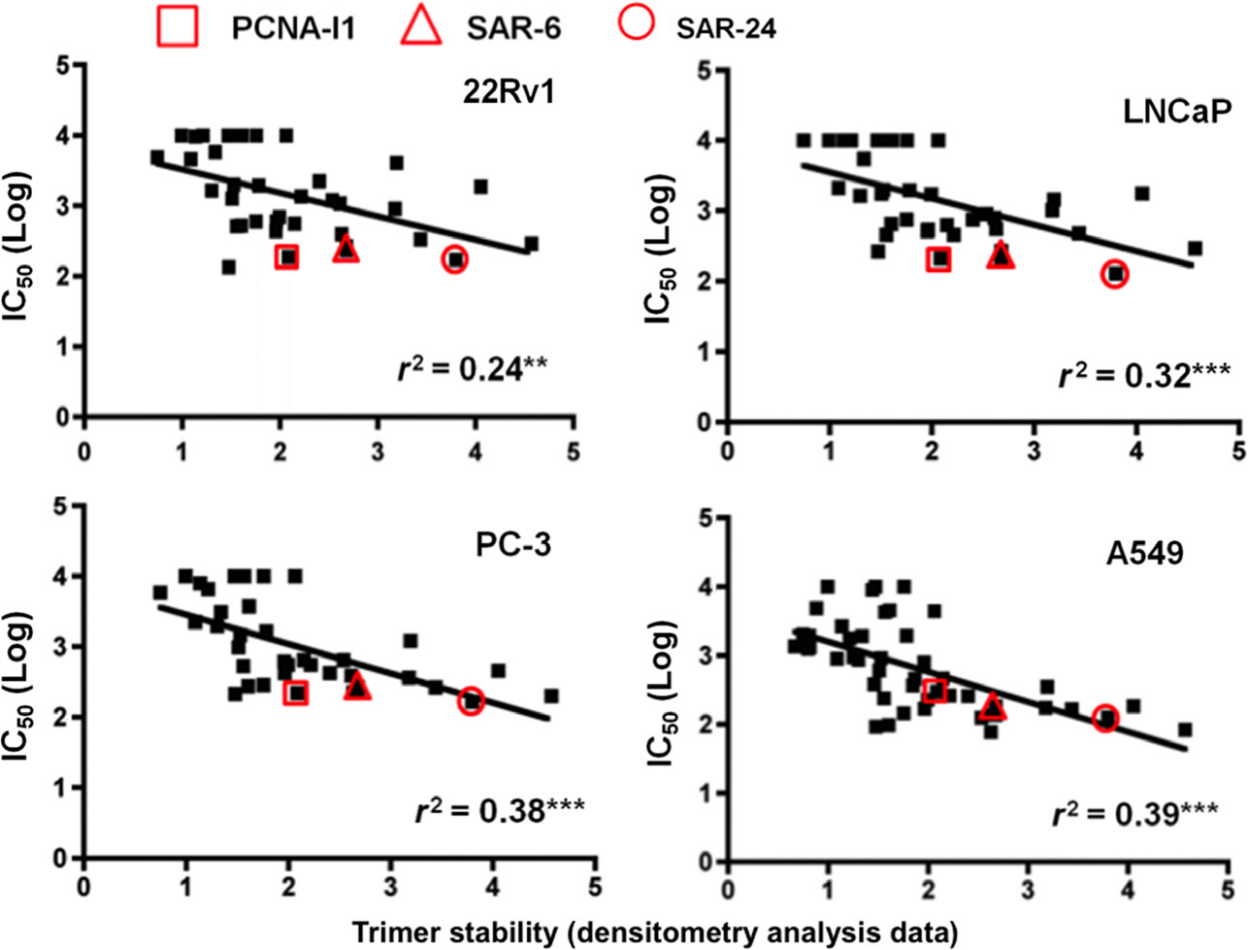
Correlation between growth inhibitory effects (IC_50_, nmol/L) of SAR compounds and trimer stability determined by linear regression analysis, r^2^ values are indicated as significantly different from zero by **P < 0.01 and ***P < 0.001. The location of PCNA-I1, SAR-6, and SAR-24 are indicated on the plot.

Linear regression analysis indicates a modest correlation between the effects of the SAR compounds (also PCNA-I1) on PCNA trimer stability and their growth inhibitory effects (Fig.5). In general, however, those compounds that significantly increased PCNA trimer stability were most potent at inhibiting tumor cell growth. Six SAR compounds, SAR-2, SAR-6, SAR-7, SAR-24, SAR-25, SAR-26, and SAR-27, had similar or stronger effects on tumor cell growth and PCNA trimer stability when compared to PCNA-I1 (Table1 and Fig.5).

## Discussion

Previously we reported a series of novel small molecule compounds that bind directly to and stabilize PCNA homotrimers, reduce chromatin-associated PCNA in cells, attenuate DNA replication, and selectively inhibit tumor cell growth (Tan et al. 2012). In this study, we further investigated the effects of PCNA-I1, the most potent among the PCNA-Is, on chromatin-associated PCNA in cells transiently transfected with wild-type and mutant GFP-PCNA. Moreover, based an initial SAR analysis, we identified 46 analogs of PCNA-I1 and investigated and compared their effects on PCNA trimer stabilization, tumor cell growth, and chromatin-associated PCNA with PCNA-I1.

We found that a short treatment (8 h) of GFP-PCNA-engineered PC-3 cells with PCNA-I1 reduced chromatin-associated endogenous PCNA, confirming our previous observation (Tan et al. 2012). The levels of chromatin-associated wild-type GFP-PCNA and single mutant of GFP-PCNA-D86N and GFP-PCNA-K110I, which are located in the same monomer in the predicted binding site, were also reduced upon exposure to PCNA-I1. In sharp contrast, the levels of chromatin-associated GFP-PCNA with other amino acid mutations (single mutation of GFP-PCNA-R146L, all double mutation, and triple mutation) were not affected. These data provide strong evidence that the reduction in chromatin-associated PCNA by PCNA-I1 is caused by direct interaction of PCNA-I1 with PCNA through these three amino acid residues. Although remaining to be elucidated by further studies, it is likely that the binding of PCNA-I1 to PCNA trimers at monomer–monomer interface stabilizes the trimer structure and attenuates PCNA loading to DNA by RFC (Fukuda et al. 1995; Bowman et al. 2004; Hedglin et al. 2013).

**Figure 6:**
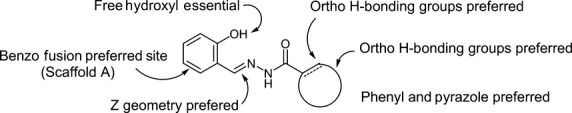
Summary of SAR analysis.

Among the most potent stabilizers of PCNA (>2-fold vs. DMSO), all of the compounds showed potent cytotoxic activity. The strongest PCNA trimer stabilization and cytotoxicity was confined to scaffolds A and B, including PCNA-I1, which belongs to the Scaffold B class. Over 40% of the analogs with scaffold A showed trimer stabilization greater than twofold and over 20% showed trimer stabilization greater than threefold, whereas only 20% of analogs with scaffold B showed trimer stabilization greater than twofold and none showed trimer stabilization greater than threefold, suggesting potency advantages within scaffold A. Strongest cytotoxicity was also observed within analogs based on scaffold A. It must be noted, however, that the scaffold A analogs with the most potent trimer stabilization and cytotoxicity do not have direct counterparts within scaffold B analogs. For pairwise comparisons, SAR-3 to SAR-30 (o-ClPh), SAR-4 to SAR-31 (o-MePh), and SAR-7 to SAR-33 (m-ClPh), where the aryl group is phenyl, had quite similar activity, whereas heteroaryl pairings of SAR-13 to SAR-35 (furan) and SAR-20 to SAR-37 (Me-pyrazole) showed a consistent potency advantage within scaffold A analogs. Within scaffold A analogs, all of the Z isomers showed trimer stabilization greater than 2.5-fold and consistently better potency in growth inhibition against the four cell lines, suggesting superiority of this isomer relative to the E isomers. However, pairwise comparisons of SAR-2 to SAR-24 (o-HOPh), SAR-7 to SAR-25 (m-ClPh and m-BrPh), and SAR-18 to SAR-28 (pyrazole), showed quite similar potencies, although SAR-23 to SAR-27 (Br,MePyrazole) indicates far more potent trimer stabilization (3.44 vs. 1.09-fold) and overall four to 13-fold more potent cytotoxicity in the Z-isomer. Across this series, compounds with an ortho hydroxyphenyl (SAR-2 and 24) showed the best combination of PCNA stabilization and potent cytotoxicity. Compounds across both isomeric scaffold A analogs generally had greater activity when a hydrogen bond donor is ortho to the aroyl carbonyl as in analogs SAR-2, 18, 20, 22, 24, 27, and 28. Similarly, analogs with meta hydrophobic substituents also showed stronger potencies, as seen in analogs SAR-6, 7, 25, 26, and 33. Typically, E and Z isomers are quite different in shape and conformation, so this overall similar activity is somewhat surprising and may be caused by some isomerization of the compounds occurred during storage (Cordier et al. 2004). The absence of substantial stabilization of PCNA trimers in analogs of scaffold D and the des-OH analogs SAR-39 and SAR-40 are consistent with the above hypothesized essentiality of the hydroxynaphthyl substructure. Only one analog with Scaffold C (SAR-41) was available. SAR-41 showed submicromolar cytotoxicity and relatively moderate activity in trimer stabilization. The results of SAR analysis are summarized in Figure6.

There exists a modest correlation between PCNA stabilization and cell growth inhibition induced by the 46 PCNA-I1 analogs. All compounds with strong PCNA stabilization showed potent cytotoxicity across the four cell lines. However, several compounds showed high potency in stabilizing PCNA trimers, but demonstrated moderate growth inhibition in one or two cell lines. One plausible interpretation for this discrepancy is that the trimer stability assay reveals the direct interaction of the compounds with PCNA trimers in vitro, whereas the cell growth inhibition is a much more complex process, including penetration, accumulation, and metabolism of the compounds in the cells. On the other hand, the modest correlation is also caused in part by several compounds with relatively potent growth inhibitory effects but moderate trimer stabilization activity. Since hydroxyaryl hydrazones display a number of activities, some of which are cytotoxic or mechanistically associated with cytotoxicity (Saletta et al. 2010; Caboni et al. 2013; Stefani et al. 2013; Naveen Kumar et al. 2014), it is also plausible that non-PCNA pathways would contribute to the growth inhibitory effects of these SAR compounds. Overall, this study led to the identification of 14 compounds with superior PCNA stabilization and inhibition of tumor cell growth to PCNA-I1, and the most potent analogs from the scaffold A class.

Two compounds, SAR-6 and SAR-24 were advanced to detailed chromatin associations studies, due to their superior properties to PCNA-I1 in suppressing tumor cell growth, increasing trimer stability, and potentially improved solubility (cLogP). Treatment of PC-3 cells with SAR-6 and SAR-24 decreased PCNA association with chromatin, which coincides with the IC_50_ and trimer stability for these two compounds, thus confirming the mechanism of action for this class of compounds.

Great efforts have been made to identify peptides and small molecules that can interrupt the interaction of PCNA with partner proteins. Several peptides mimicking the PIP-box and APIM have been developed (Luo et al. 1995; Gilljam et al. 2009; Bozza et al. 2012; Muller et al. 2013). These peptides were shown to inhibit cell proliferation, induce apoptosis, and enhance cytotoxicity of some chemotherapy drugs in culture (Luo et al. 1995; Gilljam et al. 2009; Bozza et al. 2012; Muller et al. 2013). Tumor cells were much more susceptible to the cytotoxic activity of the peptides (Luo et al. 1995; Gilljam et al. 2009; Bozza et al. 2012; Muller et al. 2013), which is consistent with our observation reported previously (Tan et al. 2012). Moreover, the peptide mimicking APIM, although not effective alone, was shown to enhance therapeutic effects of melphalan in a myeloma model in mice (Muller et al. 2013). Recently, a small molecule compound that interrupts PCNA interaction with PIP-box containing protein partners was identified (Punchihewa et al. 2012; Actis et al. 2013). This compound was shown to enhance DNA damage and cytotoxicity induced by cisplatin and inhibit DNA repair (Punchihewa et al. 2012; Actis et al. 2013). It, however, only produced moderate growth inhibitory effects (Punchihewa et al. 2012). These peptides and small molecule induce cytotoxicity or inhibit tumor growth at micromolar concentrations and are less potent in comparison with the PCNA-Is identified in our studies. One plausible reason for the discrepancy in potency between these peptides and small molecule and our PCNA-Is is that unlike the PCNA-Is from our studies, which reduce chromatin-associated PCNA and will potentially attenuate most functions of PCNA, these peptides and small molecule target one of the multiple interaction sites in PCNA and, hence, will only interrupt partial functions of PCNA.

In conclusion, we have validated that PCNA-I1 targets PCNA and discovered several PCNA-I1 analogs superior to PCNA-I1 in stabilizing PCNA trimer structure and inhibiting tumor cell growth. The SAR analysis shows the essentiality of the hydroxynaphthyl substructure in the compounds. Overall, the information gathered in this study has led to the identification of SAR-24, which is denoted as PCNA-I1S for its superior potencies and potentially improved solubility, and will be used for future development of PCNA-targeting cancer therapy studies.

## Abbreviations

GFP: green fluorescent protein
NP-R: NP-40 resistant
NP-E: NP-40 extractable
PMSF: phenylmethylsulfonyl fluoride
NMR: nuclear magnetic resonance
IDCL: interdomain connecting loop
APIM: AlkB homologue 2 PCNA-interacting motif
FBS: fetal bovine serum
MTT: 3-(4,5-dimethylthiazol-2-yl)-2,5-diphenyltetrazolium bromide
PCNA: proliferating cell nuclear antigen
PCNA-I: PCNA inhibitor
PIP: PCNA-interaction protein
RFC: replication factor C
SAR: structure–activity relationship

## Supporting Information

**Data S1.** NMR and MS analyses of compounds used in the studies.
