# Erratum

**DOI:** 10.1002/prp2.167

**Published:** 2015-07-24

In Dillehay et al. 2015, an error has been identified in Table1. The COAryl schematic diagrams mismatched the corresponding Analogs for SAR-1 to SAR-25. The correct table is shown below:

**Table 1:** Classification of SAR compounds by scaffold group illustrating CoAryl and indicating IC50 in 22Rv1, LNCaP, PC-3, and A549 cells

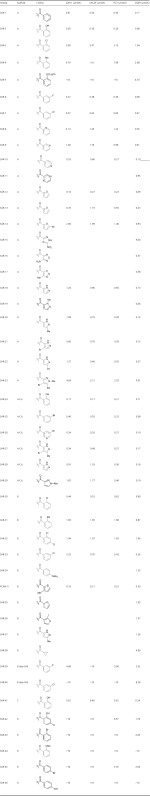

We apologize for this error.
