# Supplementary material for: Target validation and structure–activity analysis of a series of novel PCNA inhibitors
Source: Pharmacol Res Perspect. 2015 Feb 2;3(2):e00115. doi: 10.1002/prp2.115 (PMC4324689; doi:10.1002/prp2.115)

PCNA-I1

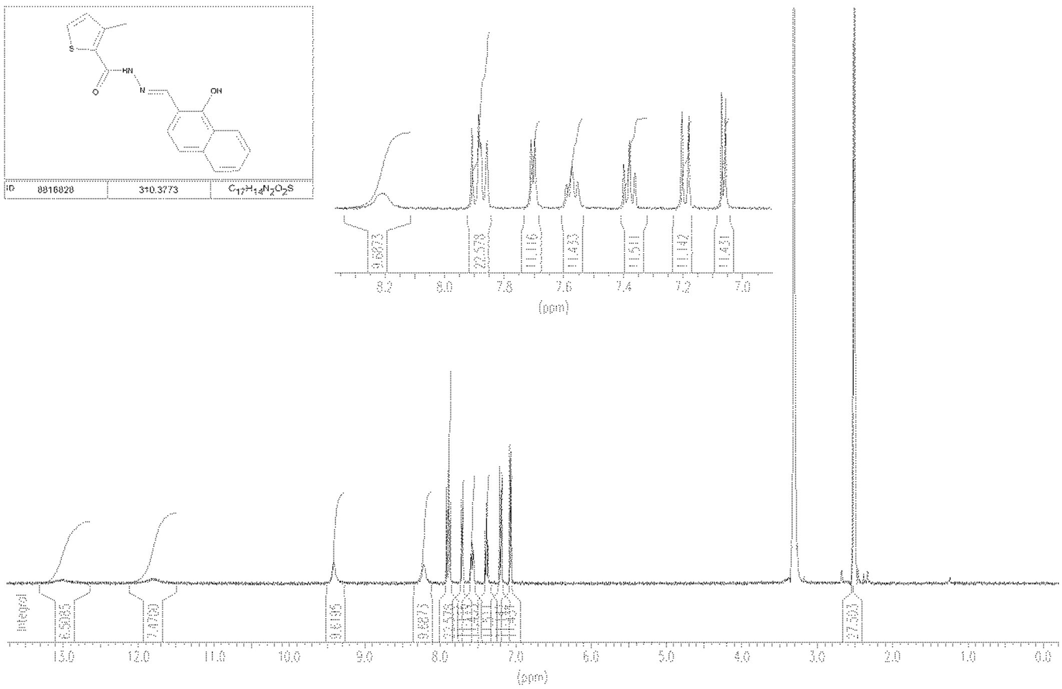

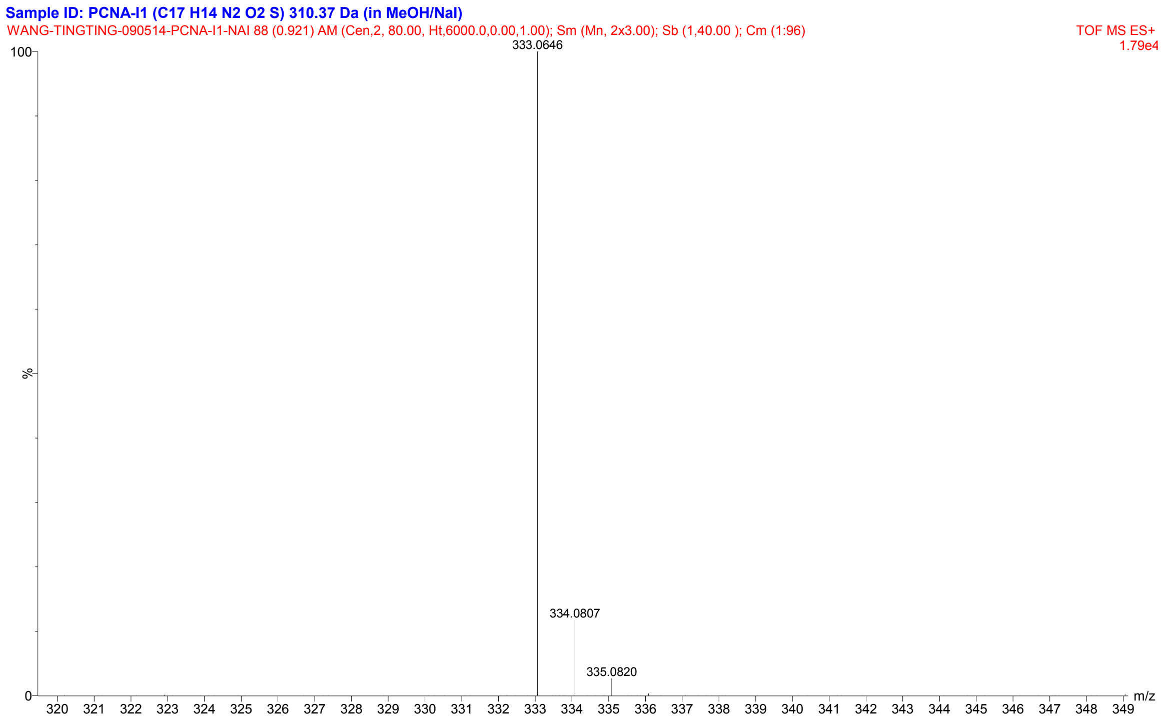

Sample ID: PCNA-I1 (C17 H14 N2 O2 S) 310.37 Da (in MeOH/NaI)
WANG-TINGTING-090514-PCNA-I1-NAI 88 (0.921) AM (Cen,2, 80.00, Ht,6000.0,0.00,1.00); Sm (Mn, 2x3.00); Sb (1,40.00); Cm (1:96)
TOF MS ES+
1.79e4
333.0646
334.0807
335.0820
%
m/z

SAR-2

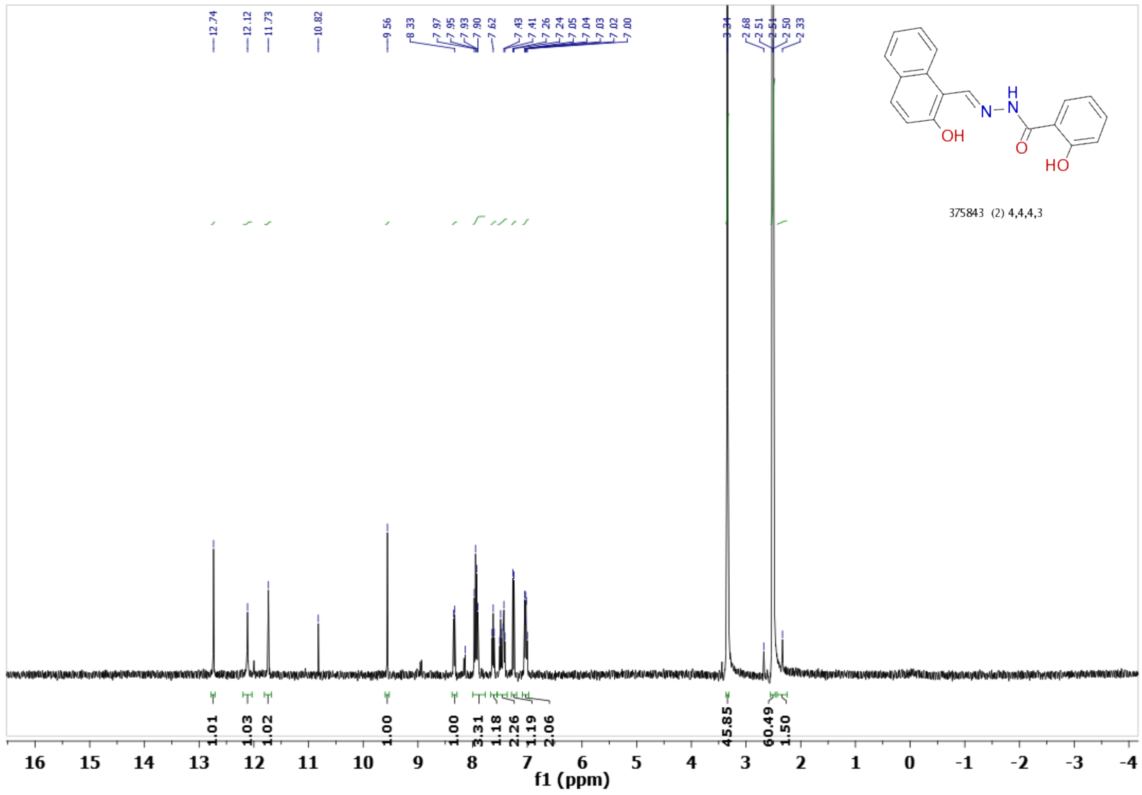

Sample ID: 375843 (C18 H14 N2 O3) 306.31 Da (in MeOH/ESIB)

WANG-TINGTING-090214-375843-R2 62 (0.650) AM (Cen,2, 80.00, Ht,6000.0,0.00,1.00); Sm (Mn, 2x3.00); Sb (1,40.00); Cm (1:96)

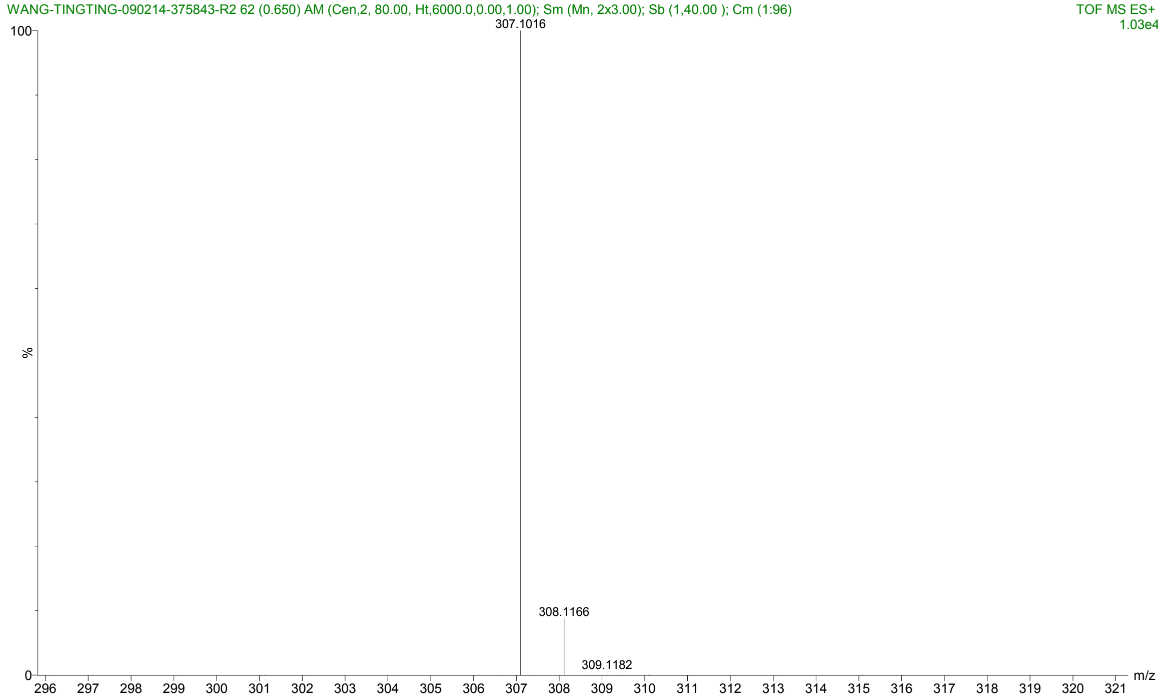

SAR-3

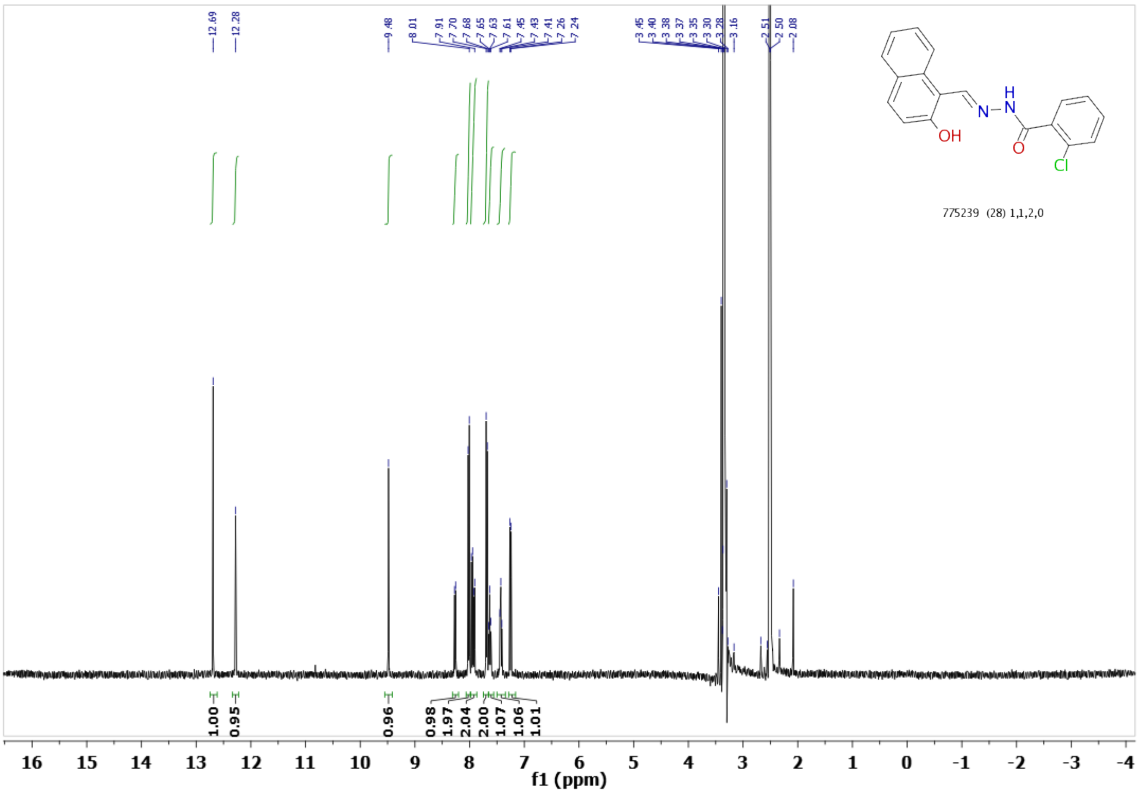

12.69
12.28
9.48
8.01
7.91
7.70
7.68
7.65
7.63
7.61
7.45
7.43
7.41
7.26
7.24
3.45
3.40
3.38
3.37
3.35
3.30
3.28
3.16
2.51
2.50
2.08
OH
H
N
N
O
Cl
775239 (28) 1,1,2,0
1.00
0.95
0.96
0.98
1.97
2.04
2.00
1.07
1.06
1.01
16 15 14 13 12 11 10 9 8 7 6 5 4 3 2 1 0 -1 -2 -3 -4
f1 (ppm)

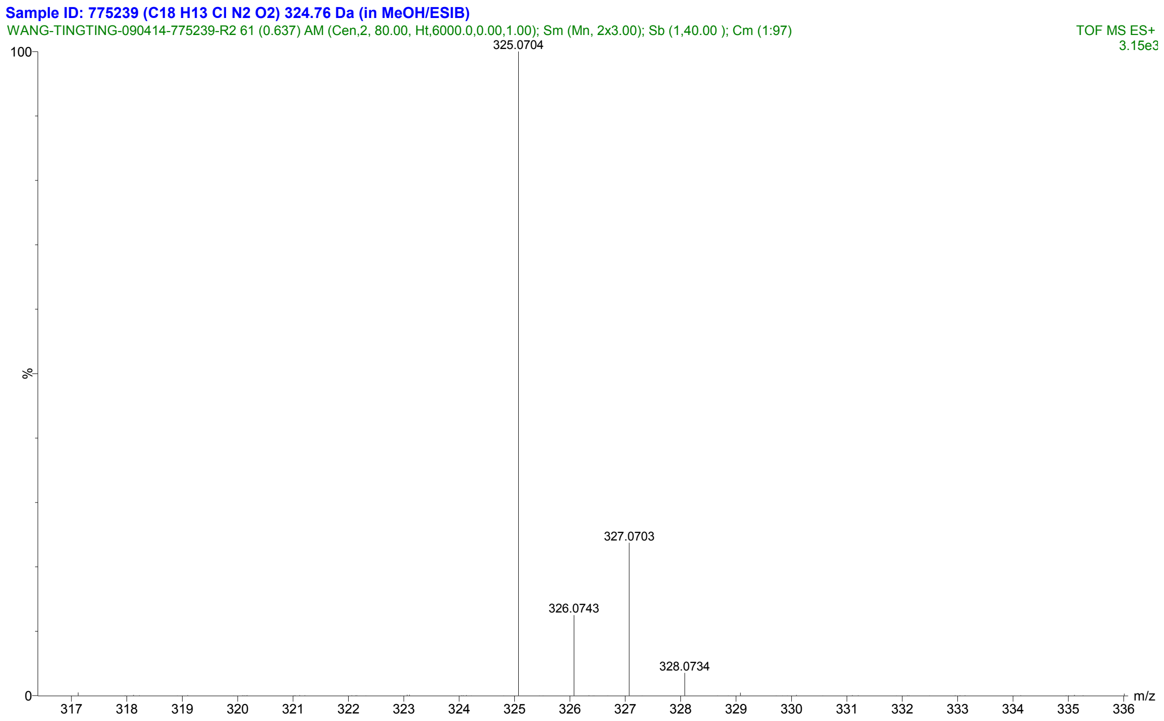

Sample ID: 775239 (C18 H13 Cl N2 O2) 324.76 Da (in MeOH/ESIB)
WANG-TINGTING-090414-775239-R2 61 (0.637) AM (Cen,2, 80.00, Ht,6000.0,0.00,1.00); Sm (Mn, 2x3.00); Sb (1,40.00); Cm (1:97)
TOF MS ES+
3.15e3
100
%
0
325.0704
326.0743
327.0703
328.0734
317 318 319 320 321 322 323 324 325 326 327 328 329 330 331 332 333 334 335 336
m/z

SAR-4

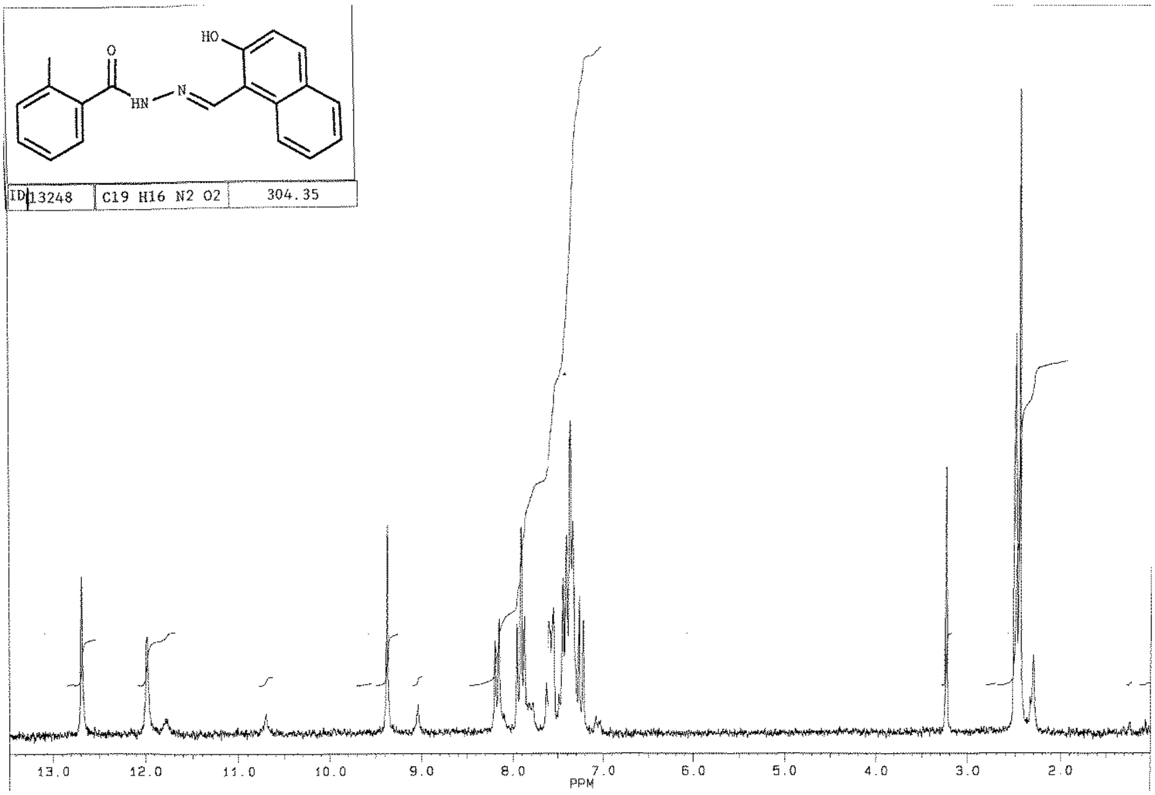
HO
O
HN
N
ID 13248
C19 H16 N2 O2
304.35
13.0
12.0
11.0
10.0
9.0
8.0
7.0
6.0
5.0
4.0
3.0
2.0
PPM

SAR-5

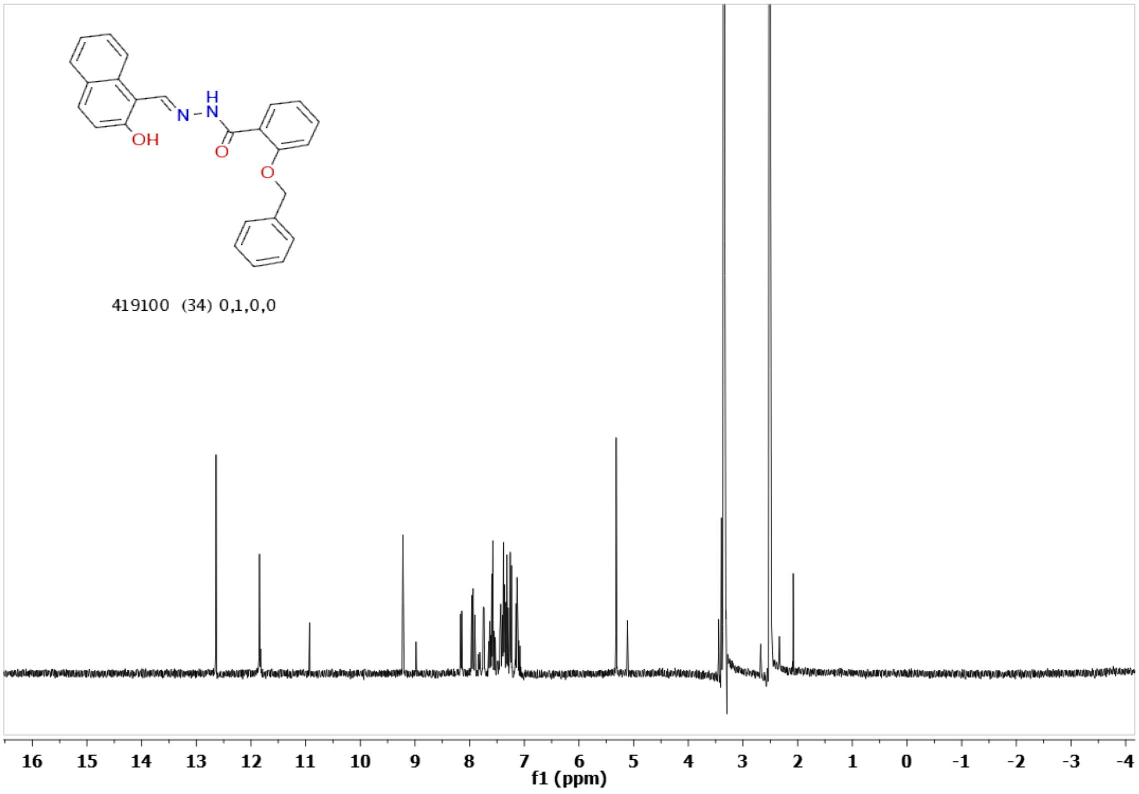

H
N
N
OH
O
O
419100 (34) 0,1,0,0
16 15 14 13 12 11 10 9 8 7 6 5 4 3 2 1 0 -1 -2 -3 -4
f1 (ppm)

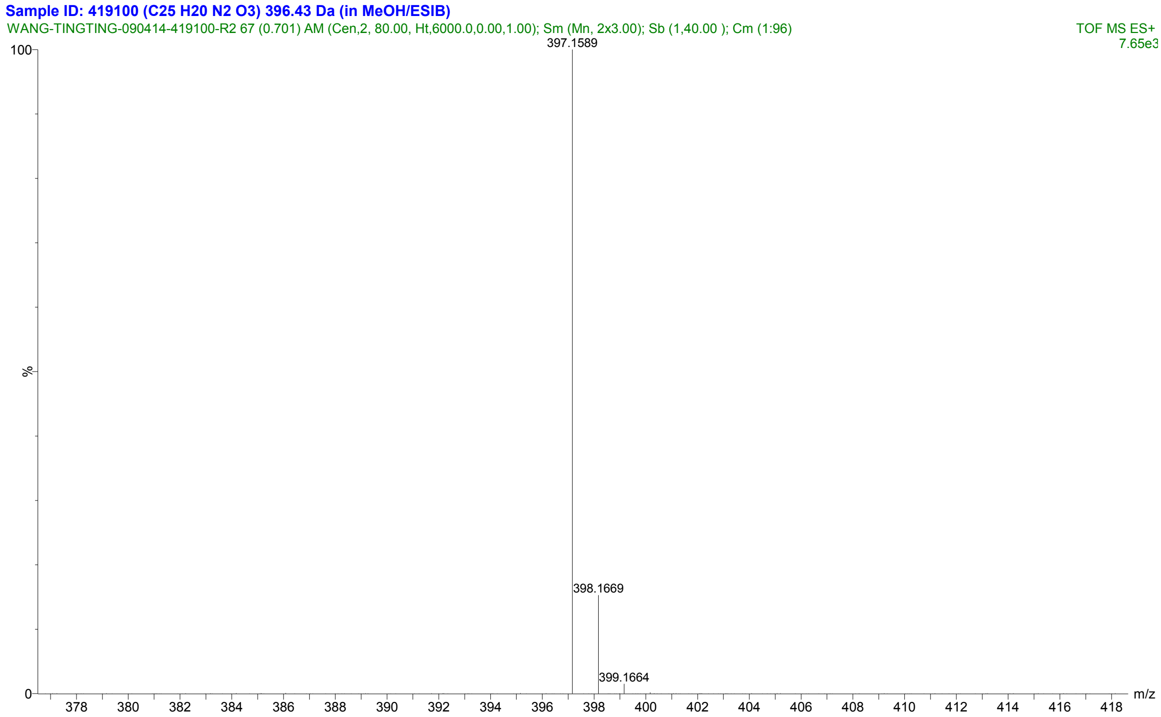

Sample ID: 419100 (C25 H20 N2 O3) 396.43 Da (in MeOH/ESIB)
WANG-TINGTING-090414-419100-R2 67 (0.701) AM (Cen,2, 80.00, Ht,6000.0,0.00,1.00); Sm (Mn, 2x3.00); Sb (1,40.00); Cm (1:96)
TOF MS ES+
7.65e3
100
%
0
397.1589
398.1669
399.1664
378 380 382 384 386 388 390 392 394 396 398 400 402 404 406 408 410 412 414 416 418
m/z

SAR-6

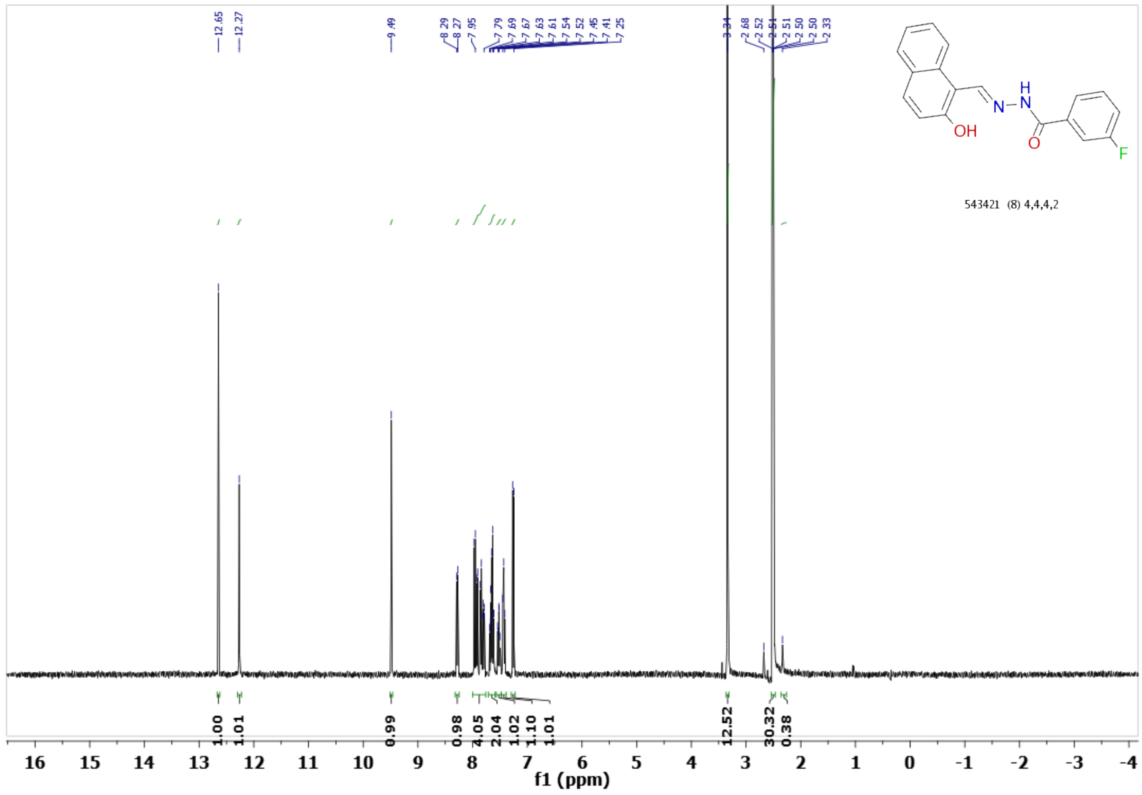

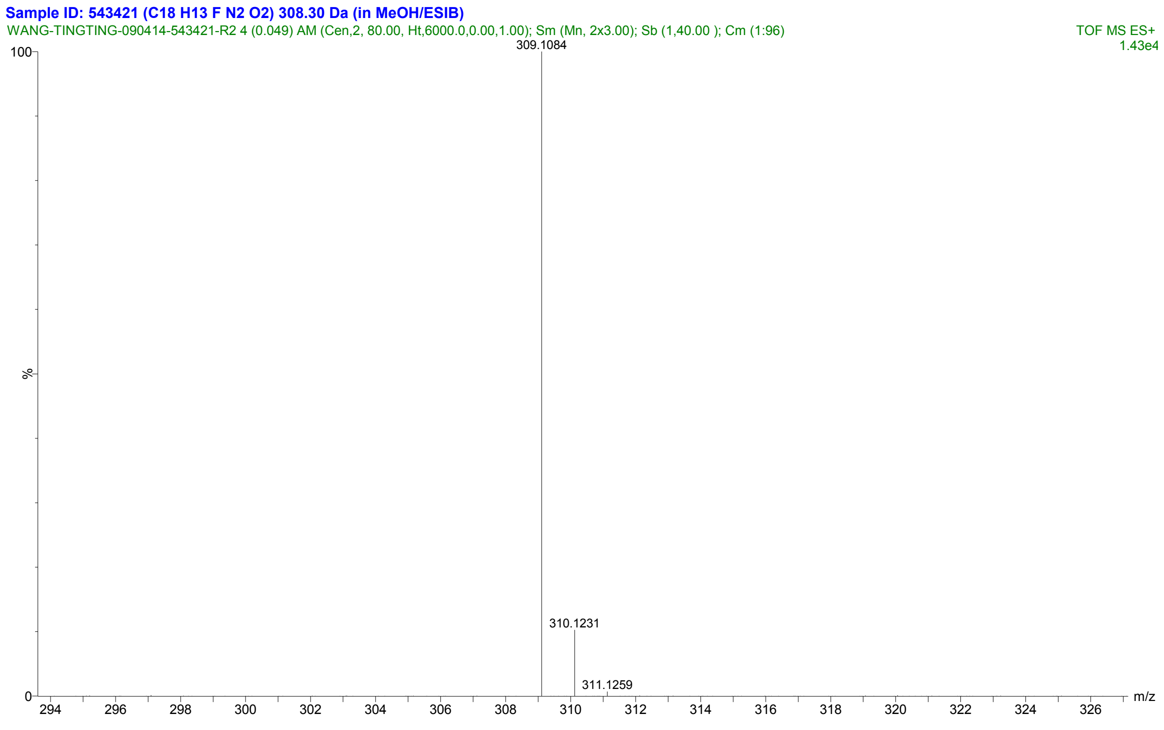
Sample ID: 543421 (C18 H13 F N2 O2) 308.30 Da (in MeOH/ESIB)
WANG-TINGTING-090414-543421-R2 4 (0.049) AM (Cen,2, 80.00, Ht,6000.0,0.00,1.00); Sm (Mn, 2x3.00); Sb (1,40.00); Cm (1:96)
TOF MS ES+
1.43e4
309.1084
310.1231
311.1259
m/z

SAR-7

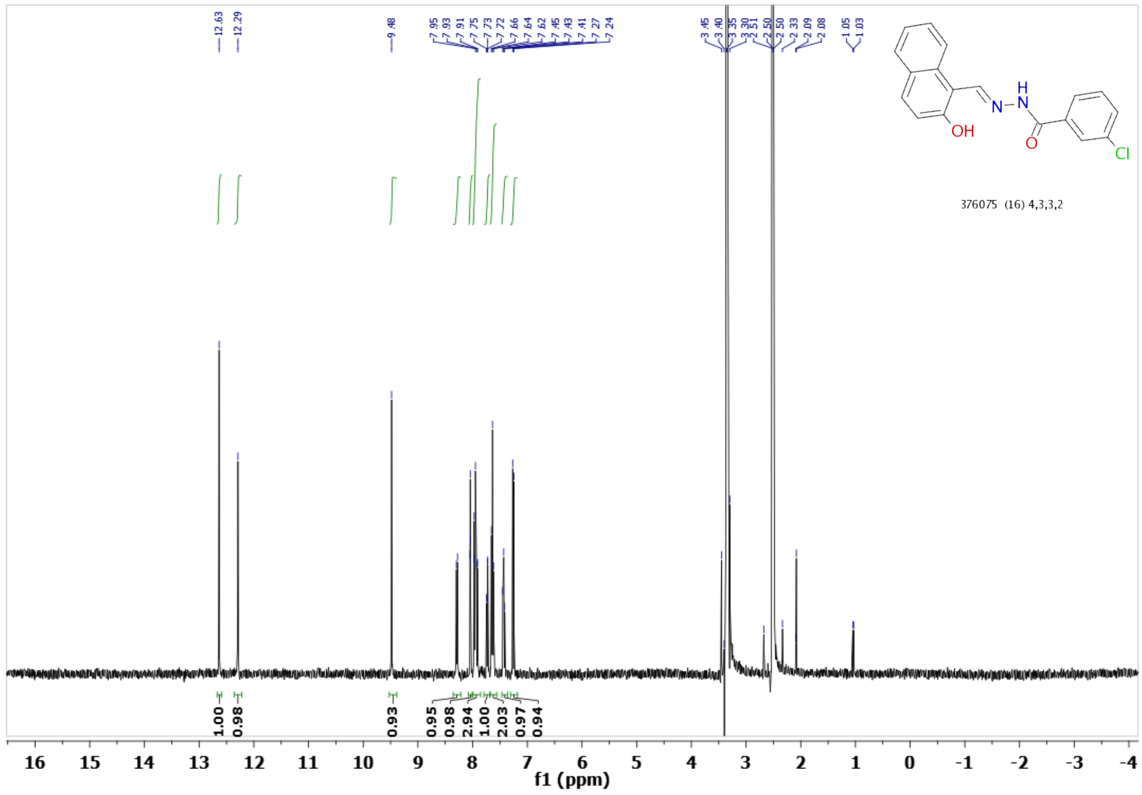

376075 (16) 4,3,3,2
f1 (ppm)

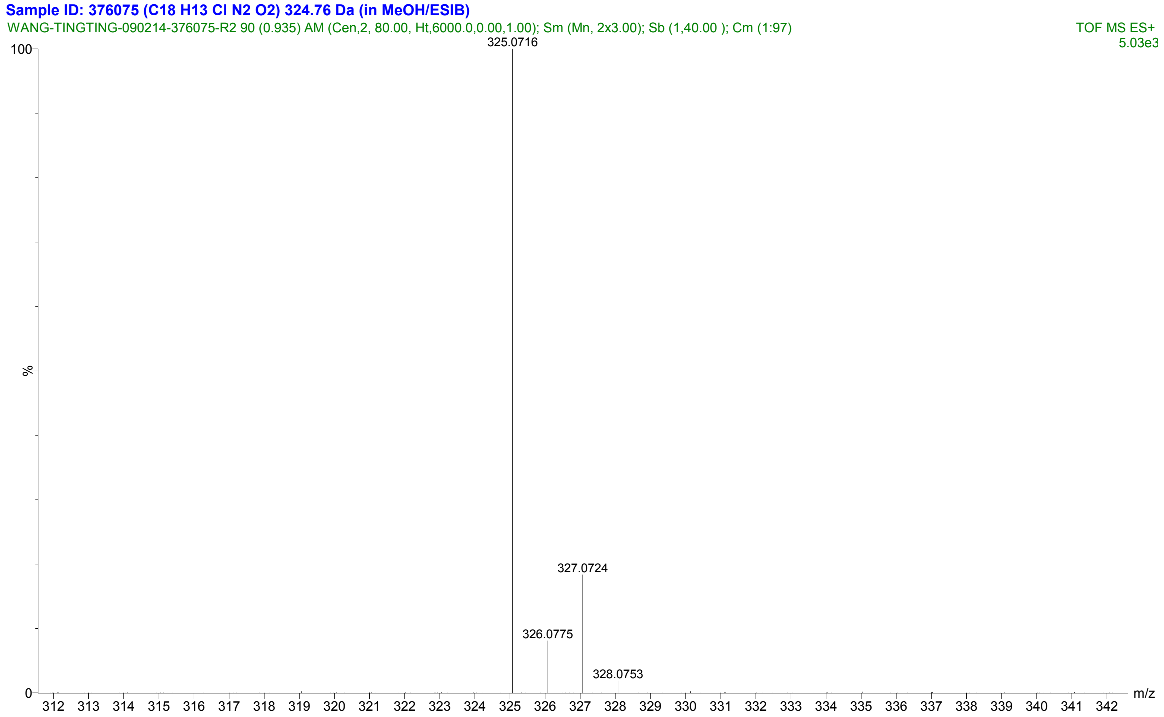

Sample ID: 376075 (C18 H13 Cl N2 O2) 324.76 Da (in MeOH/ESIB)
WANG-TINGTING-090214-376075-R2 90 (0.935) AM (Cen,2, 80.00, Ht,6000.0,0.00,1.00); Sm (Mn, 2x3.00); Sb (1,40.00); Cm (1:97)
TOF MS ES+
5.03e3
325.0716
327.0724
326.0775
328.0753
m/z

SAR-8

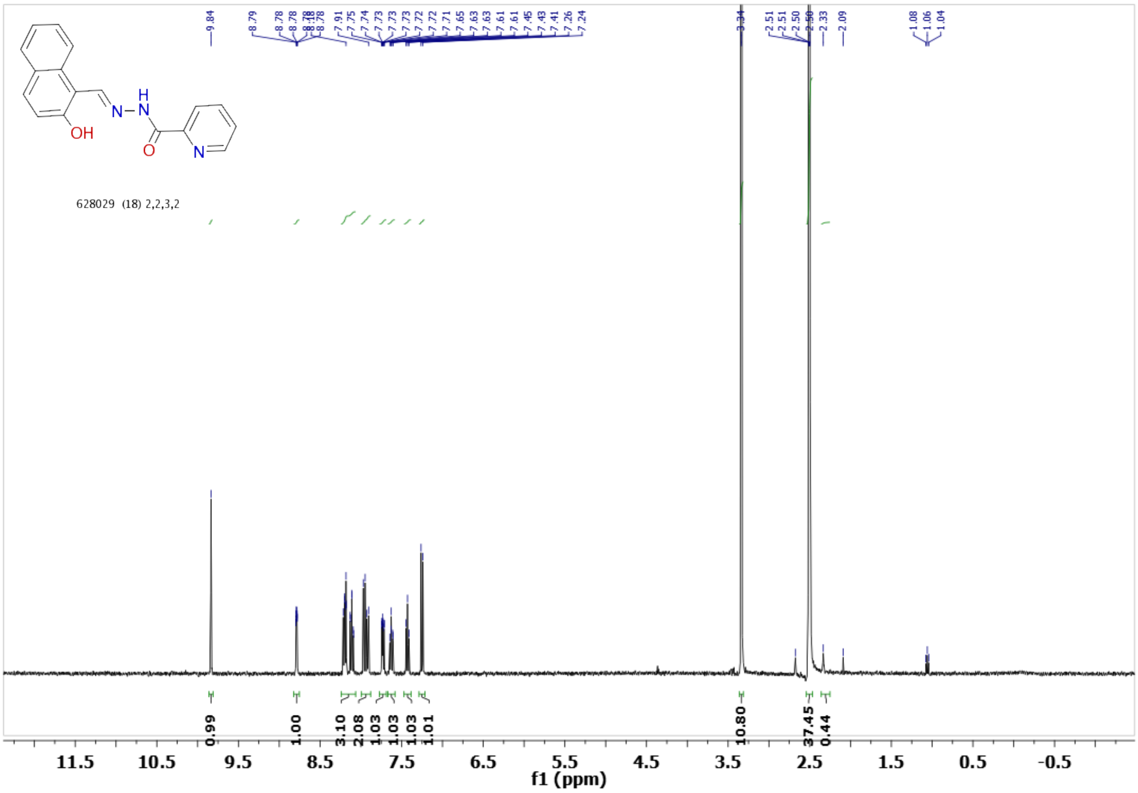

628029 (18) 2,2,3,2
OH
O
N
H
N–N
9.84
8.79
8.78
8.78
8.78
8.78
7.91
7.75
7.74
7.73
7.73
7.73
7.72
7.72
7.71
7.65
7.63
7.63
7.61
7.61
7.45
7.43
7.41
7.26
7.24
3.34
2.51
2.51
2.50
2.50
2.33
2.09
1.08
1.06
1.04
0.99
1.00
3.10
2.08
1.03
1.03
1.03
1.01
10.80
37.45
0.44
11.5
10.5
9.5
8.5
7.5
6.5
5.5
4.5
3.5
2.5
1.5
0.5
-0.5
f1 (ppm)

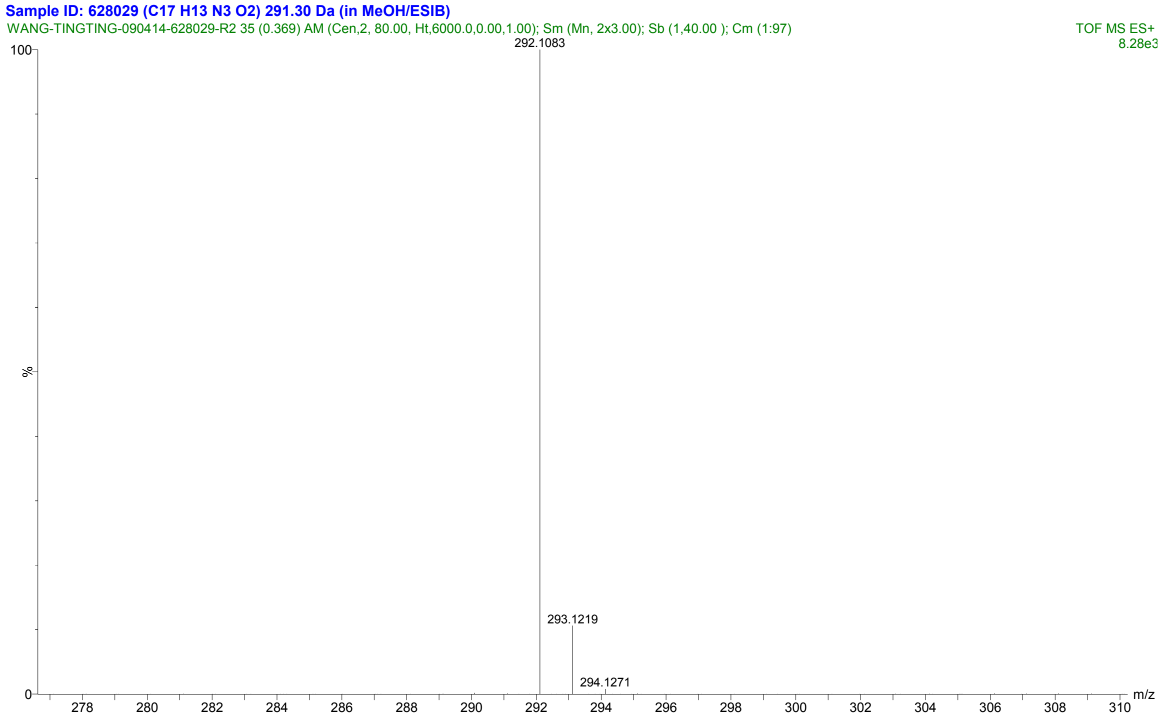

Sample ID: 628029 (C17 H13 N3 O2) 291.30 Da (in MeOH/ESIB)
WANG-TINGTING-090414-628029-R2 35 (0.369) AM (Cen,2, 80.00, Ht,6000.0,0.00,1.00); Sm (Mn, 2x3.00); Sb (1,40.00); Cm (1:97)
TOF MS ES+
8.28e3
100
%
0
292.1083
293.1219
294.1271
278
280
282
284
286
288
290
292
294
296
298
300
302
304
306
308
310
m/z

SAR-9

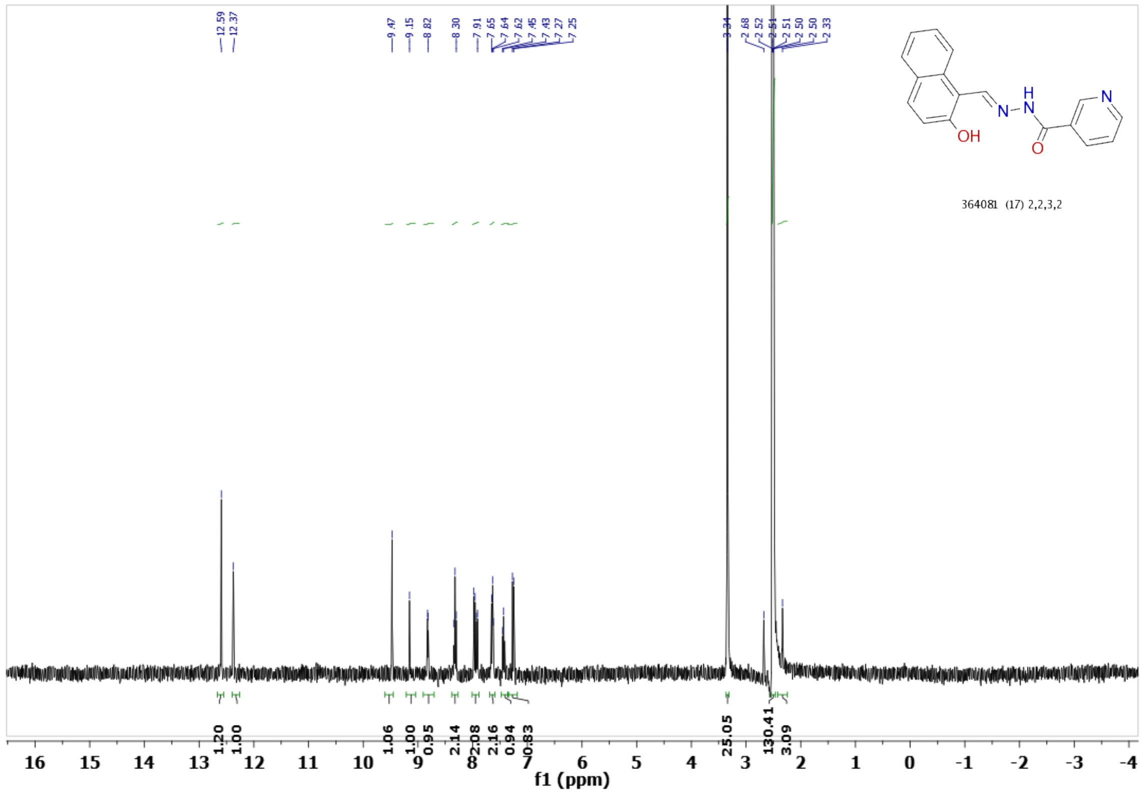

12.59
12.37
9.47
9.15
8.82
8.30
7.91
7.65
7.64
7.62
7.45
7.43
7.27
7.25
3.34
2.68
2.52
2.51
2.51
2.50
2.50
2.33
OH
H
N
N
N
O
364081 (17) 2,2,3,2
1.20
1.00
1.06
1.00
0.95
2.14
2.08
2.16
0.94
0.83
25.05
130.41
3.09
f1 (ppm)

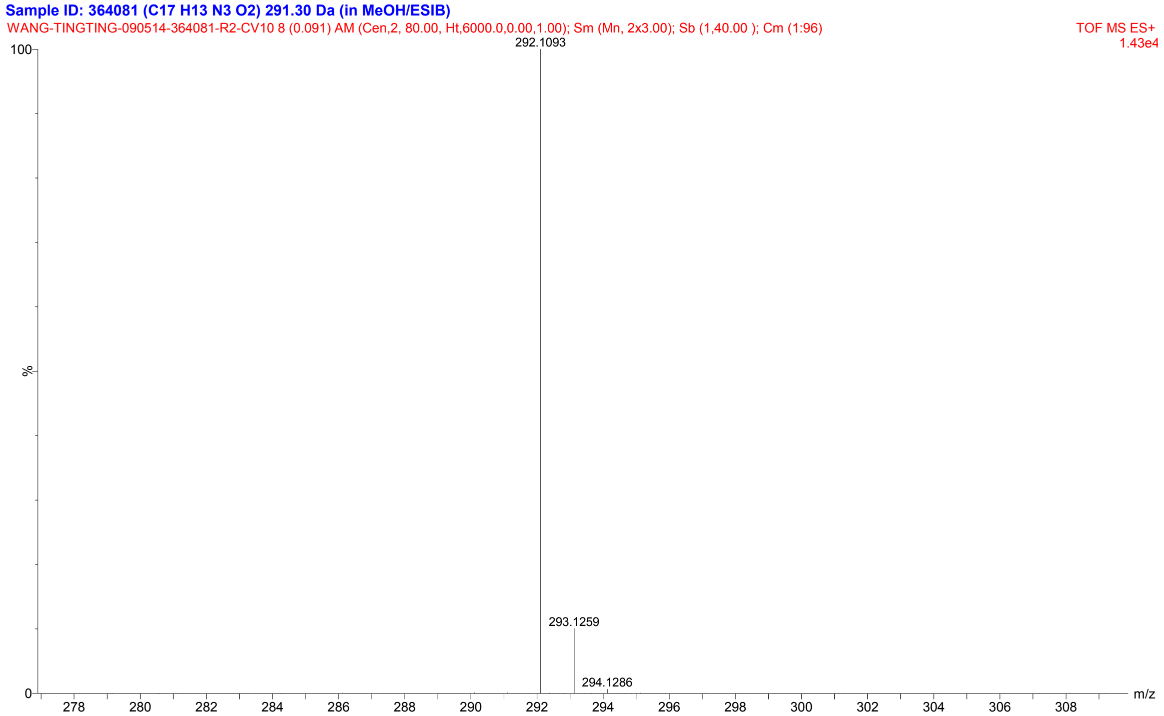

Sample ID: 364081 (C17 H13 N3 O2) 291.30 Da (in MeOH/ESIB)
WANG-TINGTING-090514-364081-R2-CV10 8 (0.091) AM (Cen,2, 80.00, Ht,6000.0,0.00,1.00); Sm (Mn, 2x3.00); Sb (1,40.00); Cm (1:96)
TOF MS ES+
1.43e4
292.1093
293.1259
294.1286
%
m/z

SAR-11

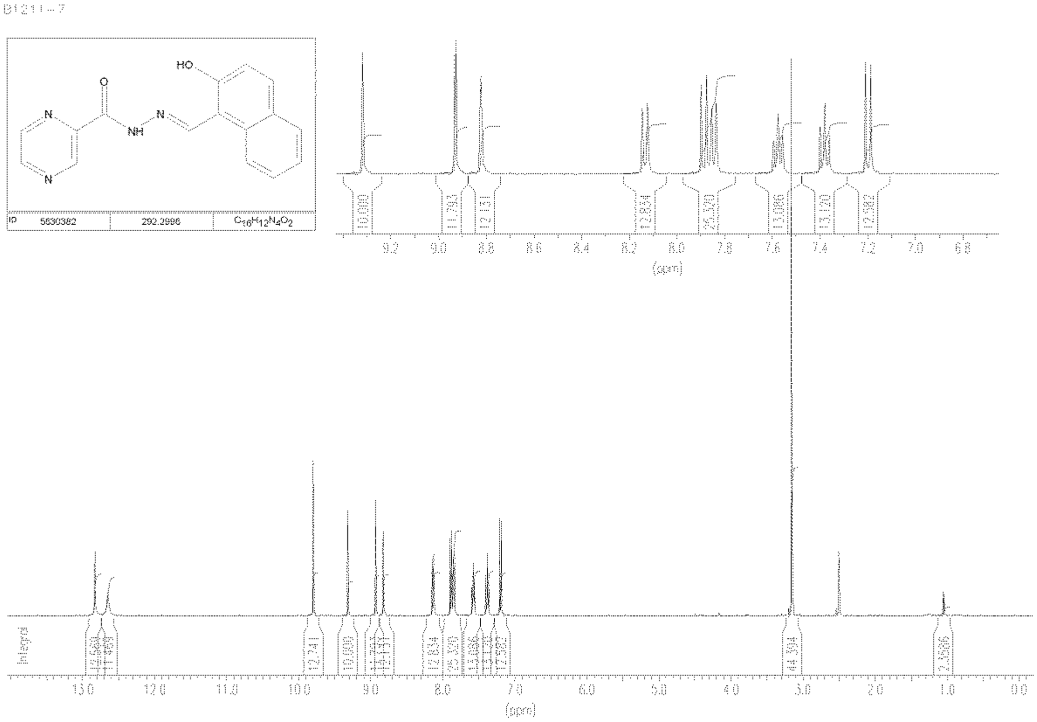

Sample ID: SAR9 (C16 H12 N4 O2) 292.29 Da (in MeOH)

WANG-TNGTING-082214-SAR9 13 (0.143) AM (Cen,2, 80.00, Ht,6000.0,0.00,0.70); Sm (Mn, 2x3.00); Sb (1,40.00); Cm (1:95)

TOF MS ES+
560

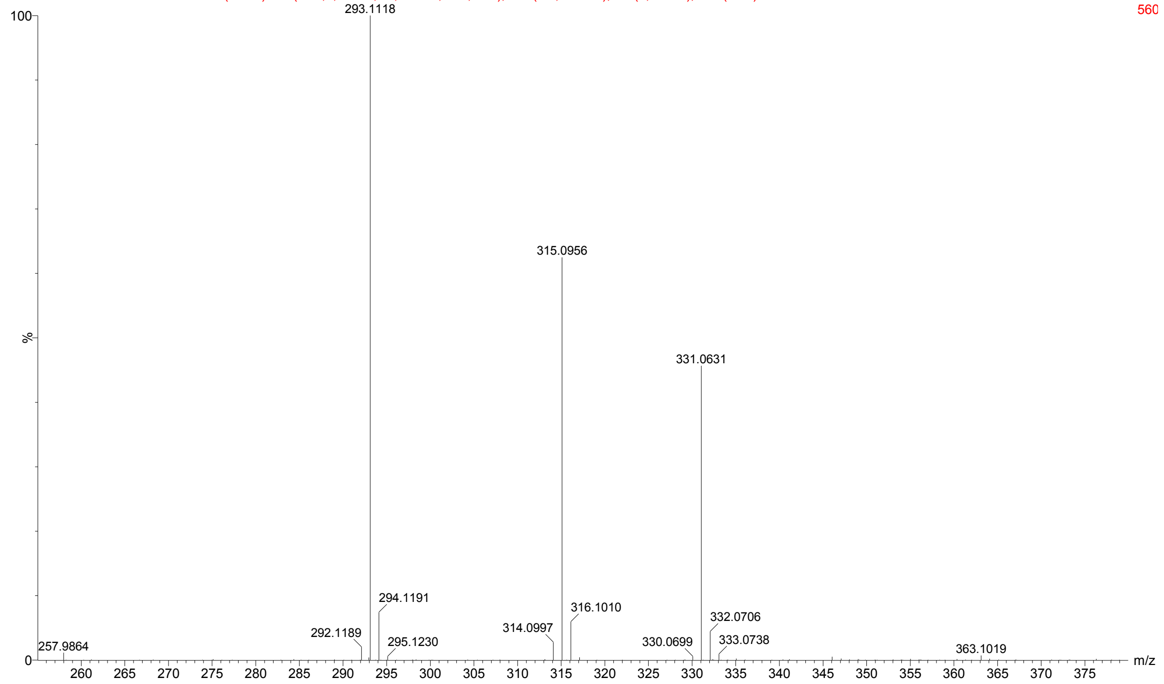

SAR-12

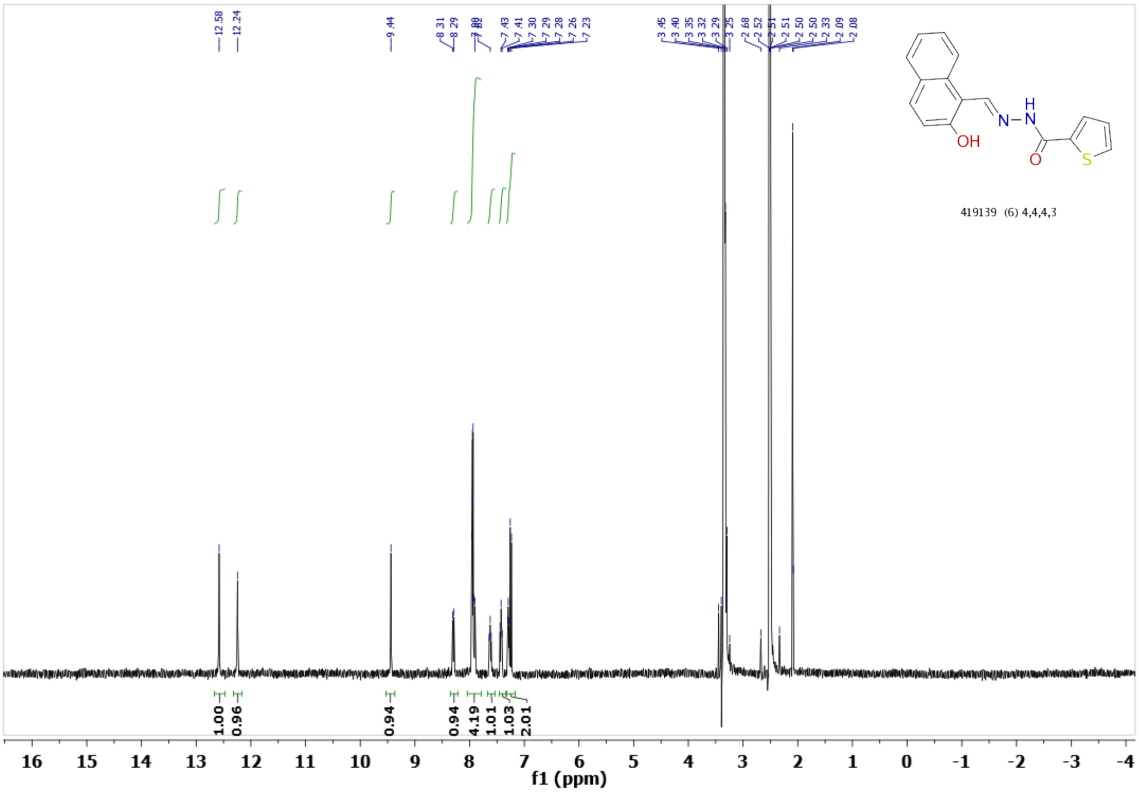

Sample ID: 419139 (C16 H12 N2 O2 S) 296.341 Da (in MeOH/ESIB)

WANG-TINGTING-090214-419139-R2 60 (0.627) AM (Cen,2, 80.00, Ht,6000.0,0.00,1.00); Sm (Mn, 2x3.00); Sb (1,40.00); Cm (1:96)

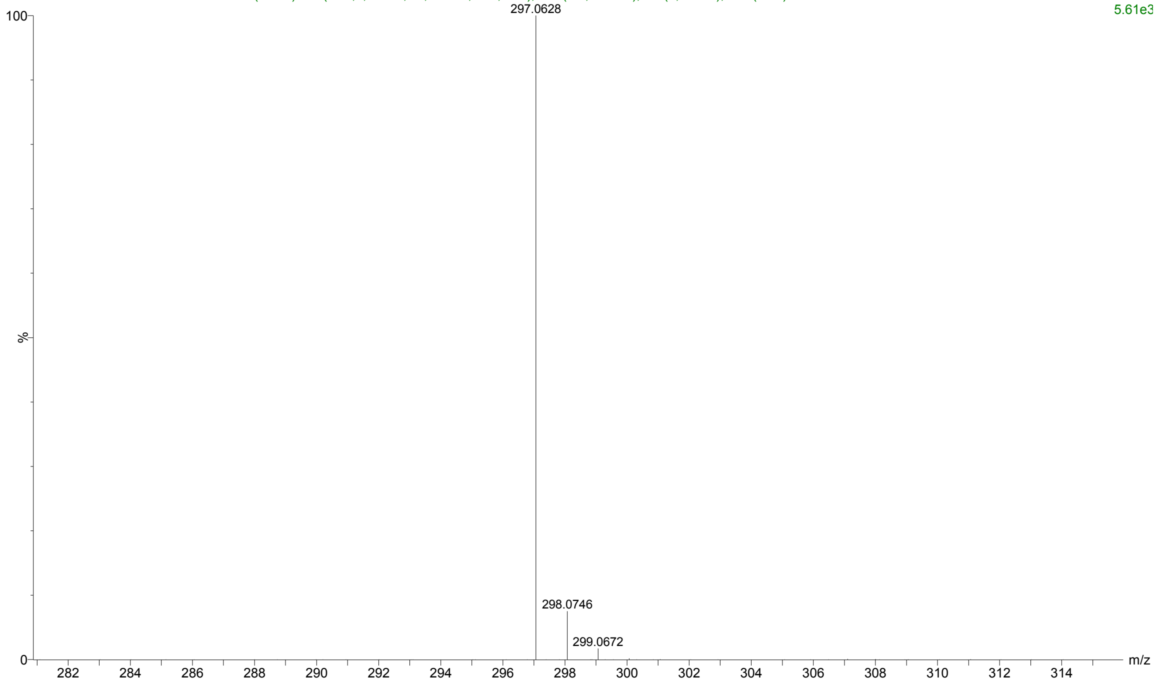

SAR-13

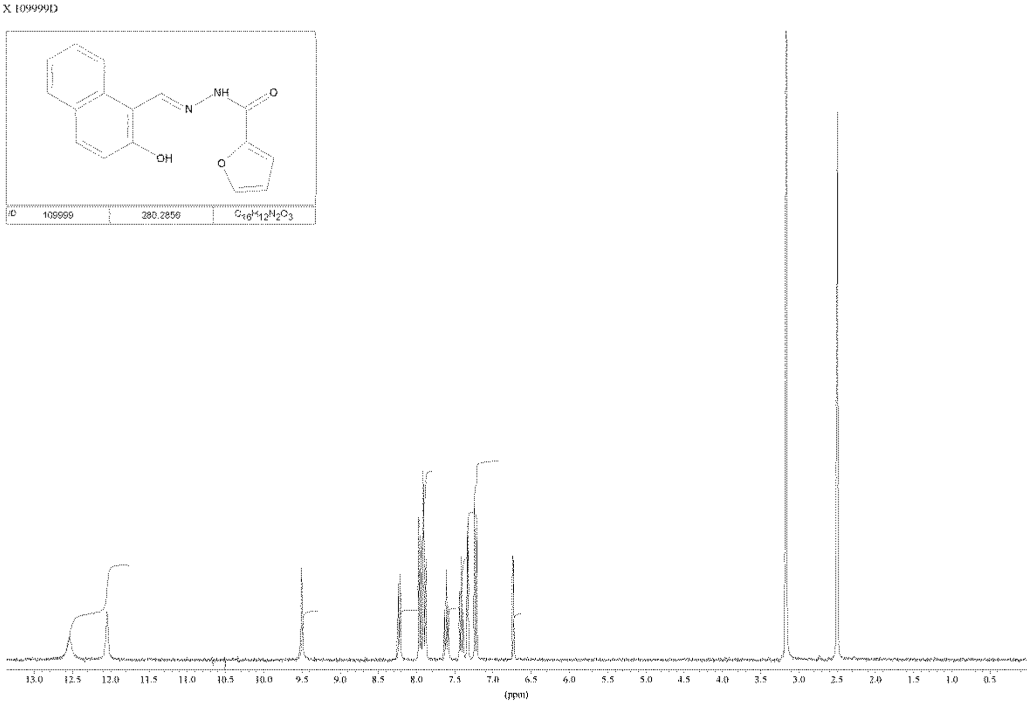
X 109999D
NH
O
N
OH
O
ID 109999
280.2856
$C_{16}H_{12}N_2O_3$
13.0 12.5 12.0 11.5 11.0 10.5 10.0 9.5 9.0 8.5 8.0 7.5 7.0 6.5 6.0 5.5 5.0 4.5 4.0 3.5 3.0 2.5 2.0 1.5 1.0 0.5
(ppm)

SAR-14

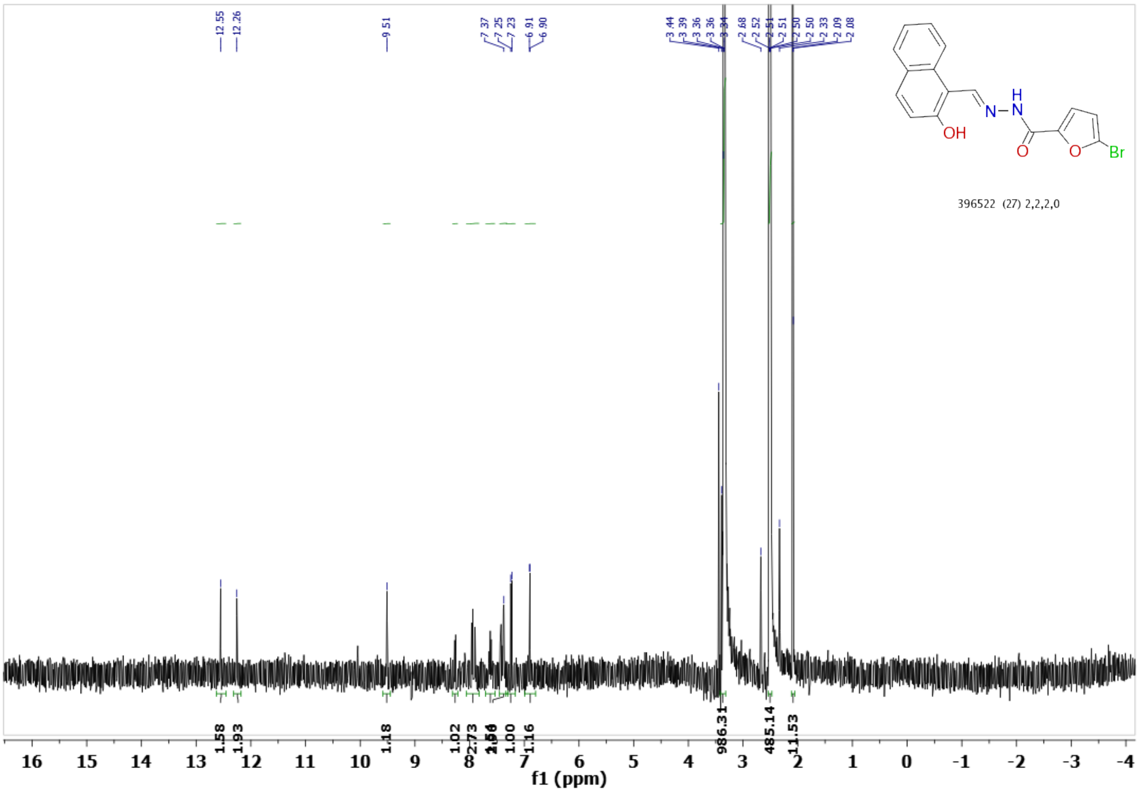

Sample ID: 396522 (C16 H11 Br N2 O3) 359.17 Da (in MeOH/ESIB)

WANG-TINGTING-090214-396522-R2 77 (0.802) AM (Cen,2, 80.00, Ht,6000.0,0.00,1.00); Sm (Mn, 2x3.00); Sb (1,40.00); Cm (1:97)

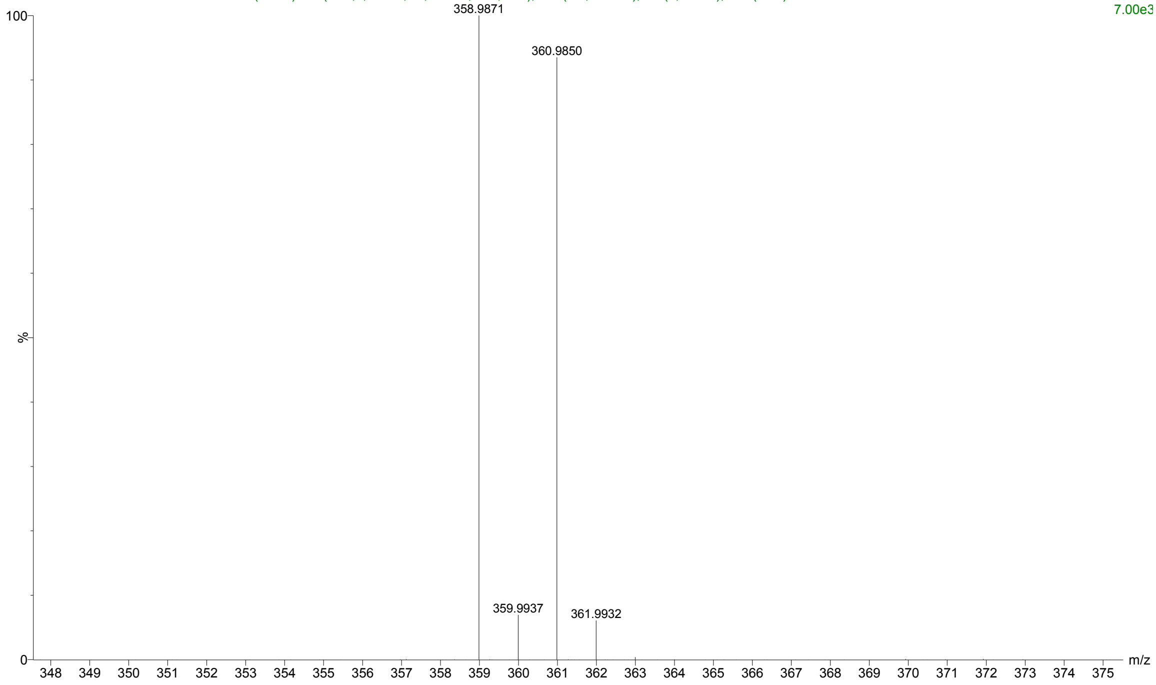

SAR-15

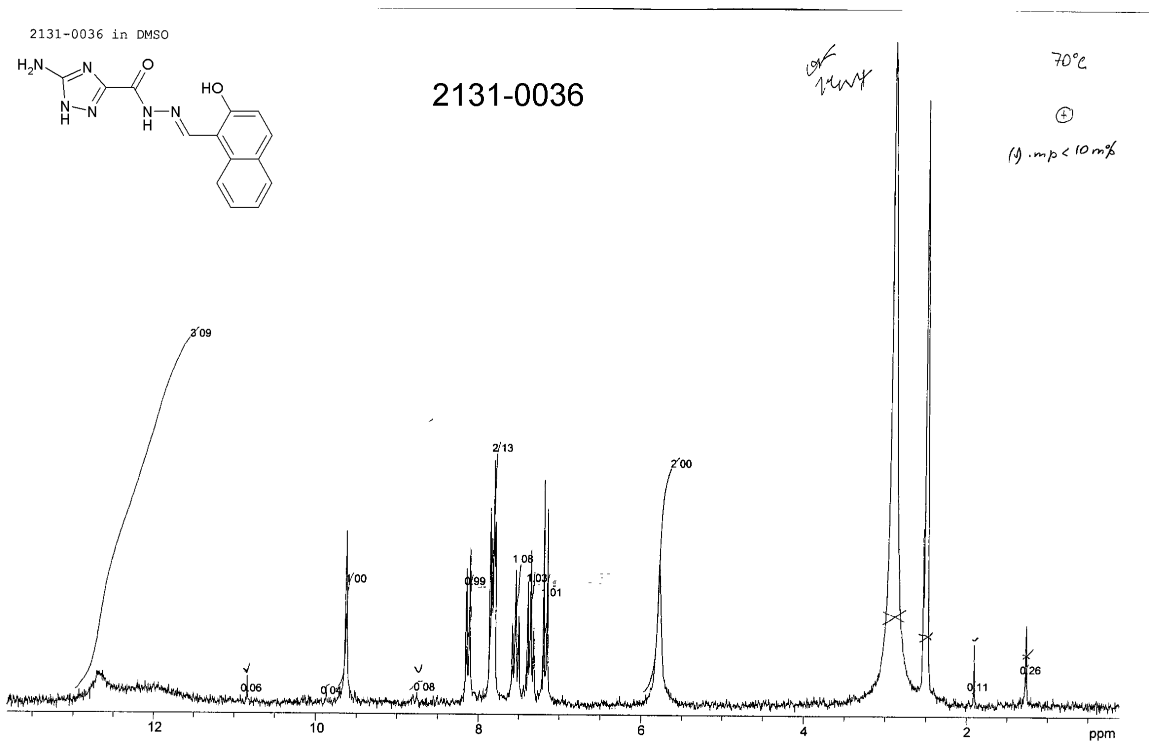
2131-0036 in DMSO
2131-0036
70°C
ppm

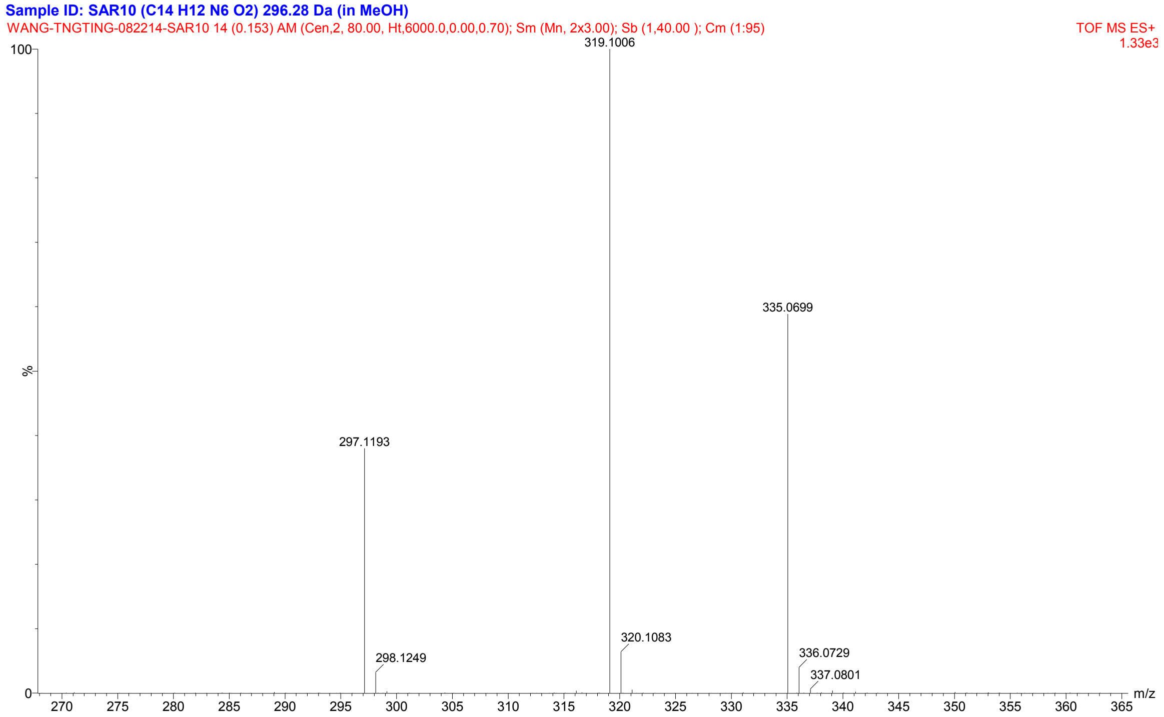
Sample ID: SAR10 (C14 H12 N6 O2) 296.28 Da (in MeOH)
WANG-TNGTING-082214-SAR10 14 (0.153) AM (Cen,2, 80.00, Ht,6000.0,0.00,0.70); Sm (Mn, 2x3.00); Sb (1,40.00); Cm (1:95)
TOF MS ES+
1.33e3
319.1006
335.0699
297.1193
320.1083
336.0729
298.1249
337.0801
m/z
%

SAR-16

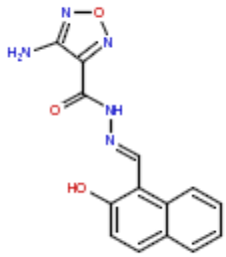

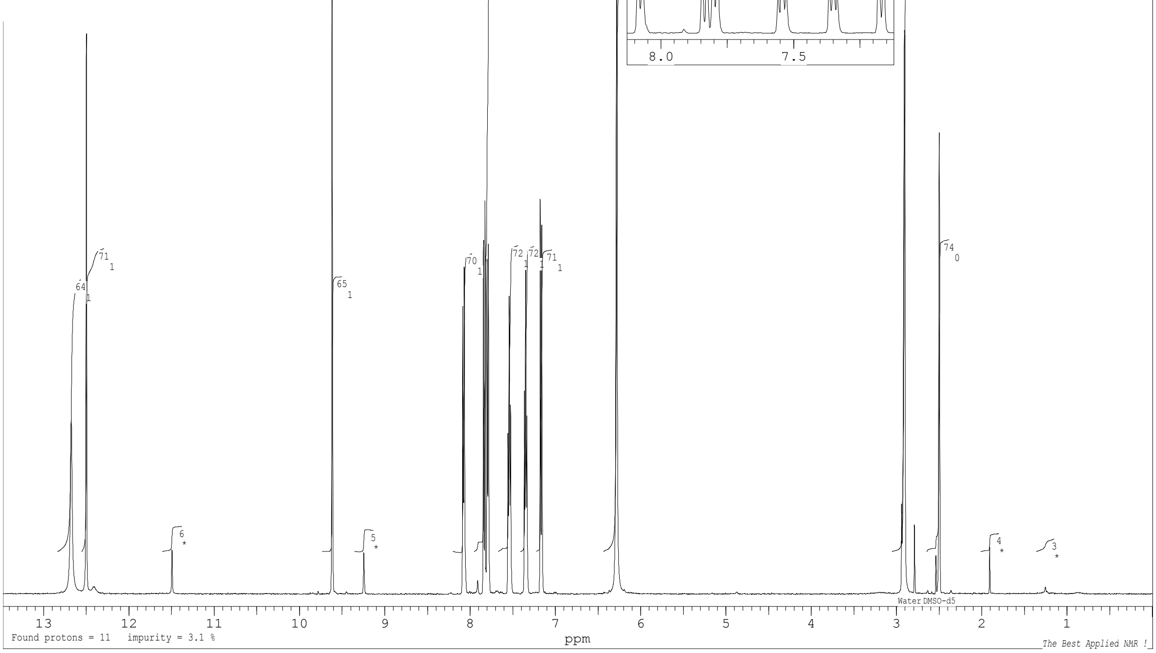

Sample ID: SAR11 (C14 H12 N5 O3) 298.28 Da (in MeOH)

WANG-TNGTING-082214-SAR11 39 (0.413) AM (Cen,2, 80.00, Ht,6000.0,0.00,0.70); Sm (Mn, 2x3.00); Sb (1,40.00); Cm (1:95)

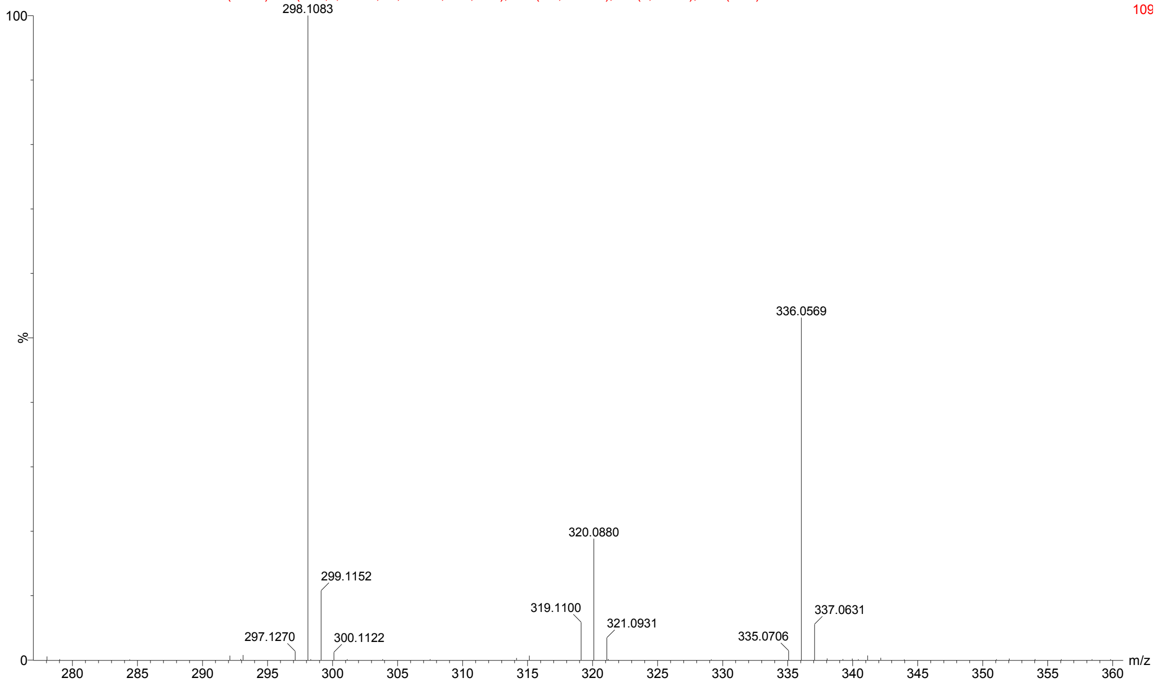

SAR-17

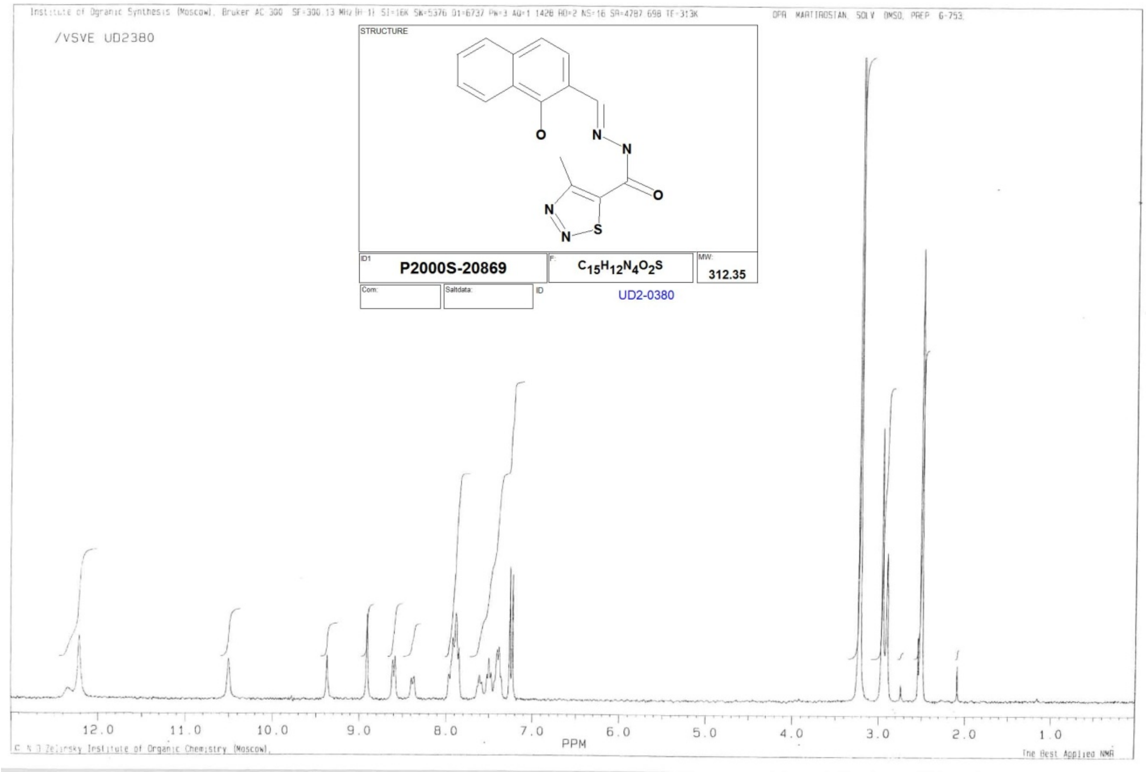

Institute of Organic Synthesis (Moscow). Bruker AC 300 SF=300.13 MHz (H-1) SI=16K SW=5376 O1=6737 PW=3 AQ=1.1428 RD=2 NS=16 SR=4787.698 TE=313K
OPR MARTIROSIAN SOLV DMSO PREP G-753
/VSVE UD2380
STRUCTURE
P2000S-20869
$C_{15}H_{12}N_4O_2S$
MW: 312.35
UD2-0380
PPM
N.D. Zelinsky Institute of Organic Chemistry (Moscow)

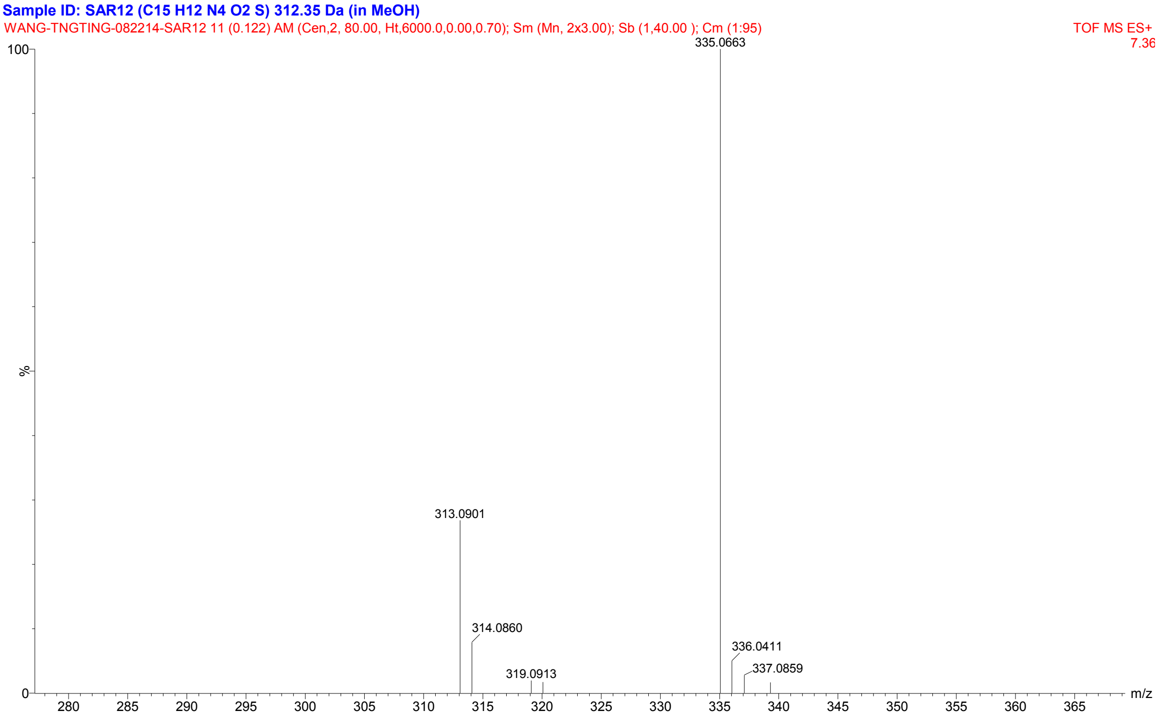

Sample ID: SAR12 (C15 H12 N4 O2 S) 312.35 Da (in MeOH)
WANG-TNGTING-082214-SAR12 11 (0.122) AM (Cen,2, 80.00, Ht,6000.0,0.00,0.70); Sm (Mn, 2x3.00); Sb (1,40.00); Cm (1:95)
TOF MS ES+
7.36
335.0663
313.0901
314.0860
319.0913
336.0411
337.0859
%
m/z

SAR-18

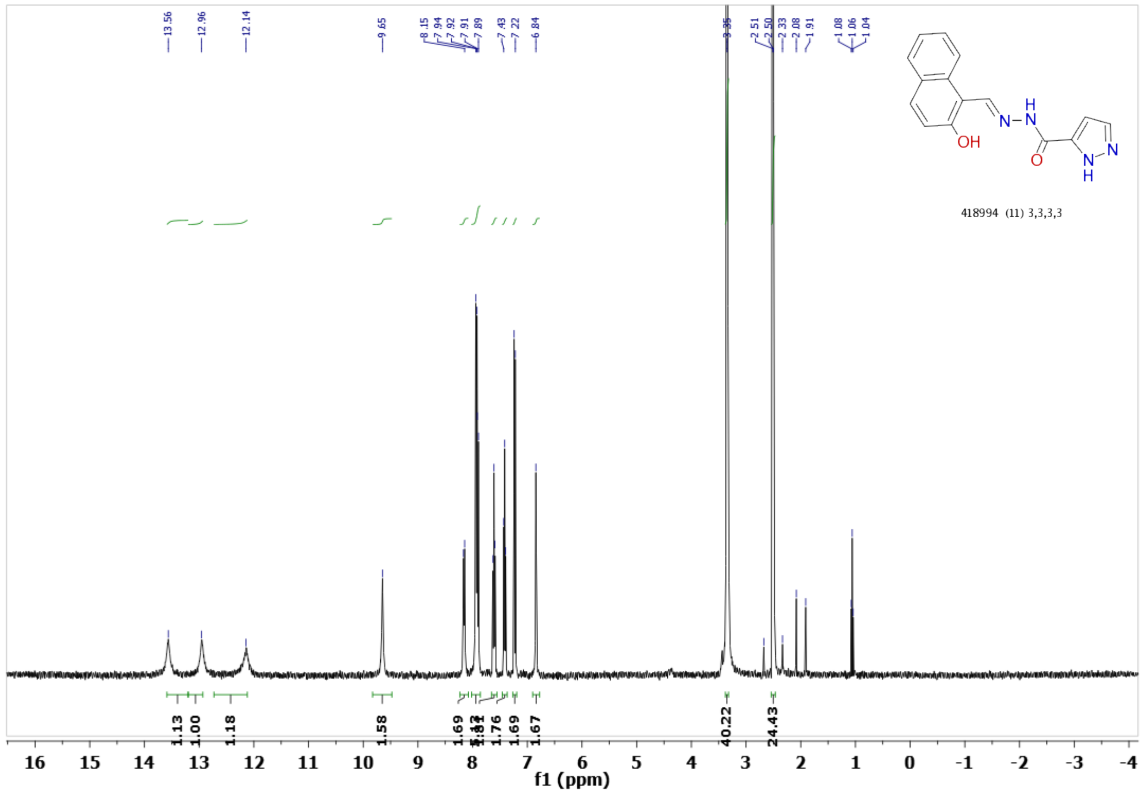

418994 (11) 3,3,3,3
f1 (ppm)
13.56
12.96
12.14
9.65
8.15
7.94
7.92
7.91
7.89
7.43
7.22
6.84
3.35
2.51
2.50
2.33
2.08
1.91
1.08
1.06
1.04
1.13
1.00
1.18
1.58
1.69
1.76
1.69
1.67
40.22
24.43
OH
O

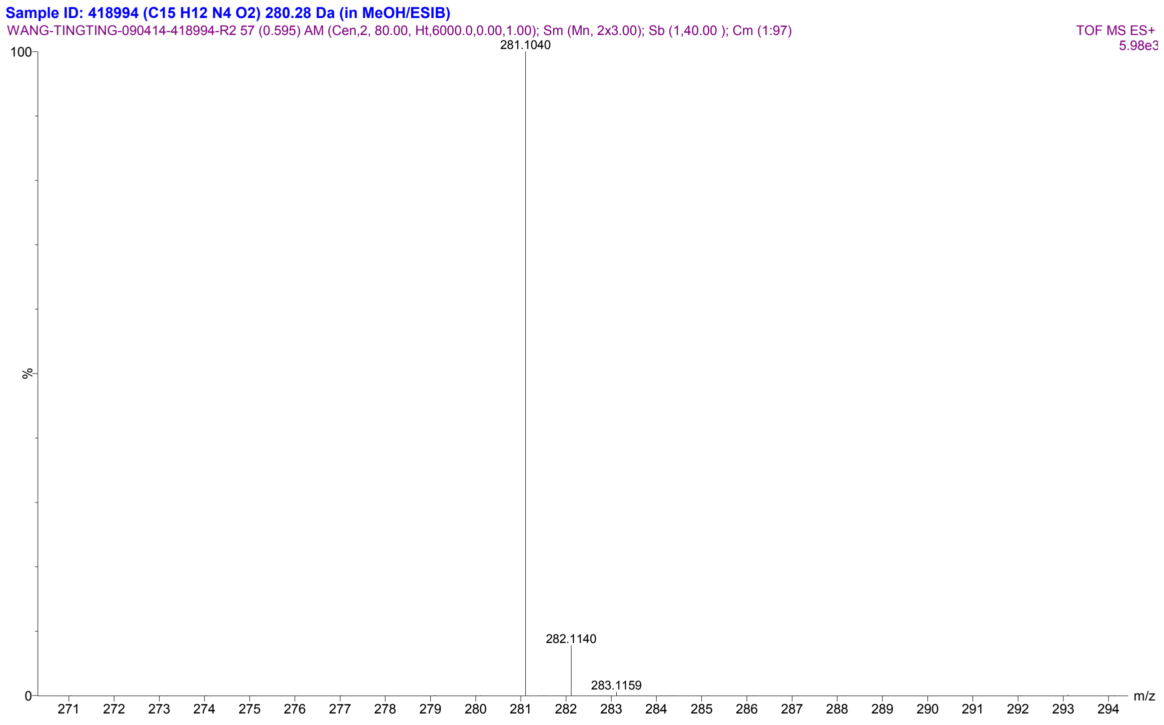

Sample ID: 418994 (C15 H12 N4 O2) 280.28 Da (in MeOH/ESIB)
WANG-TINGTING-090414-418994-R2 57 (0.595) AM (Cen,2, 80.00, Ht,6000.0,0.00,1.00); Sm (Mn, 2x3.00); Sb (1,40.00); Cm (1:97)
TOF MS ES+
5.98e3
281.1040
282.1140
283.1159
%
m/z

SAR-19

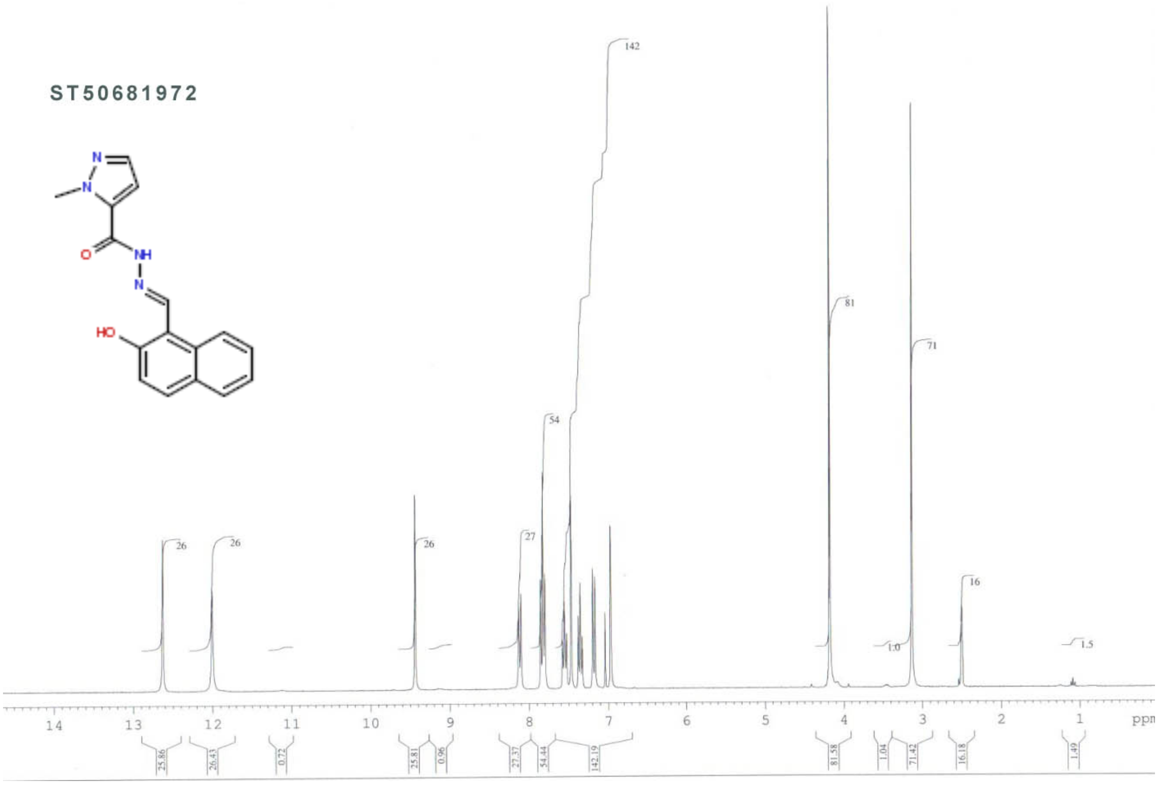

Sample ID: SAR13 (C16 H14 N4 O2) 294.31 Da (in MeOH)

WANG-TNGTING-082214-SAR13 32 (0.340) AM (Cen,2, 80.00, Ht,6000.0,0.00,0.70); Sm (Mn, 2x3.00); Sb (1,40.00); Cm (1:95)

TOF MS ES+
416

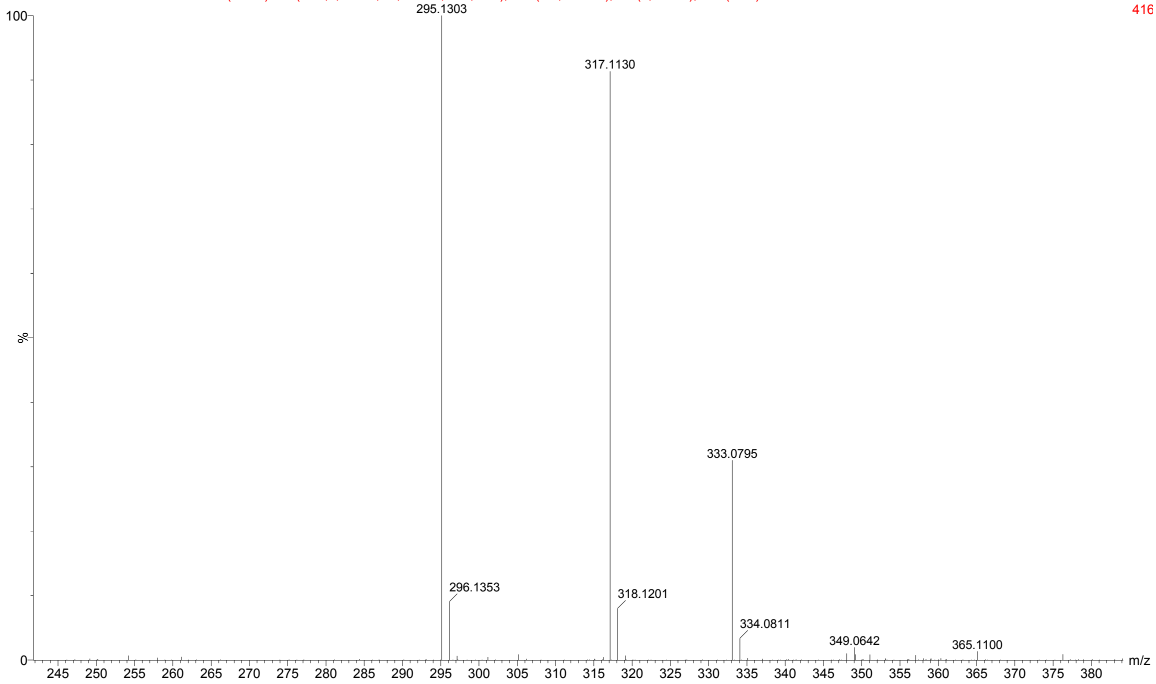

SAR-20

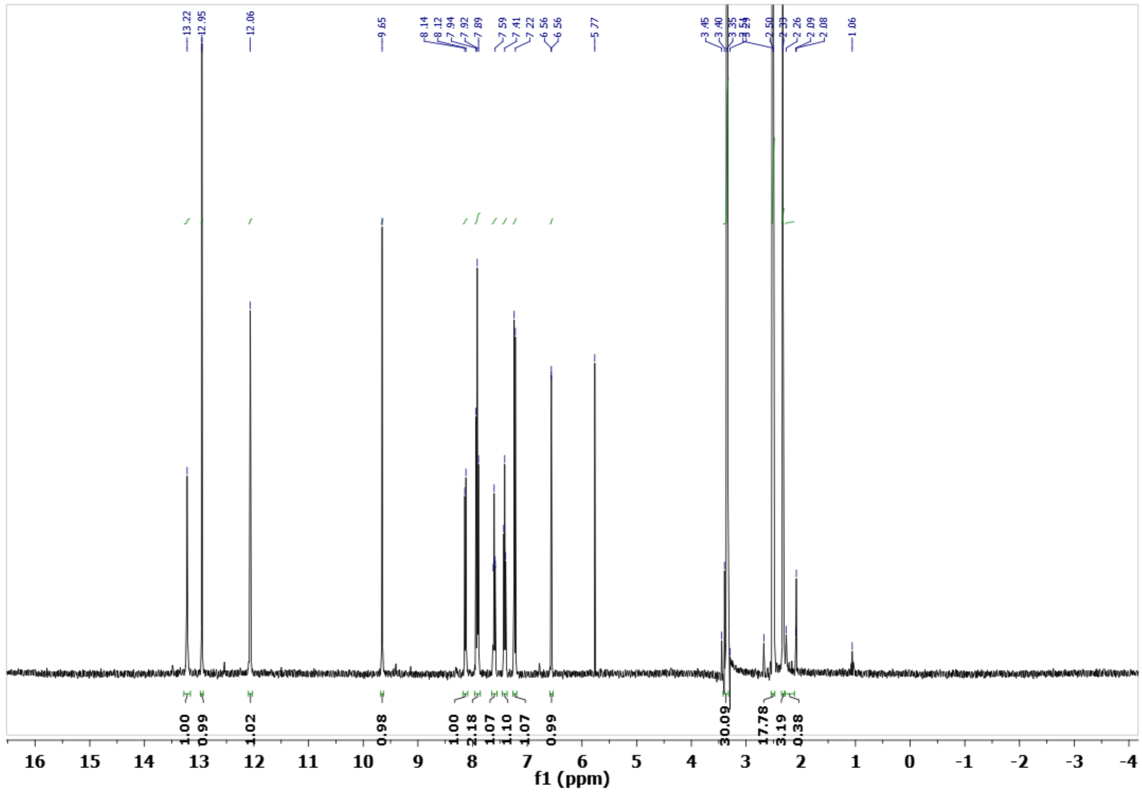

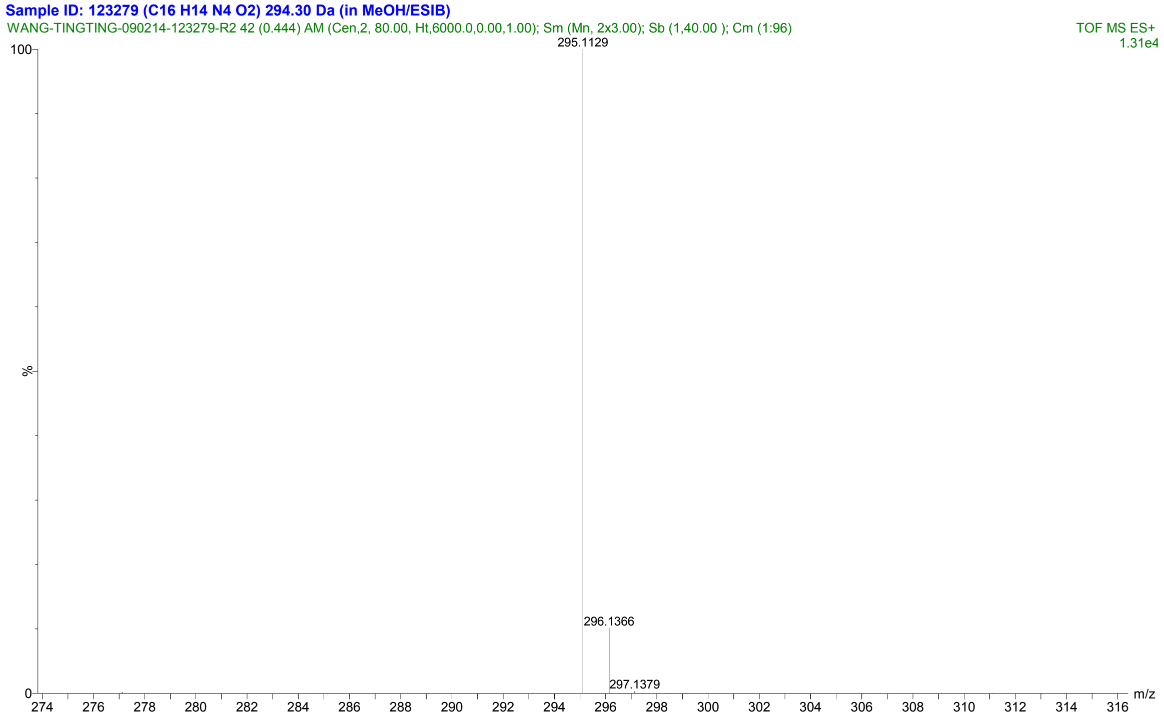
Sample ID: 123279 (C16 H14 N4 O2) 294.30 Da (in MeOH/ESIB)
WANG-TINGTING-090214-123279-R2 42 (0.444) AM (Cen,2, 80.00, Ht,6000.0,0.00,1.00); Sm (Mn, 2x3.00); Sb (1,40.00); Cm (1:96)
TOF MS ES+
1.31e4
295.1129
296.1366
297.1379
%
m/z

SAR-22

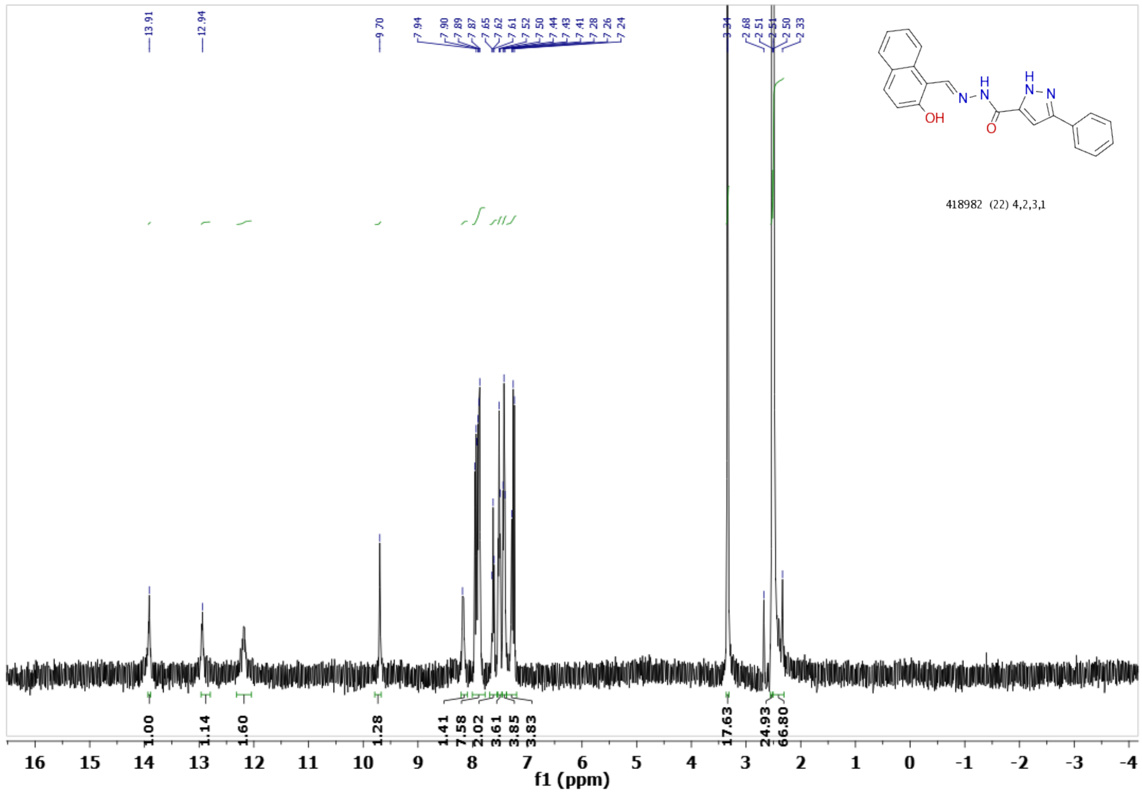

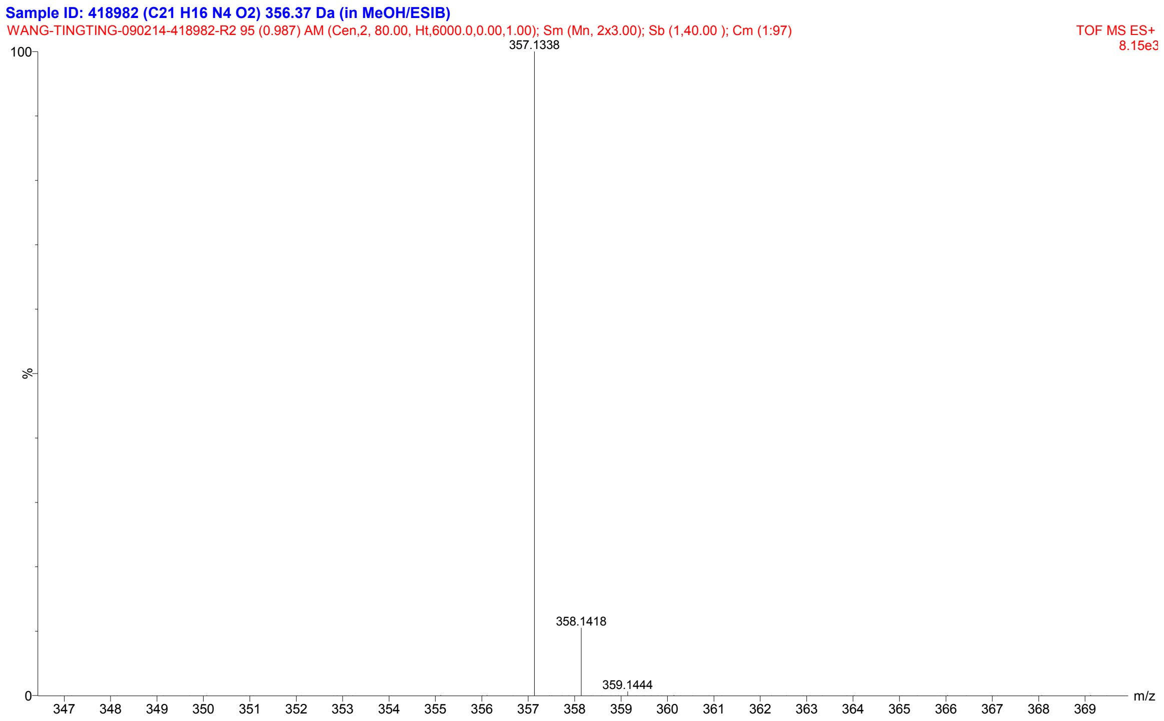
Sample ID: 418982 (C21 H16 N4 O2) 356.37 Da (in MeOH/ESIB)
WANG-TINGTING-090214-418982-R2 95 (0.987) AM (Cen,2, 80.00, Ht,6000.0,0.00,1.00); Sm (Mn, 2x3.00); Sb (1,40.00); Cm (1:97)
TOF MS ES+
8.15e3
357.1338
358.1418
359.1444
m/z

SAR-23

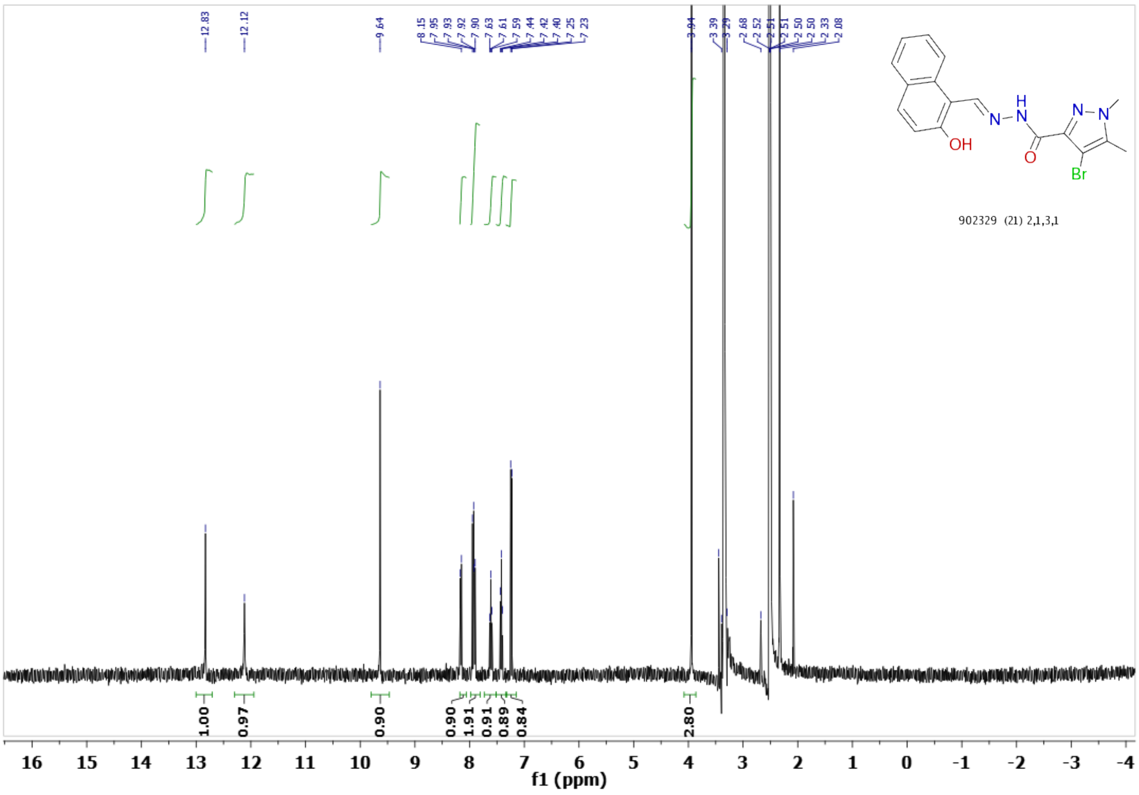

Sample ID: 902329 (C17 H16 Br N4 O2) 387.23 Da (in MeOH/NaI)

WANG-TINGTING-082914-902329-NAI 95 (0.994) AM (Cen,2, 80.00, Ht,6000.0,0.00,1.00); Sm (Mn, 2x3.00); Sb (1,40.00); Cm (1:96)

TOF MS ES+
3.40e4

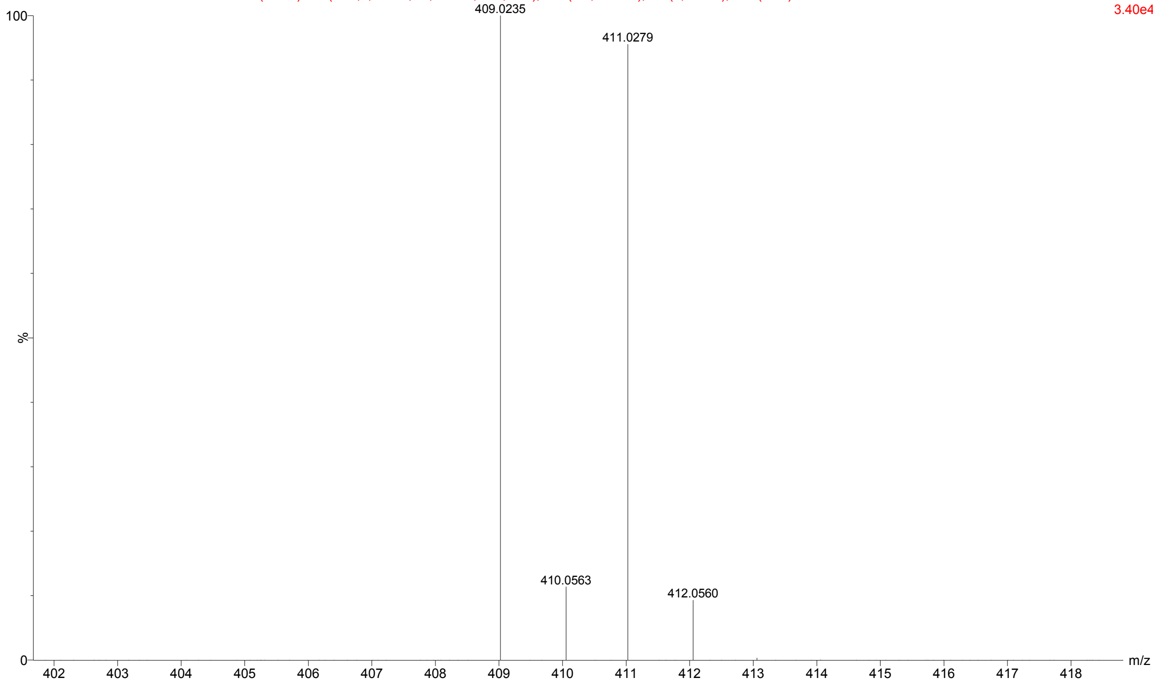

SAR-24

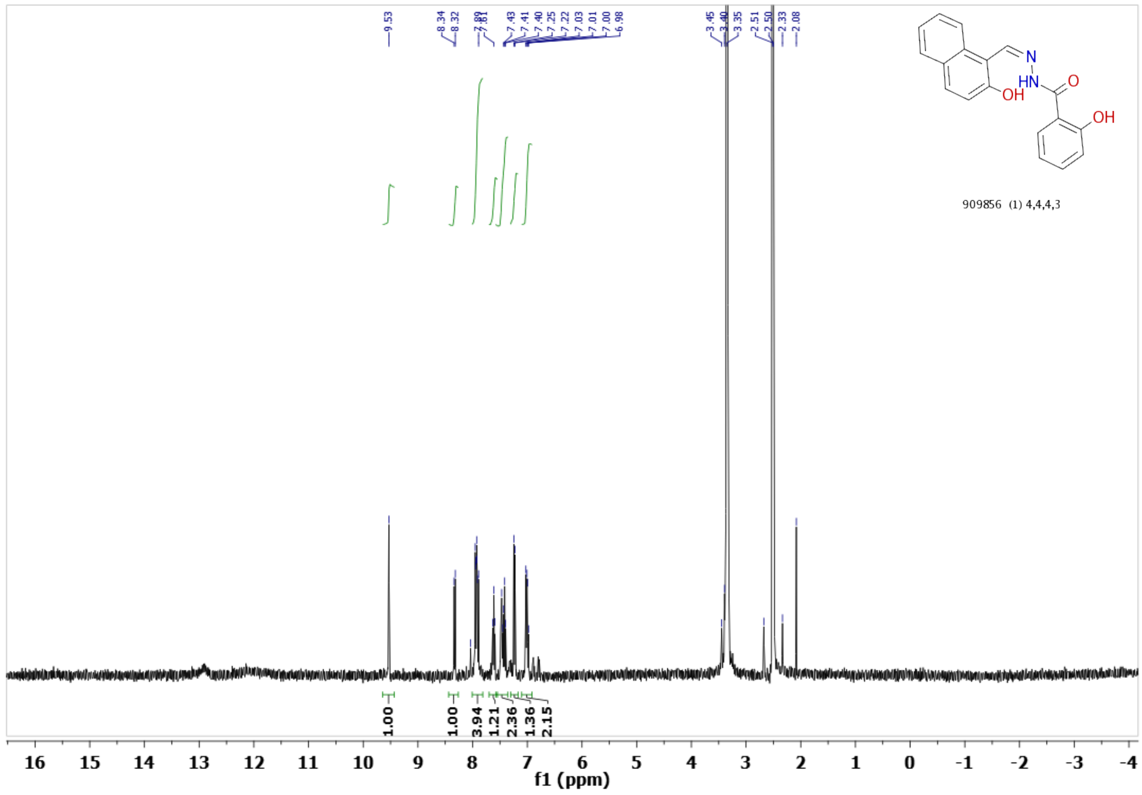

9.53
8.34
8.32
7.89
7.81
7.43
7.41
7.40
7.25
7.22
7.03
7.01
7.00
6.98
3.45
3.40
3.35
2.51
2.50
2.33
2.08
N
HN
O
OH
OH
909856 (1) 4,4,4,3
1.00
1.00
3.94
1.21
2.36
1.36
2.15
f1 (ppm)

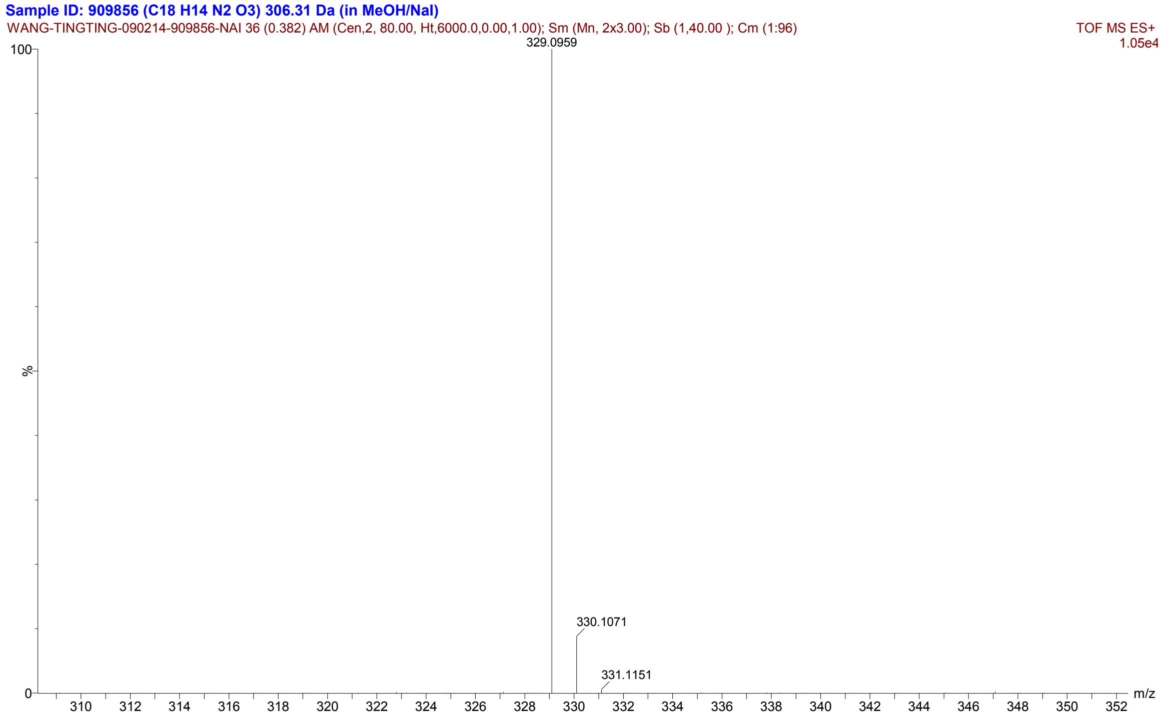

Sample ID: 909856 (C18 H14 N2 O3) 306.31 Da (in MeOH/NaI)
WANG-TINGTING-090214-909856-NAI 36 (0.382) AM (Cen,2, 80.00, Ht,6000.0,0.00,1.00); Sm (Mn, 2x3.00); Sb (1,40.00); Cm (1:96)
TOF MS ES+
1.05e4
329.0959
330.1071
331.1151
%
m/z

SAR-25

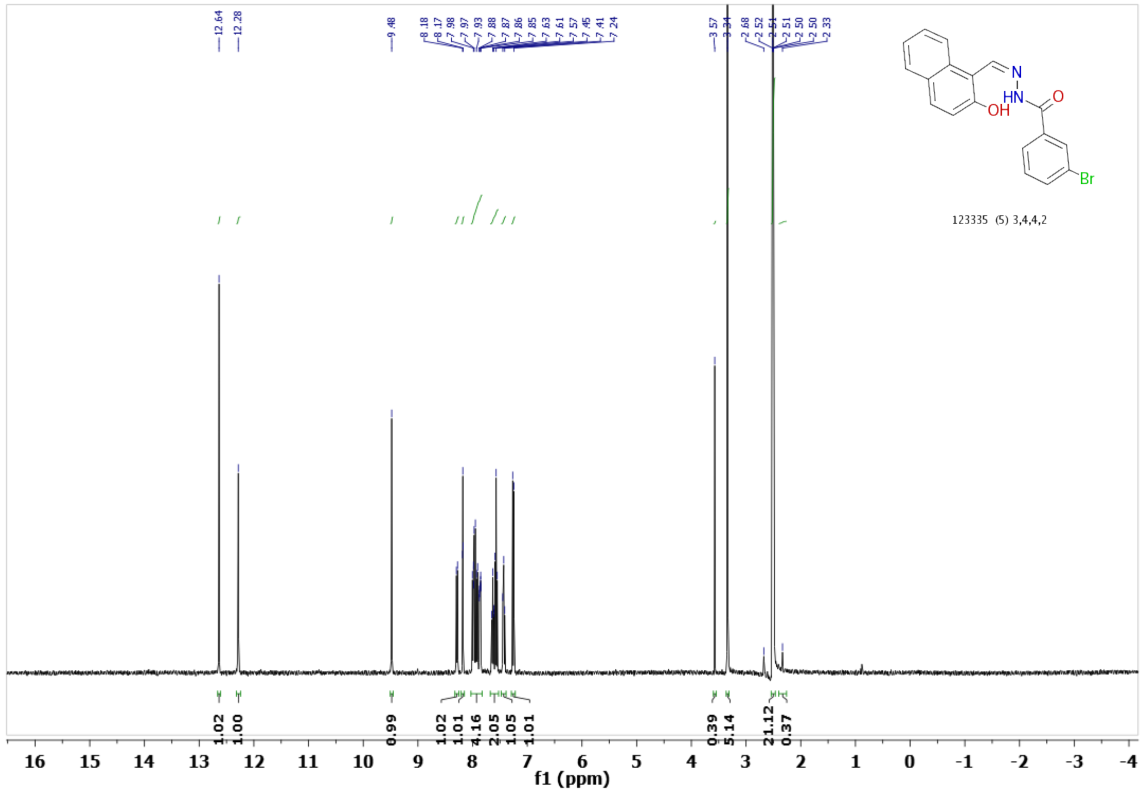
12.64
12.28
9.48
8.18 8.17 7.98 7.97 7.93 7.88 7.87 7.86 7.85 7.63 7.61 7.57 7.45 7.41 7.24
3.57 3.34 2.68 2.52 2.51 2.51 2.50 2.50 2.33
N
HN
O
OH
Br
123335 (5) 3,4,4,2
1.02 1.00 0.99 1.02 1.01 4.16 2.05 1.05 1.01 0.39 5.14 21.12 0.37
16 15 14 13 12 11 10 9 8 7 6 5 4 3 2 1 0 -1 -2 -3 -4
f1 (ppm)

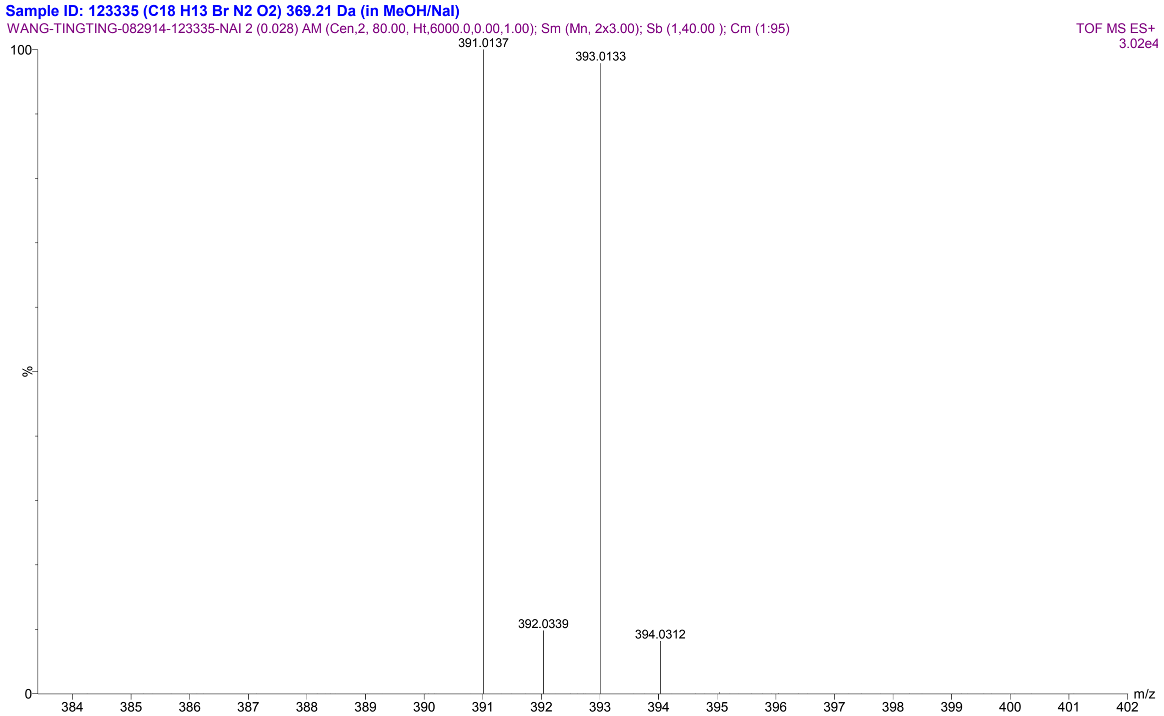
Sample ID: 123335 (C18 H13 Br N2 O2) 369.21 Da (in MeOH/NaI)
WANG-TINGTING-082914-123335-NAI 2 (0.028) AM (Cen,2, 80.00, Ht,6000.0,0.00,1.00); Sm (Mn, 2x3.00); Sb (1,40.00); Cm (1:95)
TOF MS ES+
3.02e4
100
%
0
391.0137
393.0133
392.0339
394.0312
384 385 386 387 388 389 390 391 392 393 394 395 396 397 398 399 400 401 402
m/z

SAR-26

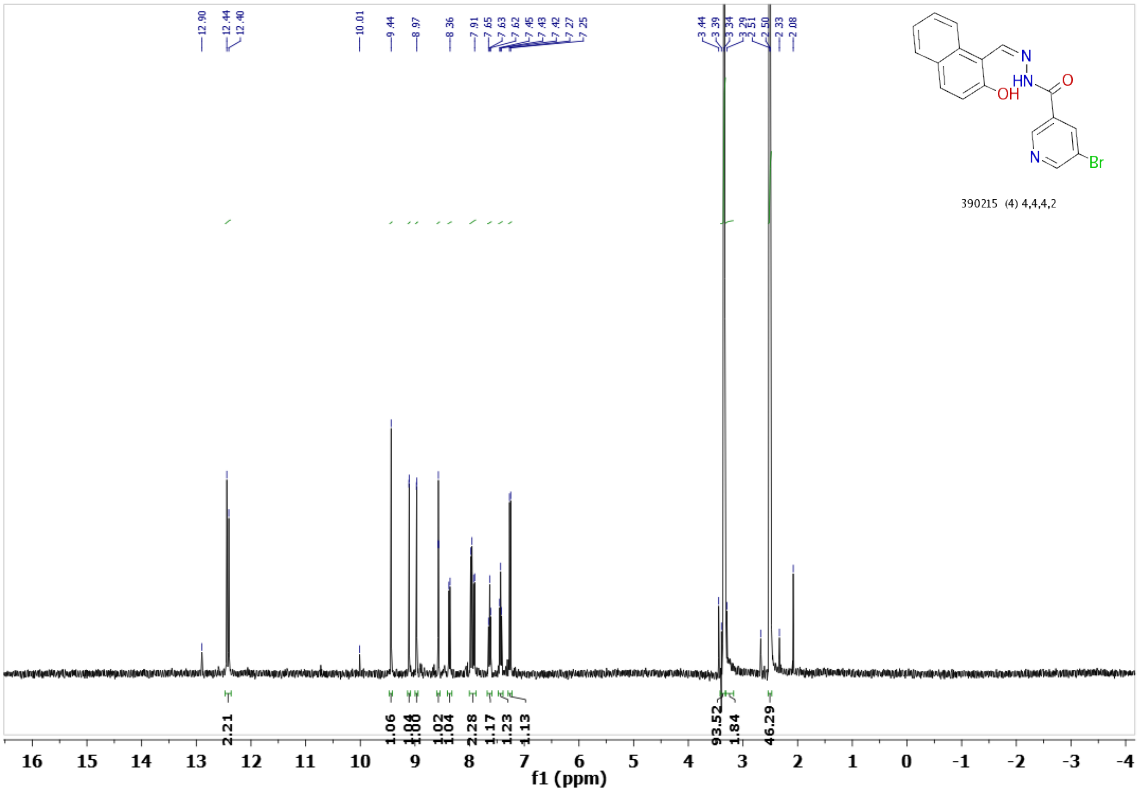

390215 (4) 4,4,4,2
f1 (ppm)

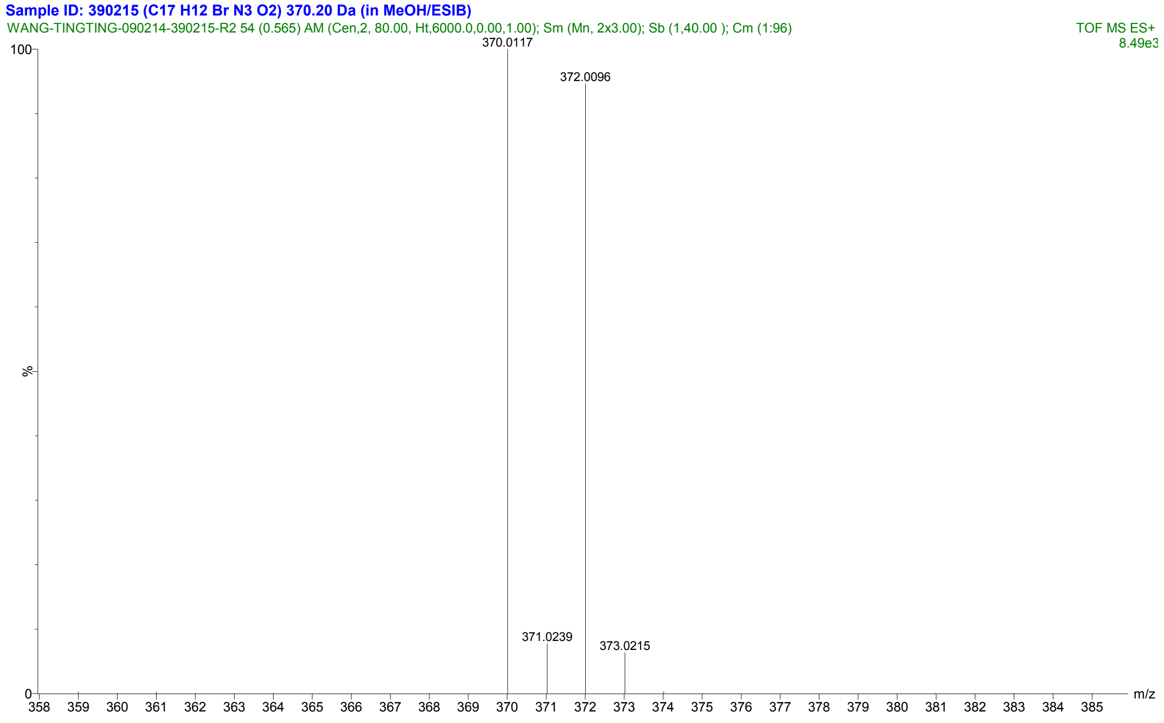

Sample ID: 390215 (C17 H12 Br N3 O2) 370.20 Da (in MeOH/ESIB)
WANG-TINGTING-090214-390215-R2 54 (0.565) AM (Cen,2, 80.00, Ht,6000.0,0.00,1.00); Sm (Mn, 2x3.00); Sb (1,40.00); Cm (1:96)
TOF MS ES+
8.49e3
370.0117
372.0096
371.0239
373.0215
%
m/z

SAR-28

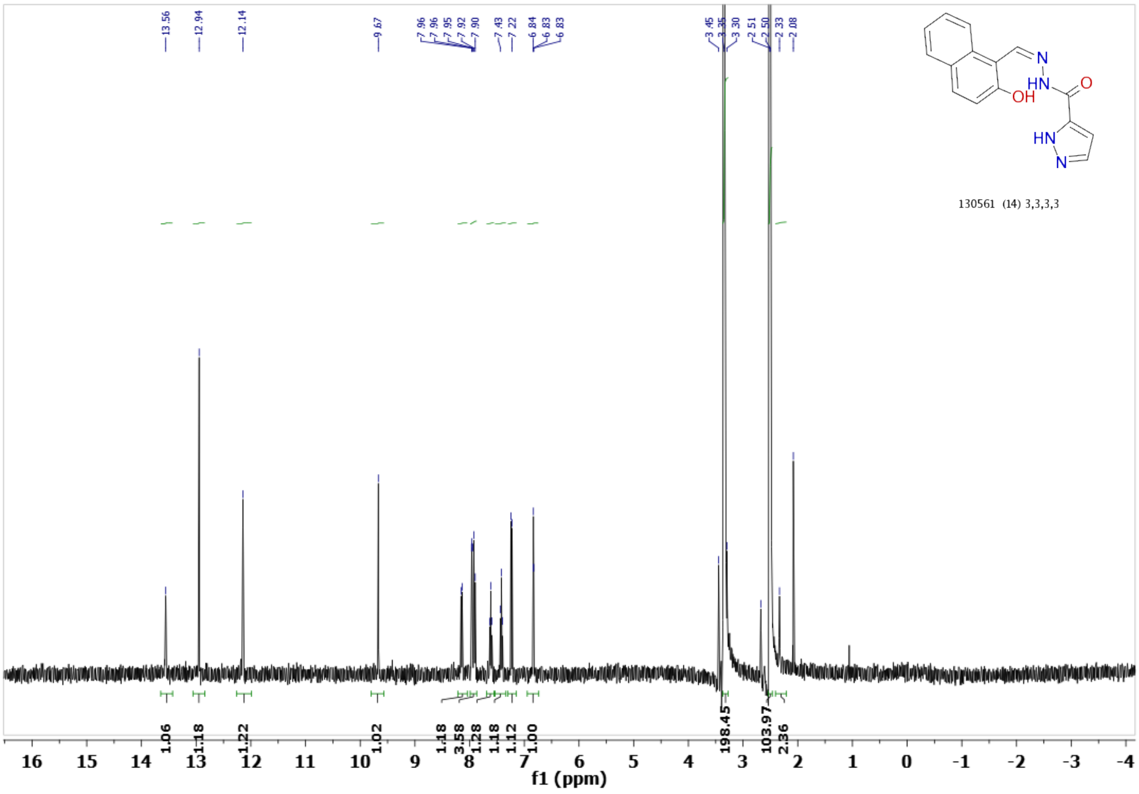

13.56
12.94
12.14
9.67
7.96
7.96
7.95
7.92
7.90
7.43
7.22
6.84
6.83
6.83
3.45
3.35
3.30
2.51
2.50
2.33
2.08
N
HN
O
OH
HN
N
130561 (14) 3,3,3,3
1.06
1.18
1.22
1.02
1.18
3.58
1.28
1.18
1.12
1.00
198.45
103.97
2.36
16 15 14 13 12 11 10 9 8 7 6 5 4 3 2 1 0 -1 -2 -3 -4
f1 (ppm)

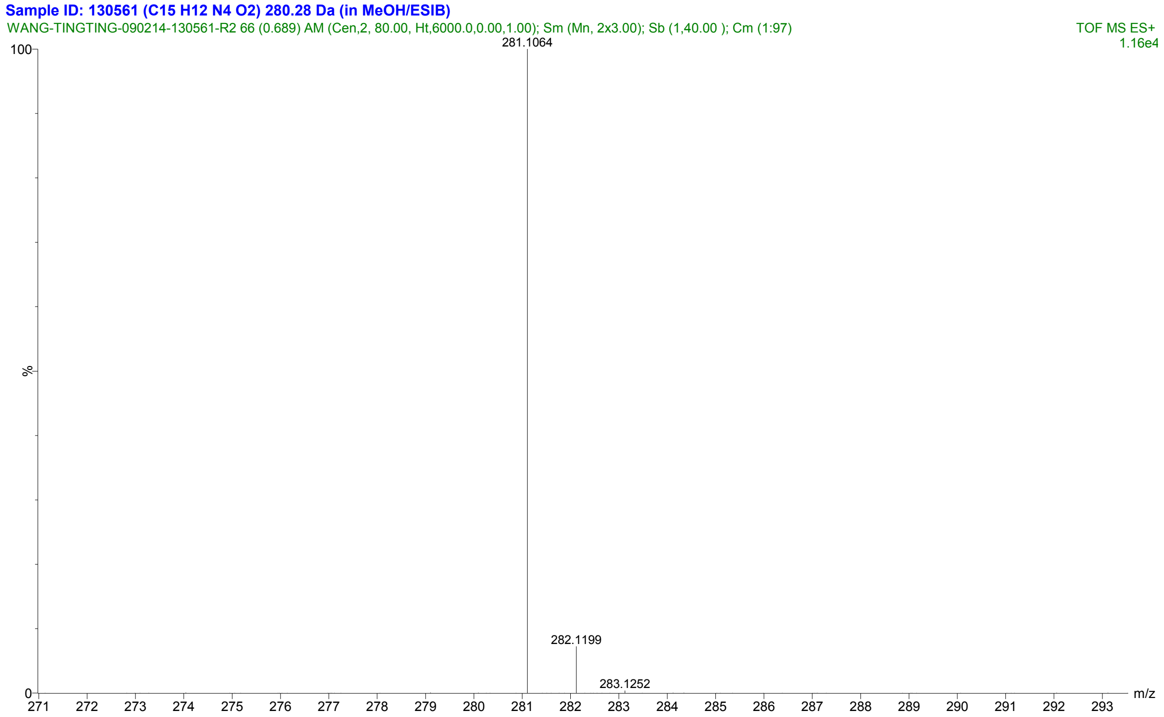

Sample ID: 130561 (C15 H12 N4 O2) 280.28 Da (in MeOH/ESIB)
WANG-TINGTING-090214-130561-R2 66 (0.689) AM (Cen,2, 80.00, Ht,6000.0,0.00,1.00); Sm (Mn, 2x3.00); Sb (1,40.00); Cm (1:97)
TOF MS ES+
1.16e4
281.1064
100
%
0
282.1199
283.1252
271 272 273 274 275 276 277 278 279 280 281 282 283 284 285 286 287 288 289 290 291 292 293
m/z

SAR-30

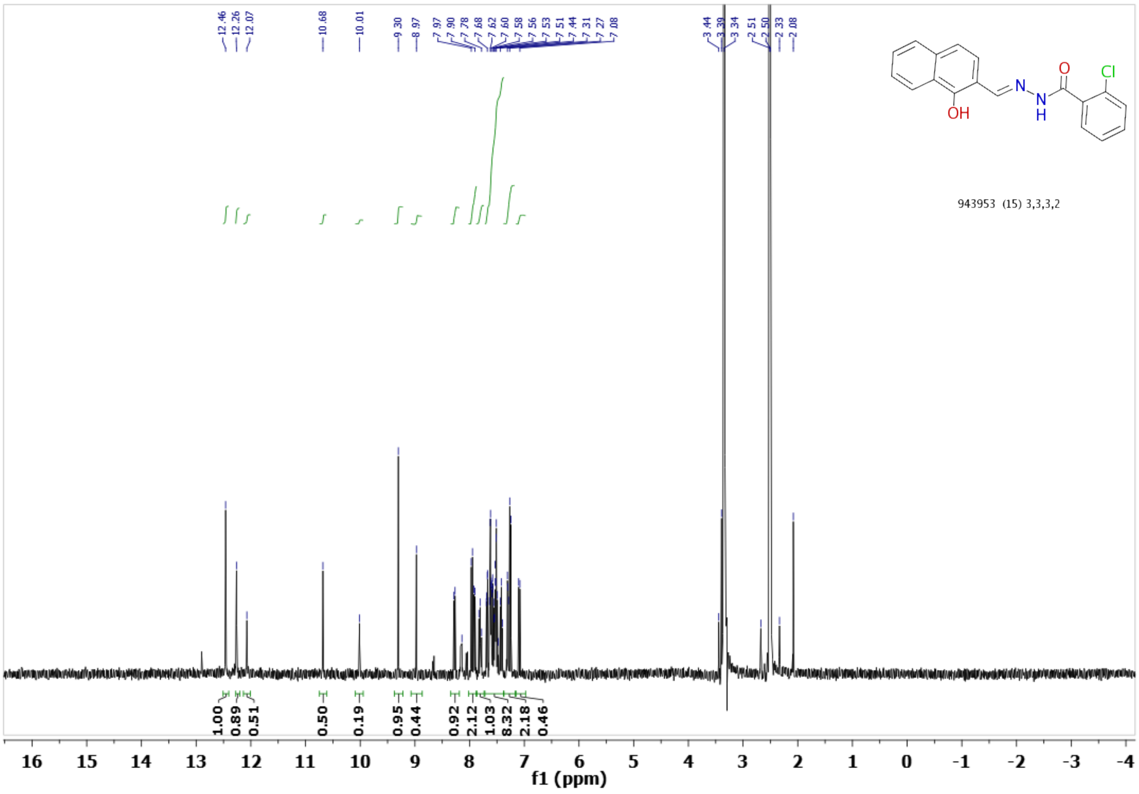

12.46
12.26
12.07
10.68
10.01
9.30
8.97
7.97
7.90
7.78
7.68
7.62
7.60
7.58
7.56
7.53
7.51
7.44
7.31
7.27
7.08
3.44
3.39
3.34
2.51
2.50
2.33
2.08
OH
N
N
H
O
Cl
943953 (15) 3,3,3,2
1.00
0.89
0.51
0.50
0.19
0.95
0.44
0.92
2.12
1.03
8.32
2.18
0.46
16 15 14 13 12 11 10 9 8 7 6 5 4 3 2 1 0 -1 -2 -3 -4
f1 (ppm)

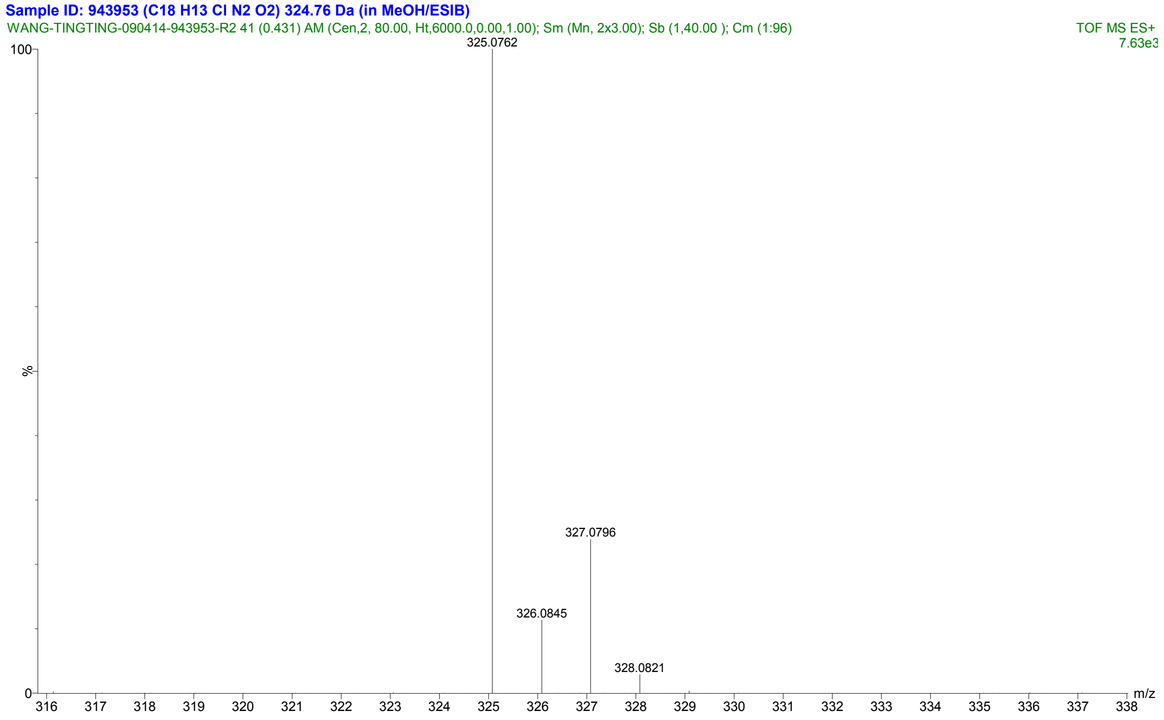

Sample ID: 943953 (C18 H13 Cl N2 O2) 324.76 Da (in MeOH/ESIB)
WANG-TINGTING-090414-943953-R2 41 (0.431) AM (Cen,2, 80.00, Ht,6000.0,0.00,1.00); Sm (Mn, 2x3.00); Sb (1,40.00); Cm (1:96)
TOF MS ES+
7.63e3
100
%
0
325.0762
327.0796
326.0845
328.0821
316 317 318 319 320 321 322 323 324 325 326 327 328 329 330 331 332 333 334 335 336 337 338
m/z

SAR-31

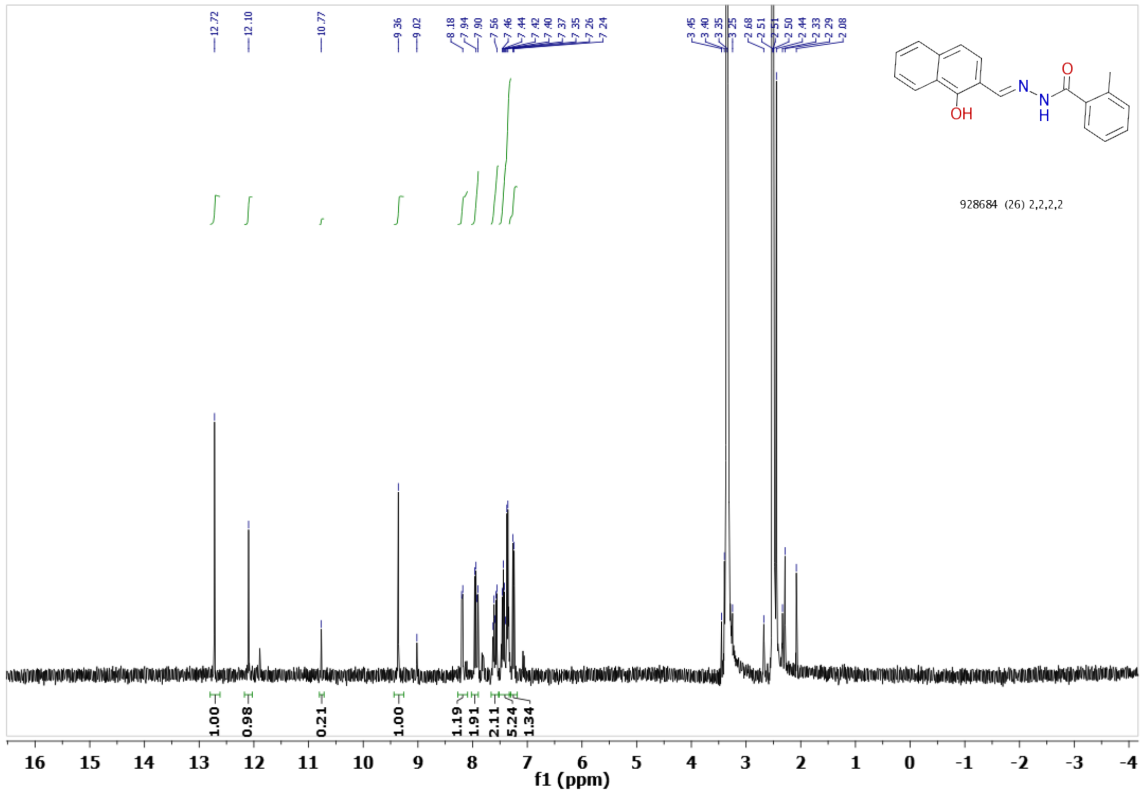

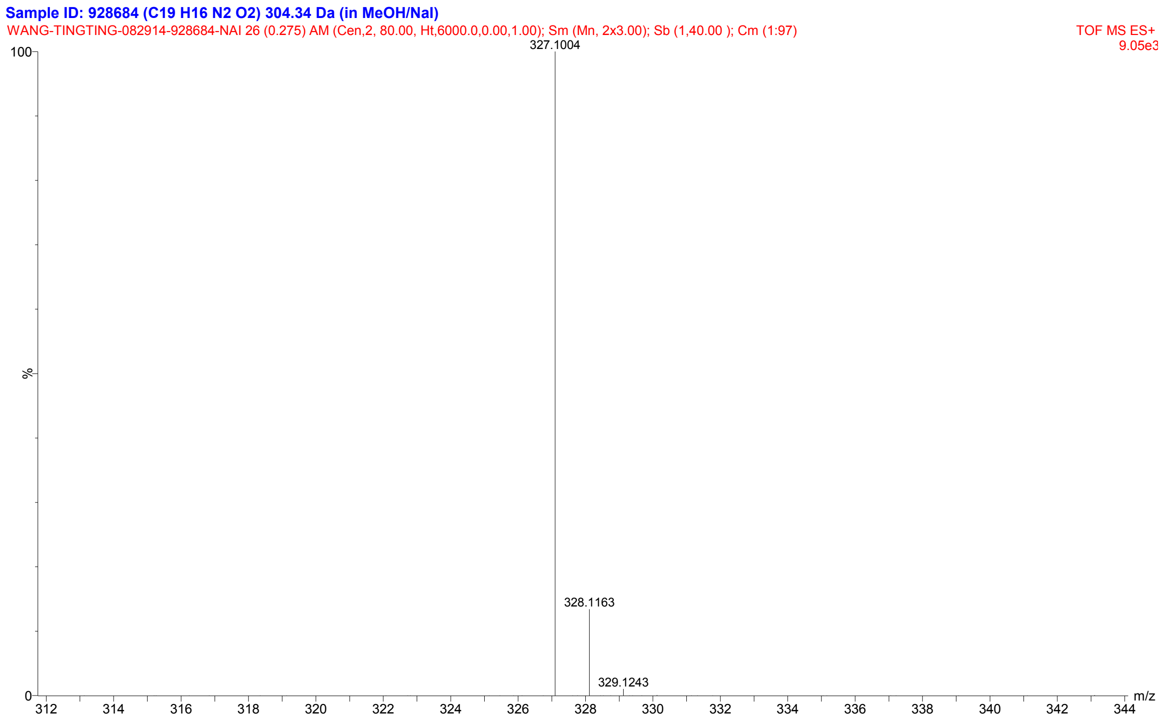
Sample ID: 928684 (C19 H16 N2 O2) 304.34 Da (in MeOH/NaI)
WANG-TINGTING-082914-928684-NAI 26 (0.275) AM (Cen,2, 80.00, Ht,6000.0,0.00,1.00); Sm (Mn, 2x3.00); Sb (1,40.00); Cm (1:97)
TOF MS ES+
9.05e3
327.1004
328.1163
329.1243
m/z

SAR-32

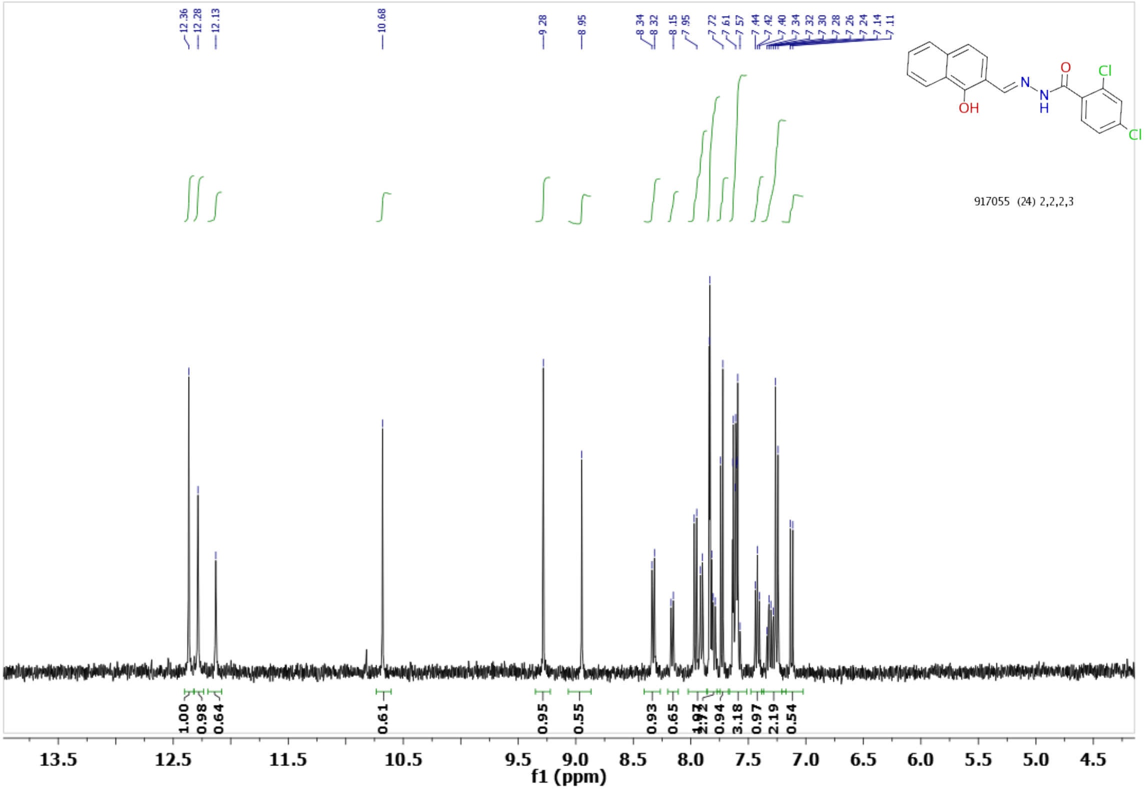

12.36
12.28
12.13
10.68
9.28
8.95
8.34
8.32
8.15
7.95
7.72
7.61
7.57
7.44
7.42
7.40
7.34
7.32
7.30
7.28
7.26
7.24
7.14
7.11
OH
N
N
H
O
Cl
Cl
917055 (24) 2,2,2,3
1.00
0.98
0.64
0.61
0.95
0.55
0.93
0.65
0.94
3.18
0.97
2.19
0.54
13.5
12.5
11.5
10.5
9.5
9.0
8.5
8.0
7.5
7.0
6.5
6.0
5.5
5.0
4.5
f1 (ppm)

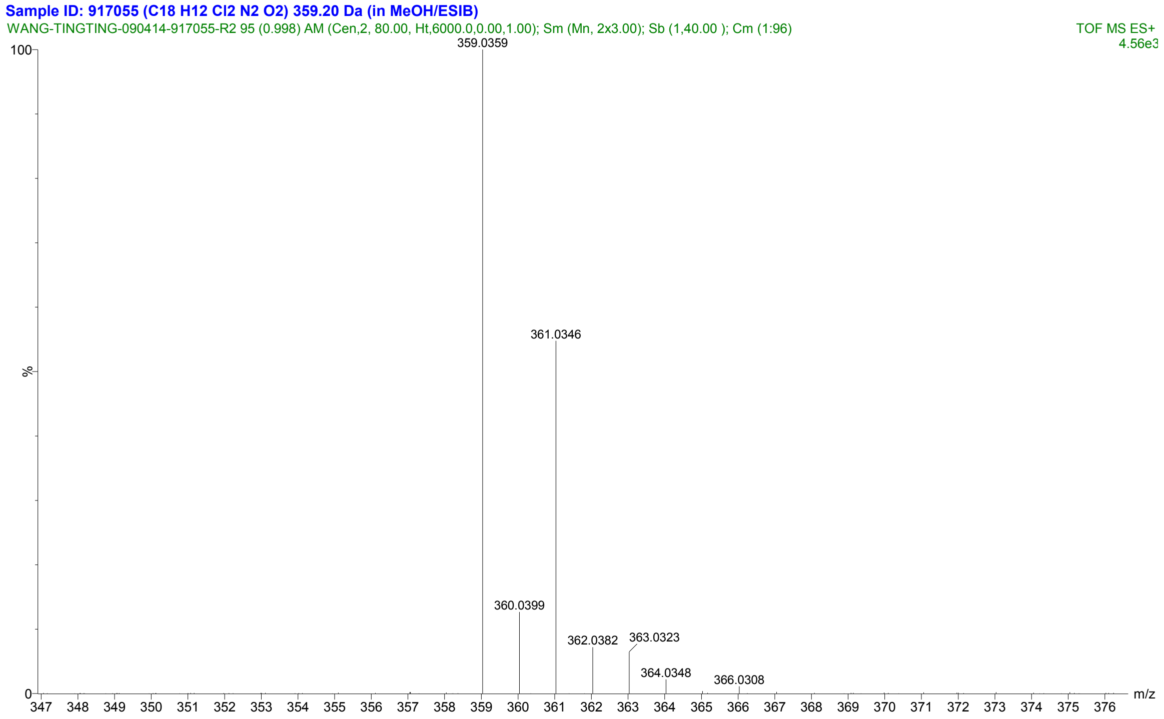

Sample ID: 917055 (C18 H12 Cl2 N2 O2) 359.20 Da (in MeOH/ESIB)
WANG-TINGTING-090414-917055-R2 95 (0.998) AM (Cen,2, 80.00, Ht,6000.0,0.00,1.00); Sm (Mn, 2x3.00); Sb (1,40.00); Cm (1:96)
TOF MS ES+
4.56e3
100
%
0
359.0359
361.0346
360.0399
362.0382
363.0323
364.0348
366.0308
347
348
349
350
351
352
353
354
355
356
357
358
359
360
361
362
363
364
365
366
367
368
369
370
371
372
373
374
375
376
m/z

SAR-33

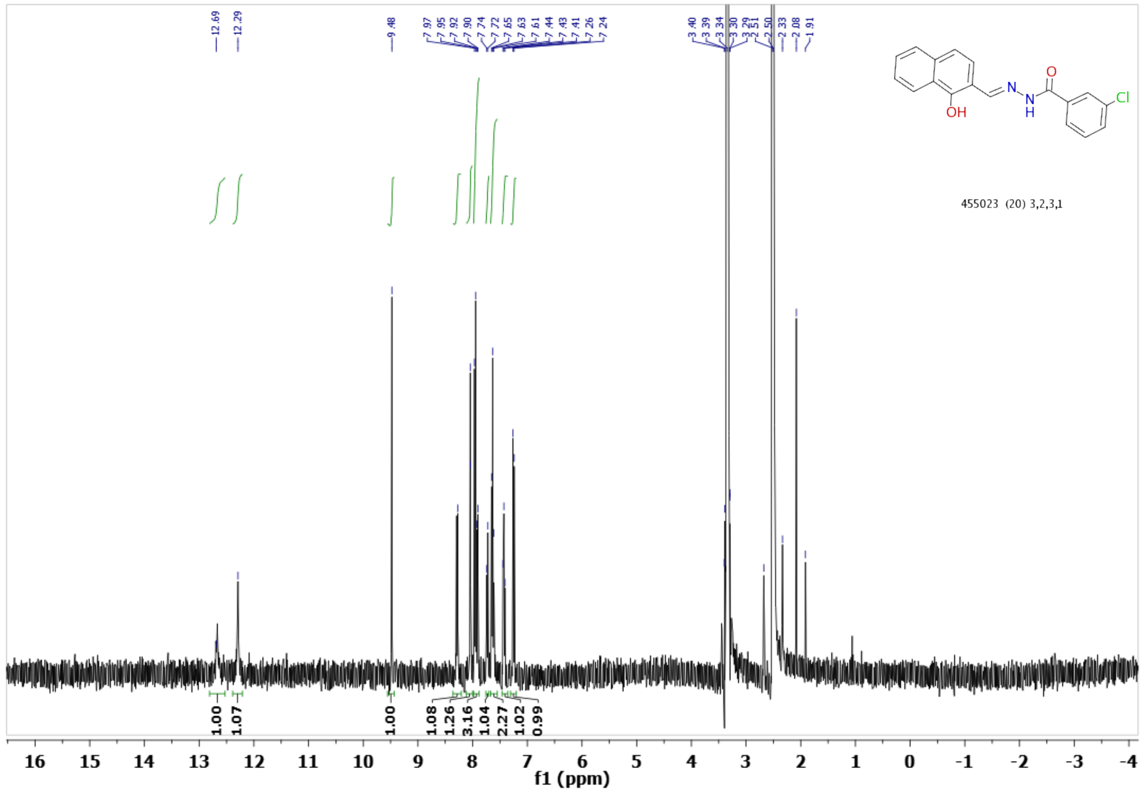

455023 (20) 3,2,3,1
f1 (ppm)

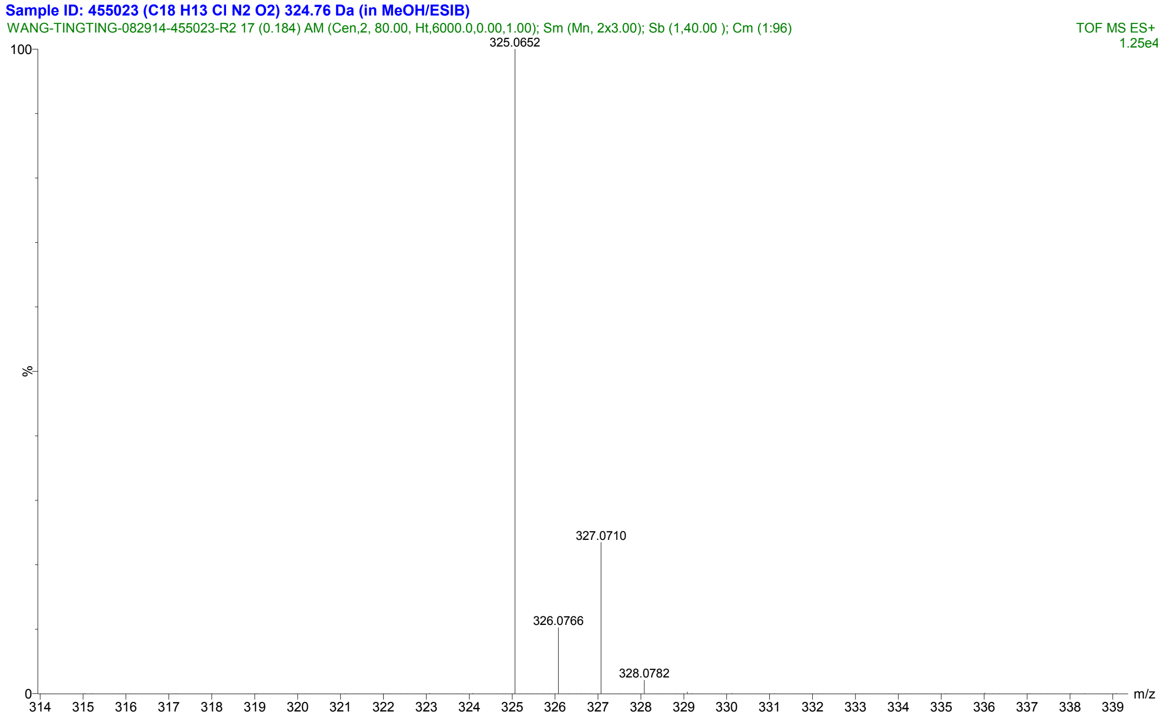

Sample ID: 455023 (C18 H13 Cl N2 O2) 324.76 Da (in MeOH/ESIB)
WANG-TINGTING-082914-455023-R2 17 (0.184) AM (Cen,2, 80.00, Ht,6000.0,0.00,1.00); Sm (Mn, 2x3.00); Sb (1,40.00); Cm (1:96)
TOF MS ES+
1.25e4
325.0652
327.0710
326.0766
328.0782
m/z

SAR-34

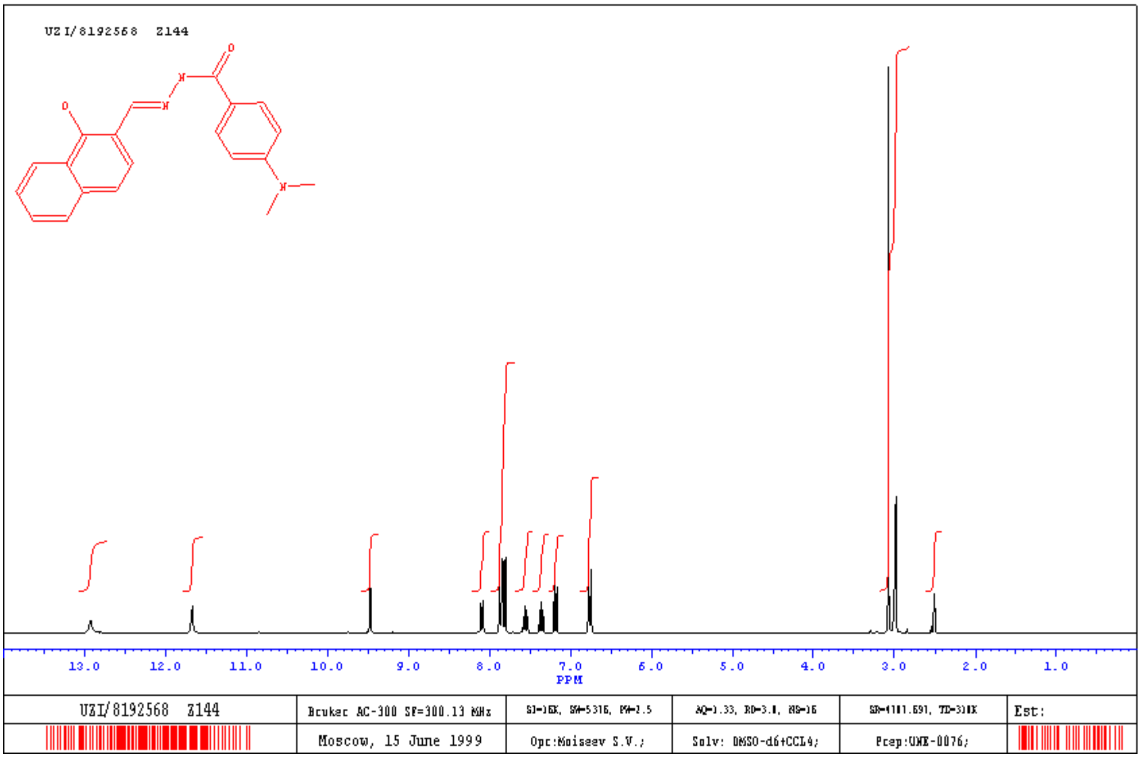

UZI/8192568 Z144
O
N
N
O
N
13.0
12.0
11.0
10.0
9.0
8.0
7.0
PPM
6.0
5.0
4.0
3.0
2.0
1.0
UZI/8192568 Z144
Bruker AC-300 SF=300.13 MHz
Moscow, 15 June 1999
Opr:Moiseev S.V.;
Solv: DMSO-d6+CCL4;
Est:

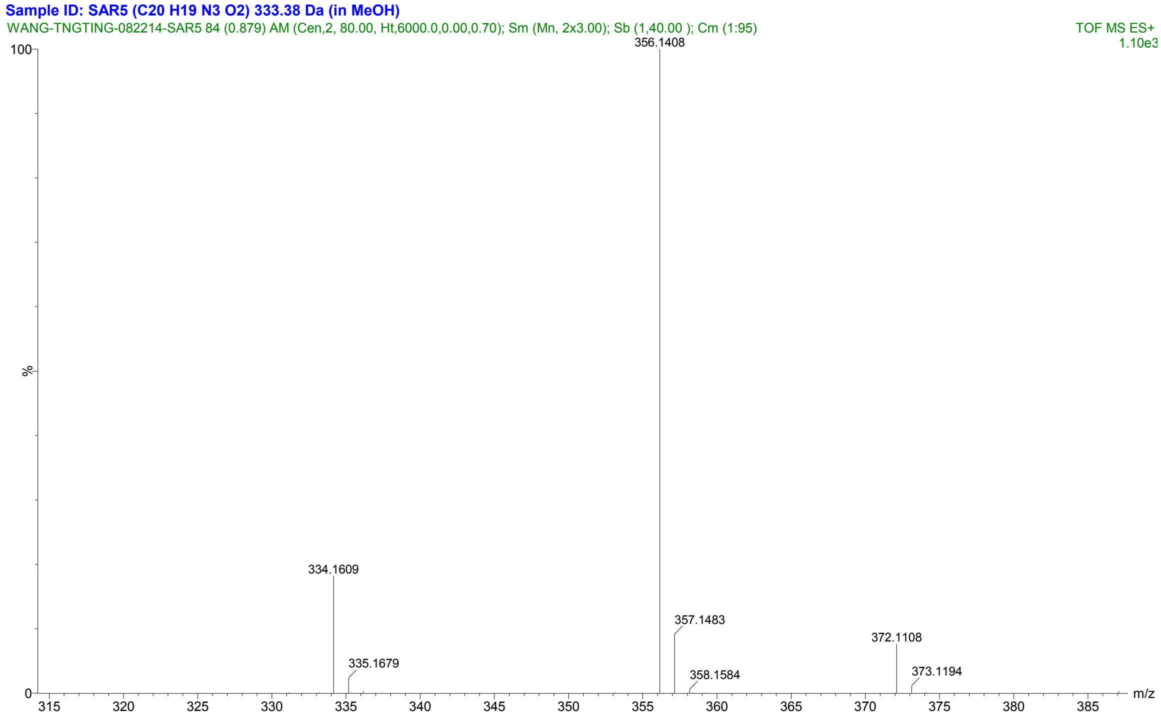

Sample ID: SAR5 (C20 H19 N3 O2) 333.38 Da (in MeOH)
WANG-TNGTING-082214-SAR5 84 (0.879) AM (Cen,2, 80.00, Ht,6000.0,0.00,0.70); Sm (Mn, 2x3.00); Sb (1,40.00); Cm (1:95)
TOF MS ES+
1.10e3
100
%
0
356.1408
334.1609
357.1483
372.1108
335.1679
358.1584
373.1194
315
320
325
330
335
340
345
350
355
360
365
370
375
380
385
m/z

SAR-35

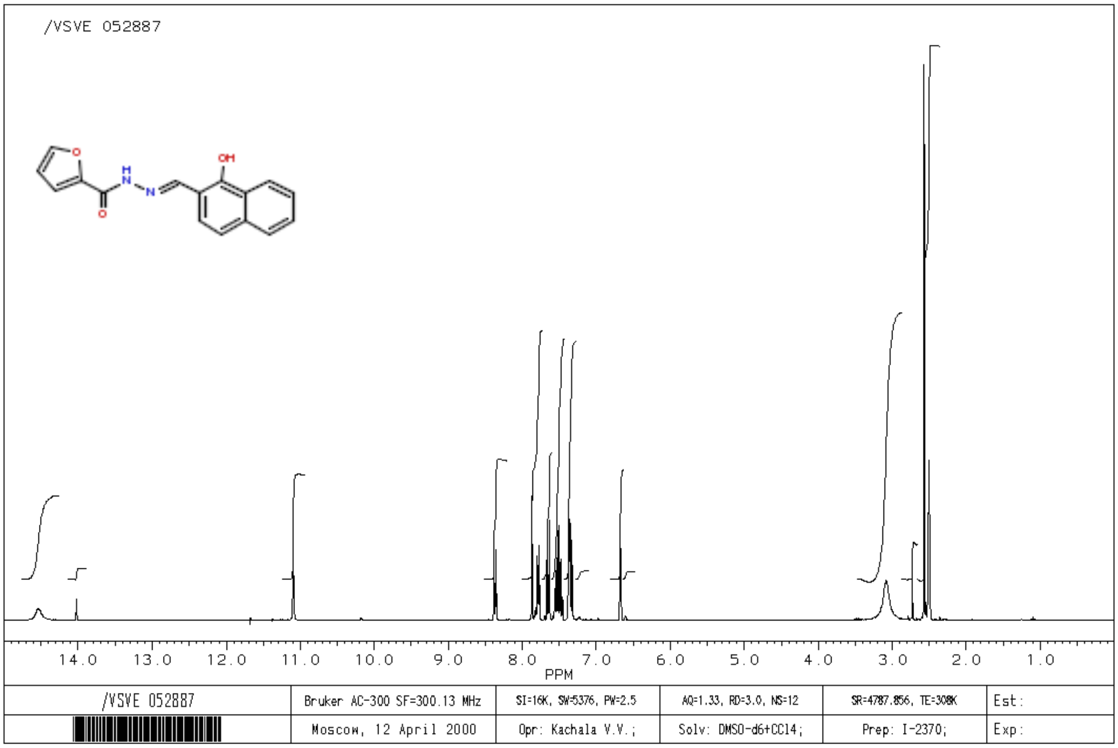

/VSVE 052887
OH
H
N
N
O
O
14.0 13.0 12.0 11.0 10.0 9.0 8.0 7.0 6.0 5.0 4.0 3.0 2.0 1.0
PPM
/VSVE 052887
Bruker AC-300 SF=300.13 MHz
SI=16K, SW=5376, PW=2.5
AQ=1.33, RD=3.0, NS=12
SR=4787.856, TE=308K
Est:
Moscow, 12 April 2000
Opr: Kachala V.V.;
Solv: DMSO-d6+CCl4;
Prep: I-2370;
Exp:

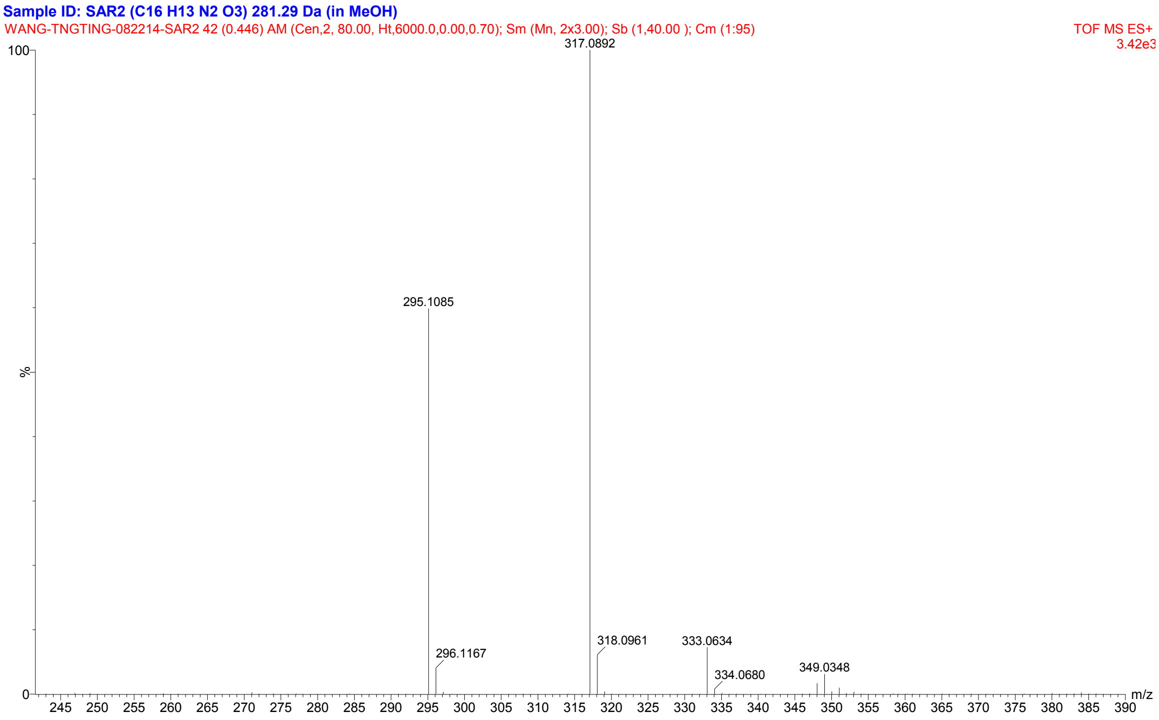

Sample ID: SAR2 (C16 H13 N2 O3) 281.29 Da (in MeOH)
WANG-TNGTING-082214-SAR2 42 (0.446) AM (Cen,2, 80.00, Ht,6000.0,0.00,0.70); Sm (Mn, 2x3.00); Sb (1,40.00); Cm (1:95)
TOF MS ES+
3.42e3
100
%
0
317.0892
295.1085
296.1167
318.0961
333.0634
334.0680
349.0348
245 250 255 260 265 270 275 280 285 290 295 300 305 310 315 320 325 330 335 340 345 350 355 360 365 370 375 380 385 390
m/z

SAR-36

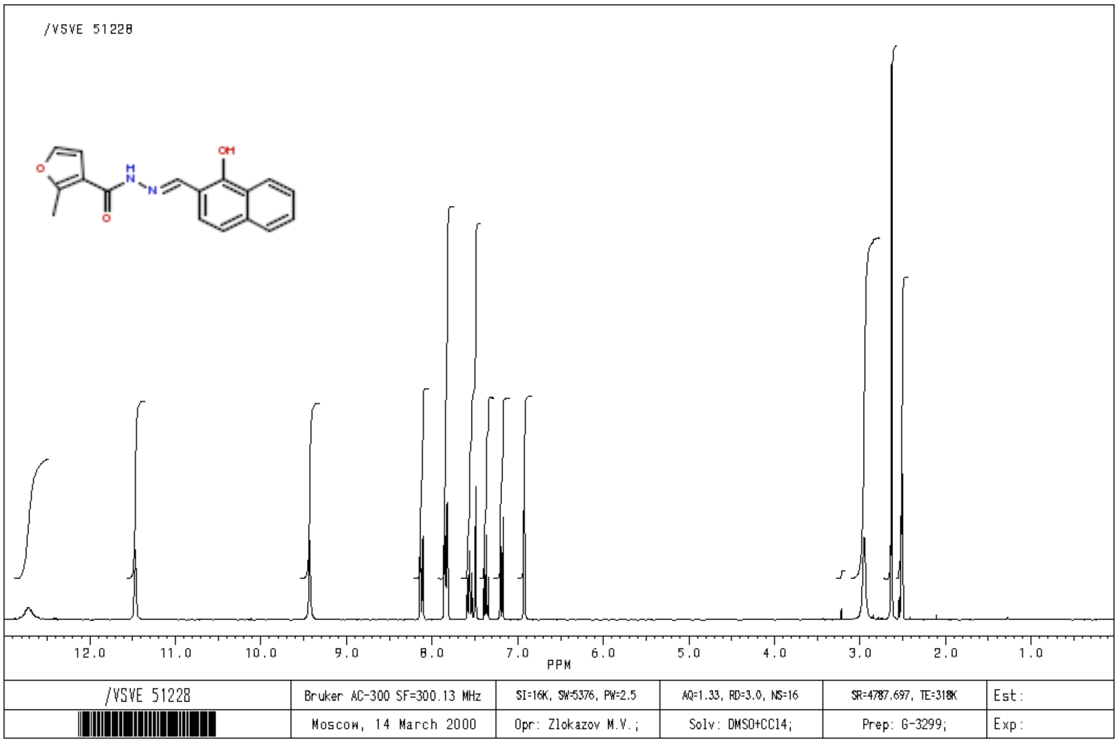

/VSVE 51228
OH
H
N
N
O
O
12.0
11.0
10.0
9.0
8.0
7.0
6.0
5.0
4.0
3.0
2.0
1.0
PPM
/VSVE 51228
Bruker AC-300 SF=300.13 MHz
SI=16K, SW=5376, PW=2.5
AQ=1.33, RD=3.0, NS=16
SR=4787.697, TE=318K
Est:
Moscow, 14 March 2000
Opr: Zlokazov M.V.;
Solv: DMSO+CCl4;
Prep: G-3299;
Exp:

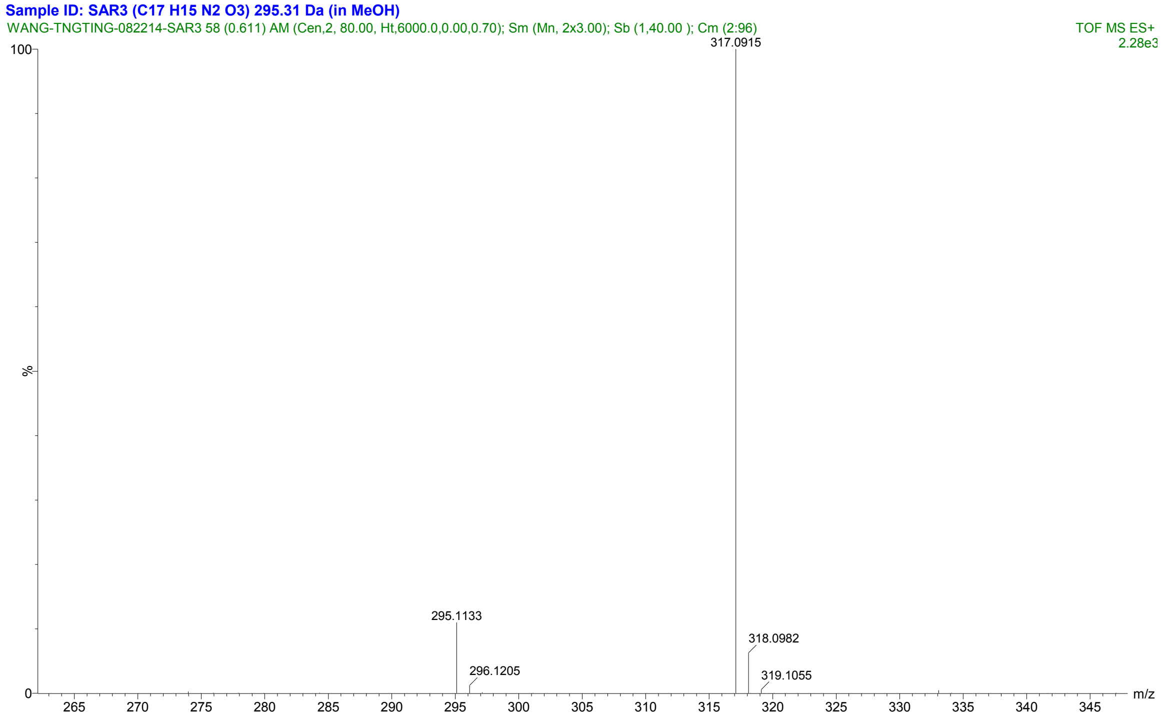

Sample ID: SAR3 (C17 H15 N2 O3) 295.31 Da (in MeOH)
WANG-TNGTING-082214-SAR3 58 (0.611) AM (Cen,2, 80.00, Ht,6000.0,0.00,0.70); Sm (Mn, 2x3.00); Sb (1,40.00); Cm (2:96)
TOF MS ES+
2.28e3
100
%
0
317.0915
295.1133
318.0982
296.1205
319.1055
265
270
275
280
285
290
295
300
305
310
315
320
325
330
335
340
345
m/z

SAR-37

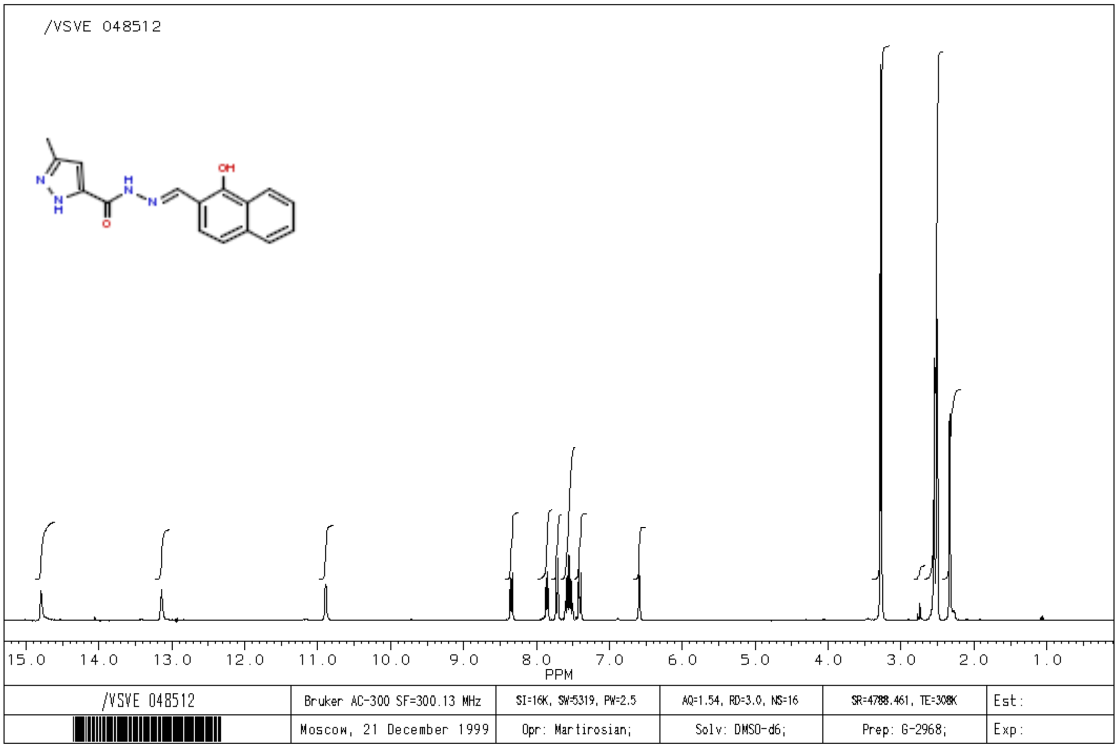
/VSVE 048512
OH
N
NH
H
N
N
O
15.0 14.0 13.0 12.0 11.0 10.0 9.0 8.0 7.0 6.0 5.0 4.0 3.0 2.0 1.0
PPM
/VSVE 048512
Bruker AC-300 SF=300.13 MHz
SI=16K, SW=5319, PW=2.5
AQ=1.54, RD=3.0, NS=16
SR=4788.461, TE=308K
Est:
Moscow, 21 December 1999
Opr: Martirosian;
Solv: DMSO-d6;
Prep: G-2968;
Exp:

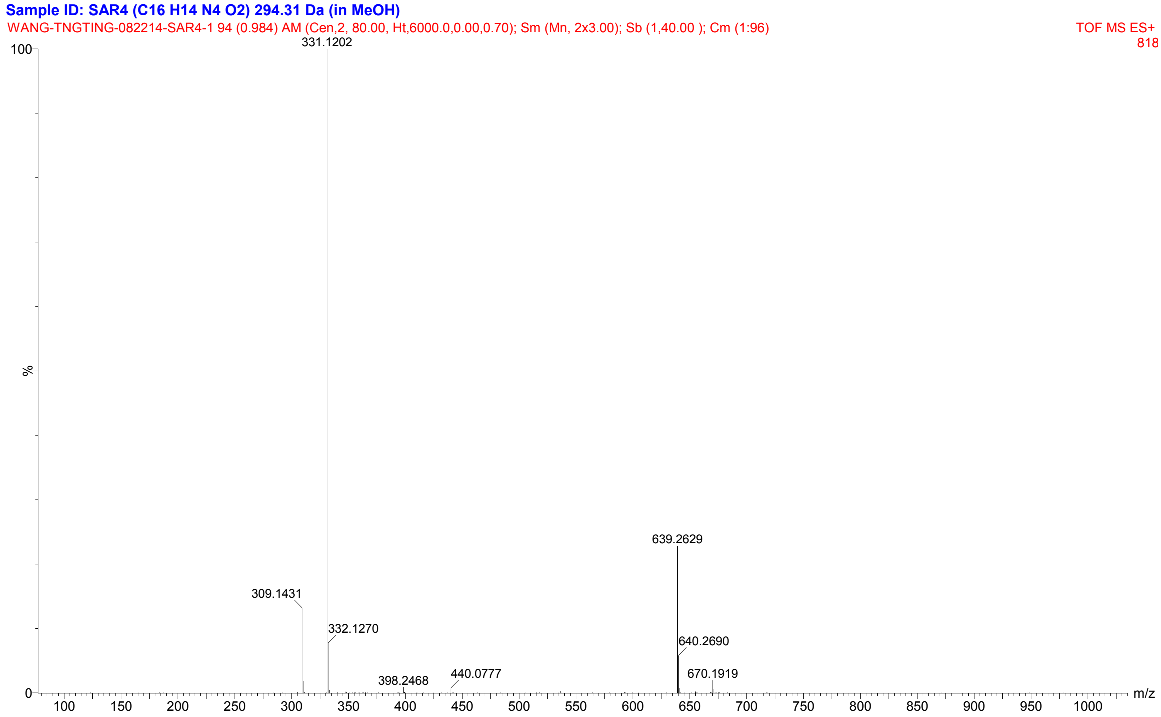
Sample ID: SAR4 (C16 H14 N4 O2) 294.31 Da (in MeOH)
WANG-TNGTING-082214-SAR4-1 94 (0.984) AM (Cen,2, 80.00, Ht,6000.0,0.00,0.70); Sm (Mn, 2x3.00); Sb (1,40.00); Cm (1:96)
TOF MS ES+
818
100
%
0
331.1202
309.1431
332.1270
398.2468
440.0777
639.2629
640.2690
670.1919
100 150 200 250 300 350 400 450 500 550 600 650 700 750 800 850 900 950 1000
m/z

SAR-38

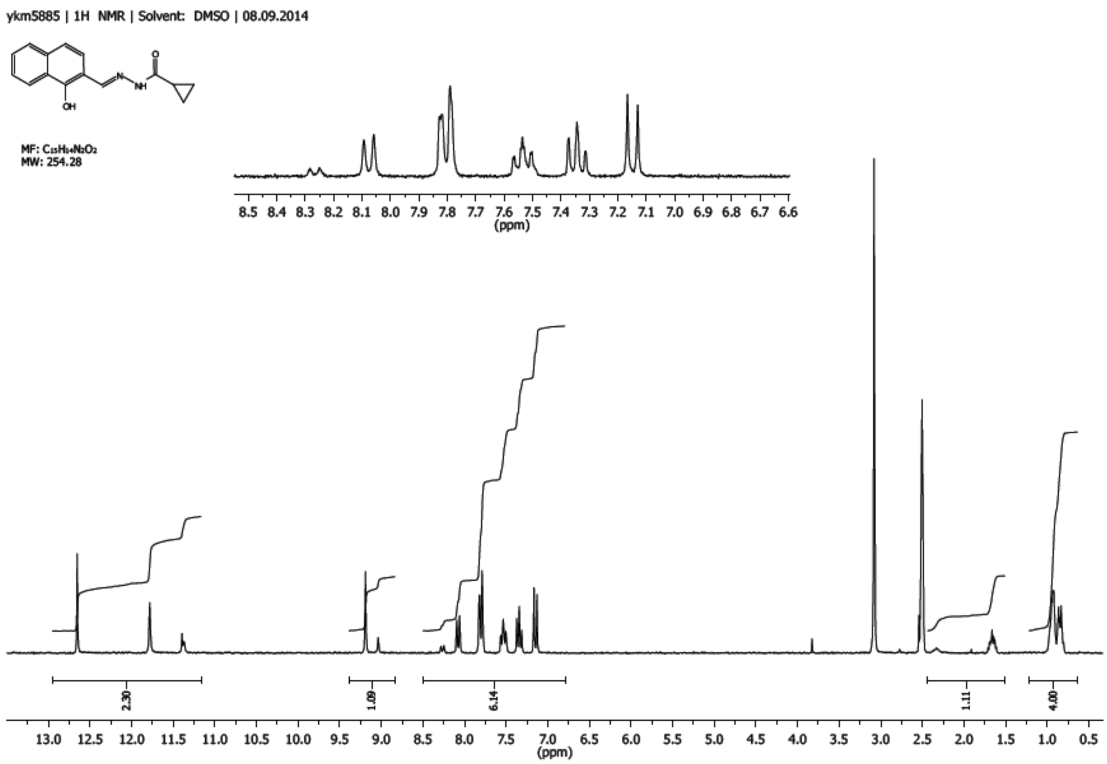
ykm5885 | 1H NMR | Solvent: DMSO | 08.09.2014
OH
NH
O
MF: C15H14N2O2
MW: 254.28
(ppm)
2.30
1.09
6.14
1.11
4.00
(ppm)

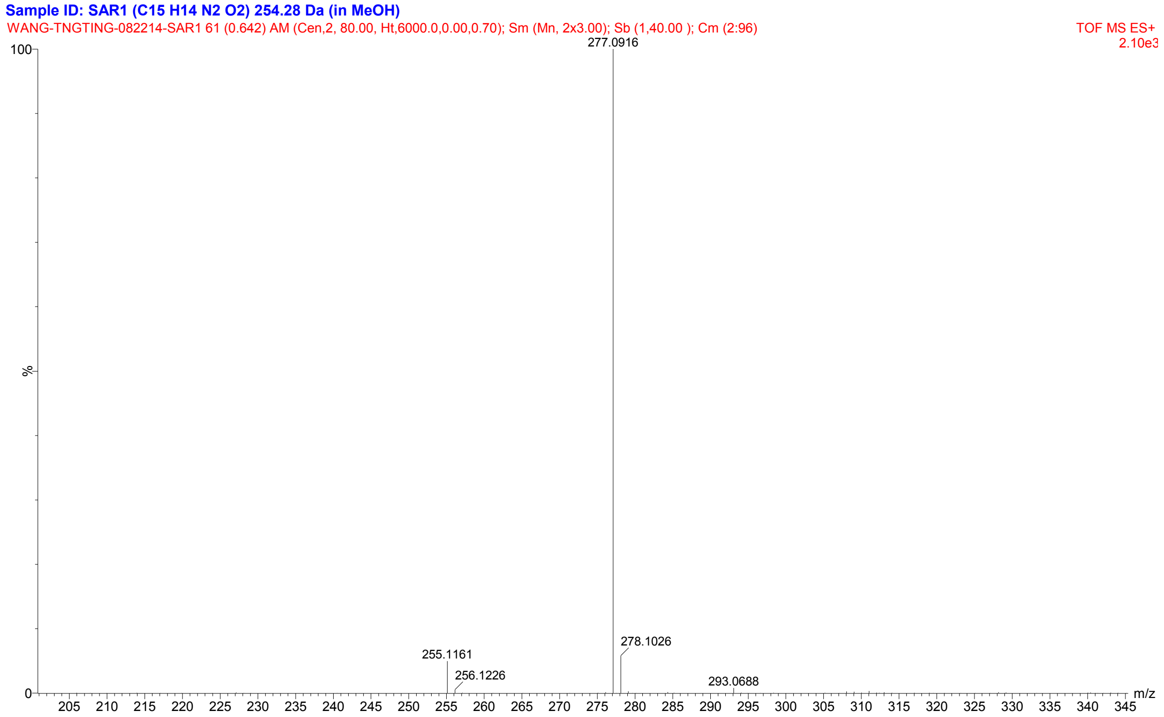
Sample ID: SAR1 (C15 H14 N2 O2) 254.28 Da (in MeOH)
WANG-TNGTING-082214-SAR1 61 (0.642) AM (Cen,2, 80.00, Ht,6000.0,0.00,0.70); Sm (Mn, 2x3.00); Sb (1,40.00); Cm (2:96)
TOF MS ES+
2.10e3
277.0916
255.1161
256.1226
278.1026
293.0688
%
m/z

SAR-39

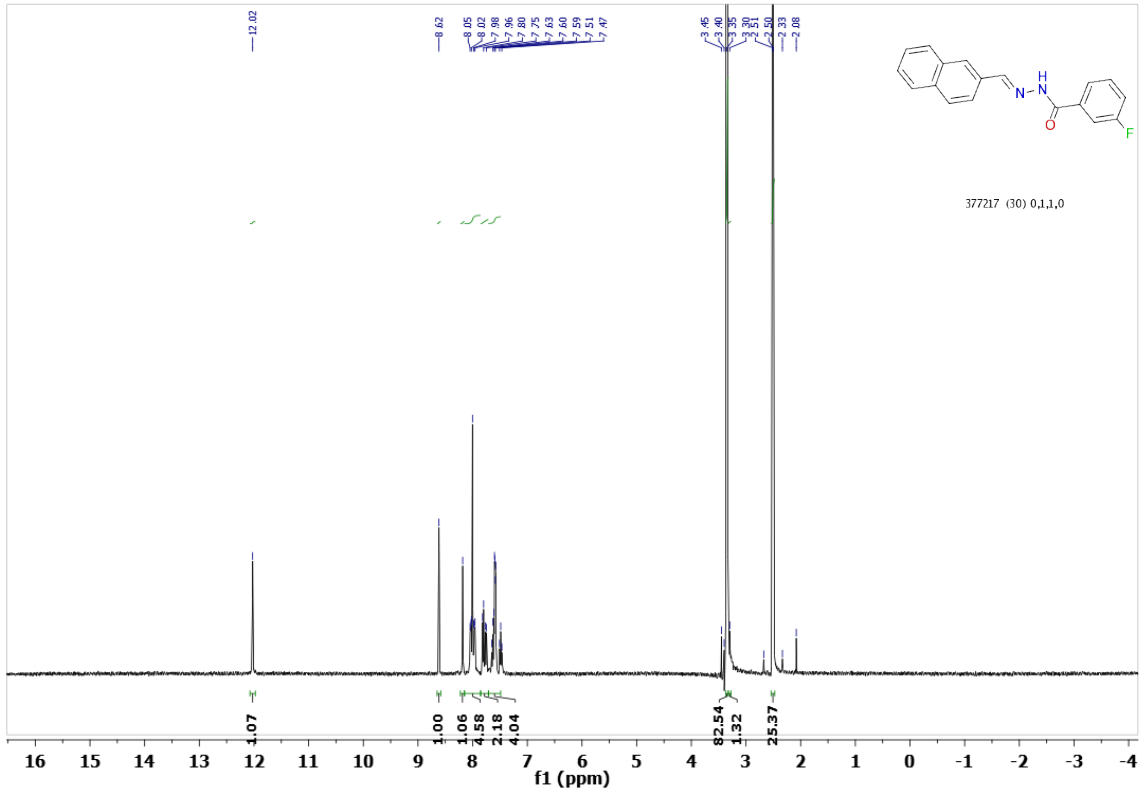

377217 (30) 0,1,1,0
f1 (ppm)

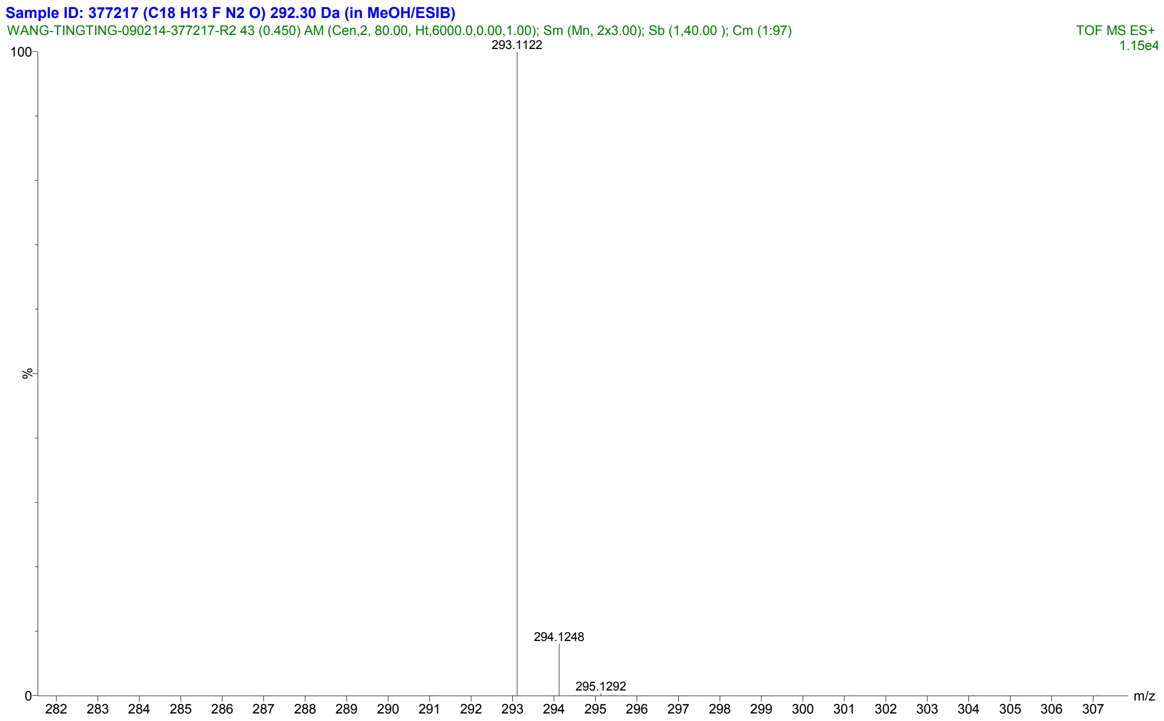

Sample ID: 377217 (C18 H13 F N2 O) 292.30 Da (in MeOH/ESIB)
WANG-TINGTING-090214-377217-R2 43 (0.450) AM (Cen,2, 80.00, Ht,6000.0,0.00,1.00); Sm (Mn, 2x3.00); Sb (1,40.00); Cm (1:97)
TOF MS ES+
1.15e4
293.1122
294.1248
295.1292
m/z

SAR-40

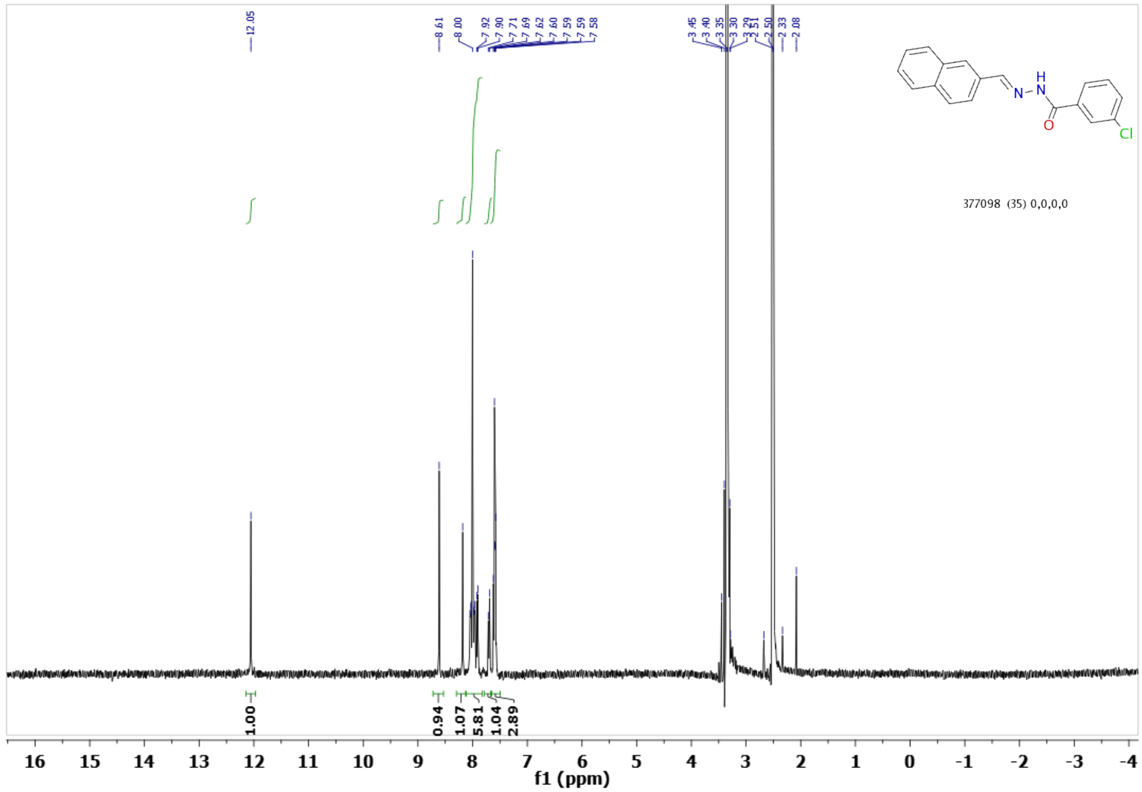
12.05
8.61
8.00
7.92
7.90
7.71
7.69
7.62
7.60
7.59
7.59
7.58
3.45
3.40
3.35
3.30
3.29
2.51
2.50
2.33
2.08
377098 (35) 0,0,0,0
1.00
0.94
1.07
5.81
1.04
2.89
f1 (ppm)
H
N
N
O
Cl

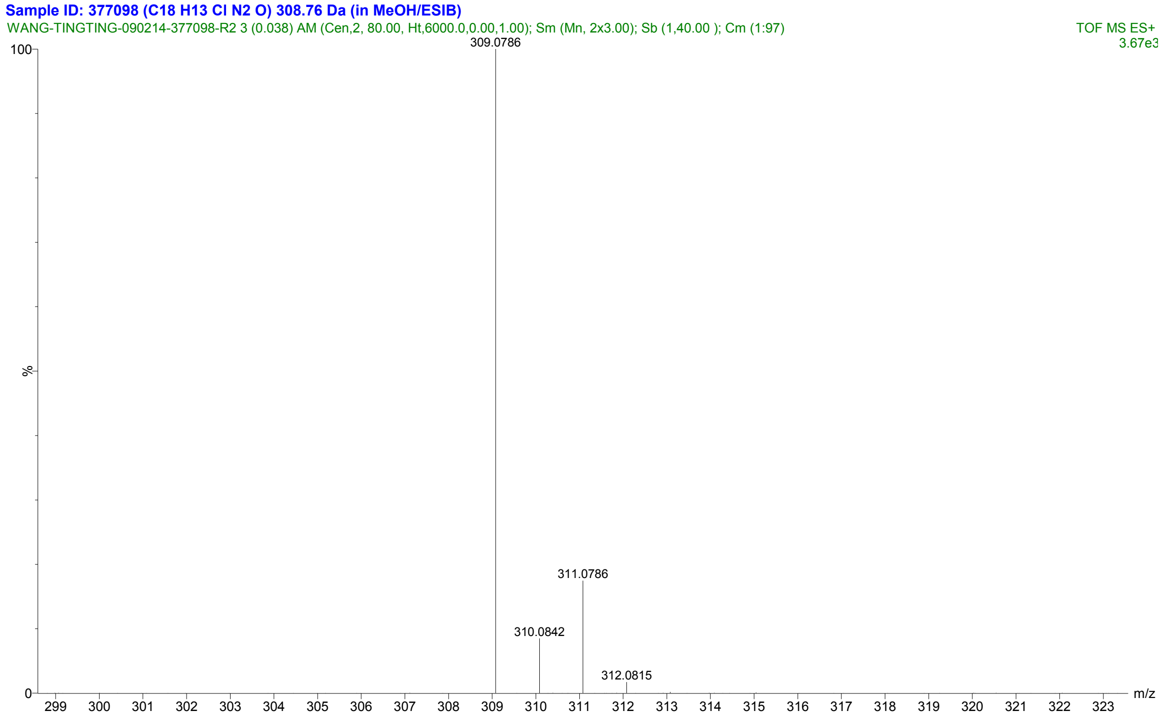
Sample ID: 377098 (C18 H13 Cl N2 O) 308.76 Da (in MeOH/ESIB)
WANG-TINGTING-090214-377098-R2 3 (0.038) AM (Cen,2, 80.00, Ht,6000.0,0.00,1.00); Sm (Mn, 2x3.00); Sb (1,40.00); Cm (1:97)
TOF MS ES+
3.67e3
309.0786
311.0786
310.0842
312.0815
%
m/z

SAR-41

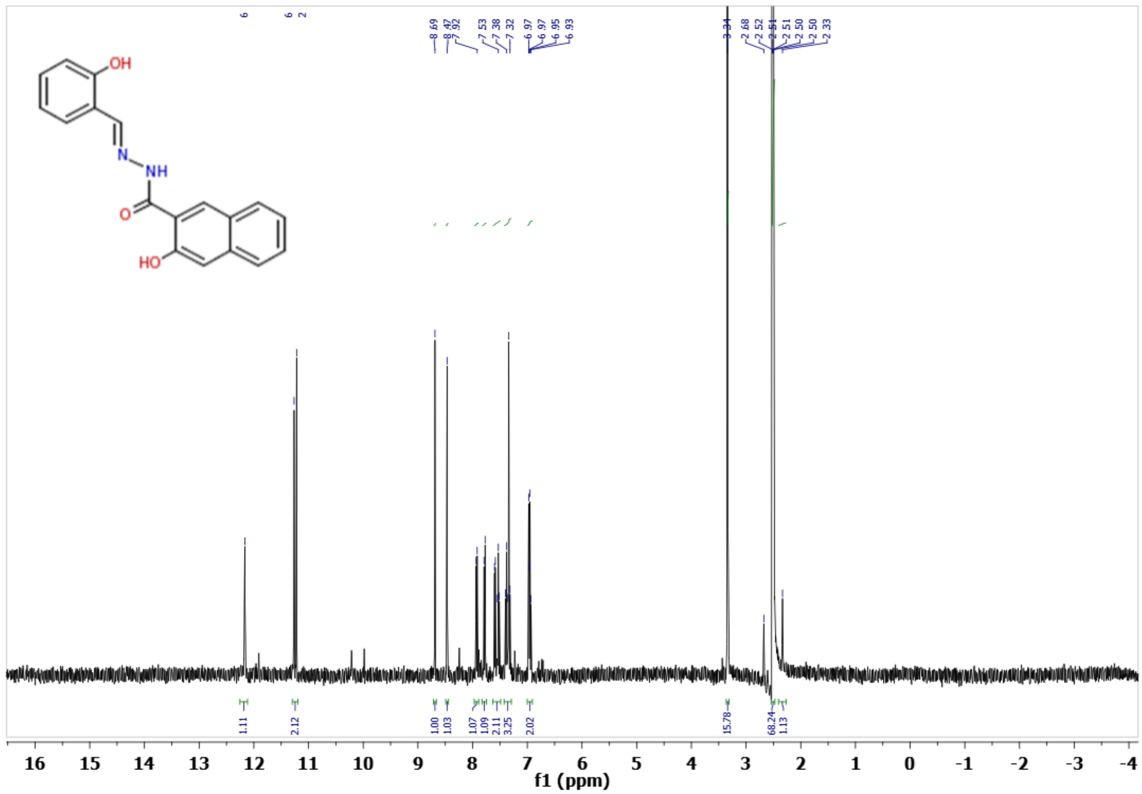

Sample ID: 121167 (C18 H14 N2 O3) 306.31 Da (in MeOH/ESIB)

WANG-TINGTING-090214-121167-R2-CV10 92 (0.954) AM (Cen,2, 80.00, Ht,6000.0,0.00,1.00); Sm (Mn, 2x3.00); Sb (1,40.00); Cm (1:97)

TOF MS ES+
1.16e3

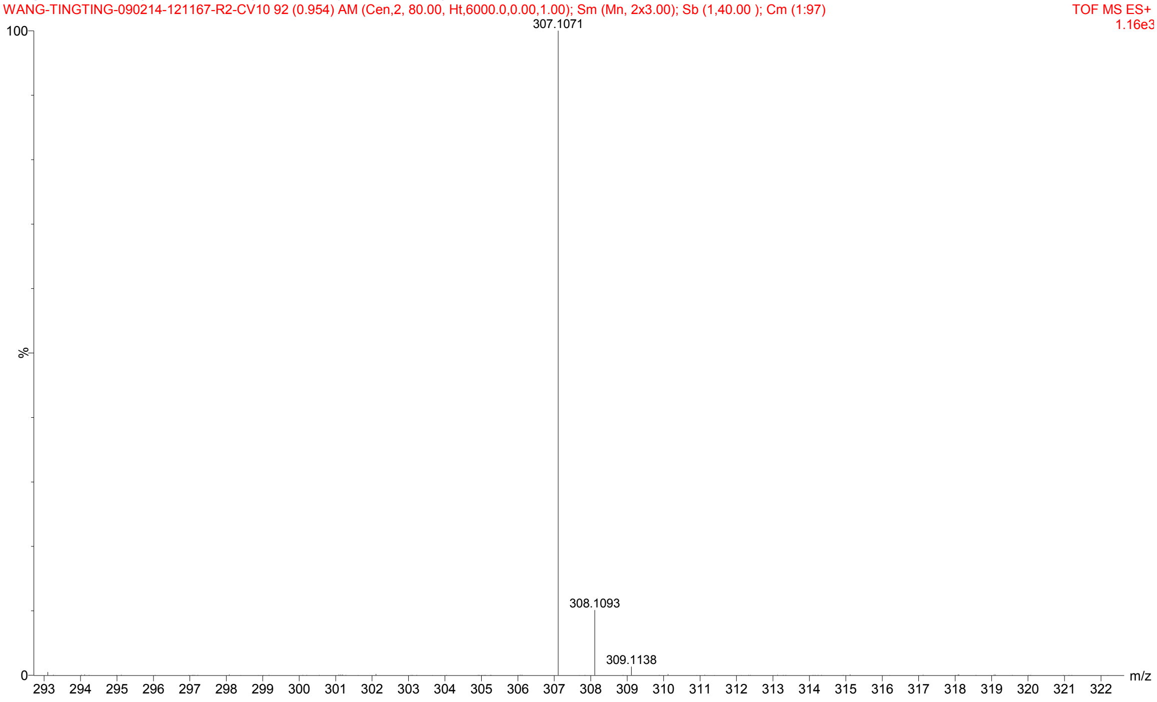

SAR-42

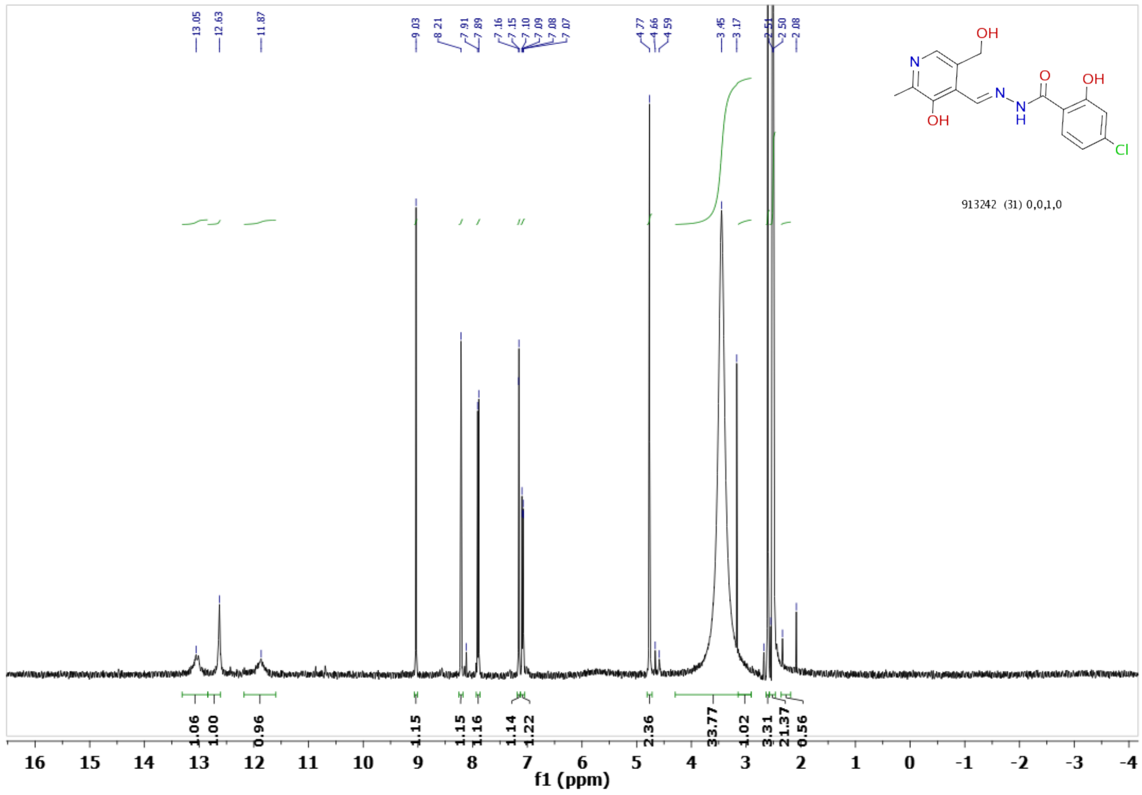

Sample ID: 913242 (C15 H14 Cl N3 O4) 335.74 Da (in MeOH/ESIB)
WANG-TINGTING-090414-913242-R2-CV10 20 (0.214) AM (Cen,2, 80.00, Ht,6000.0,0.00,1.00); Sm (Mn, 2x3.00); Sb (1,40.00); Cm (1:97)

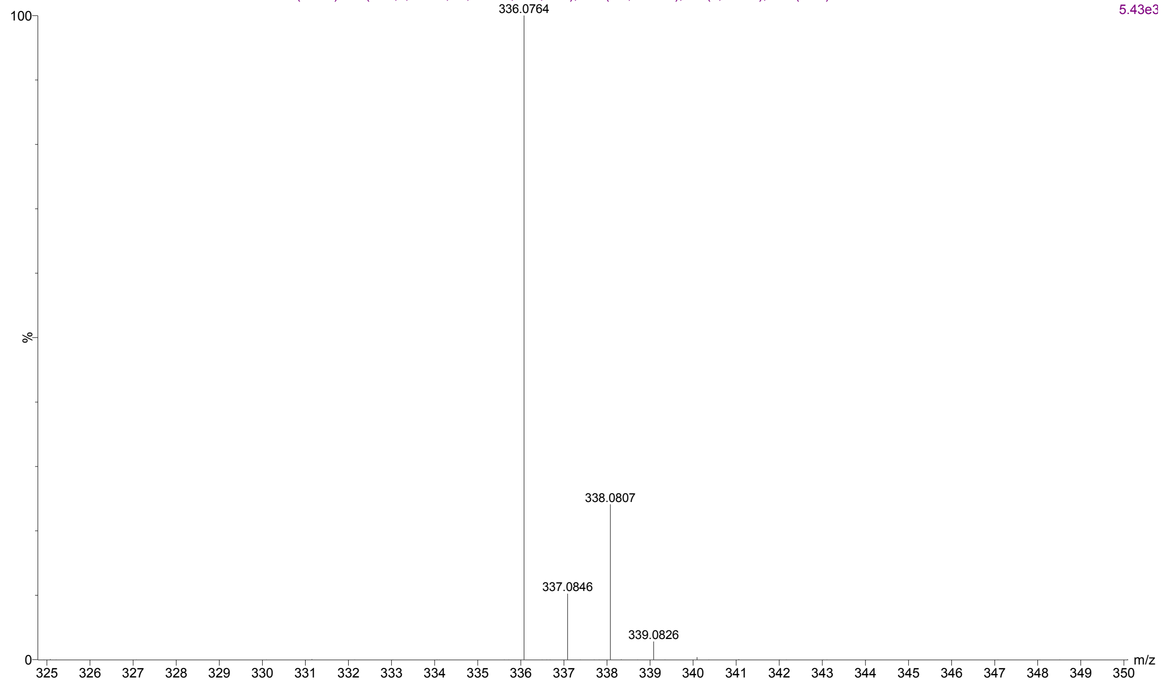

SAR-43

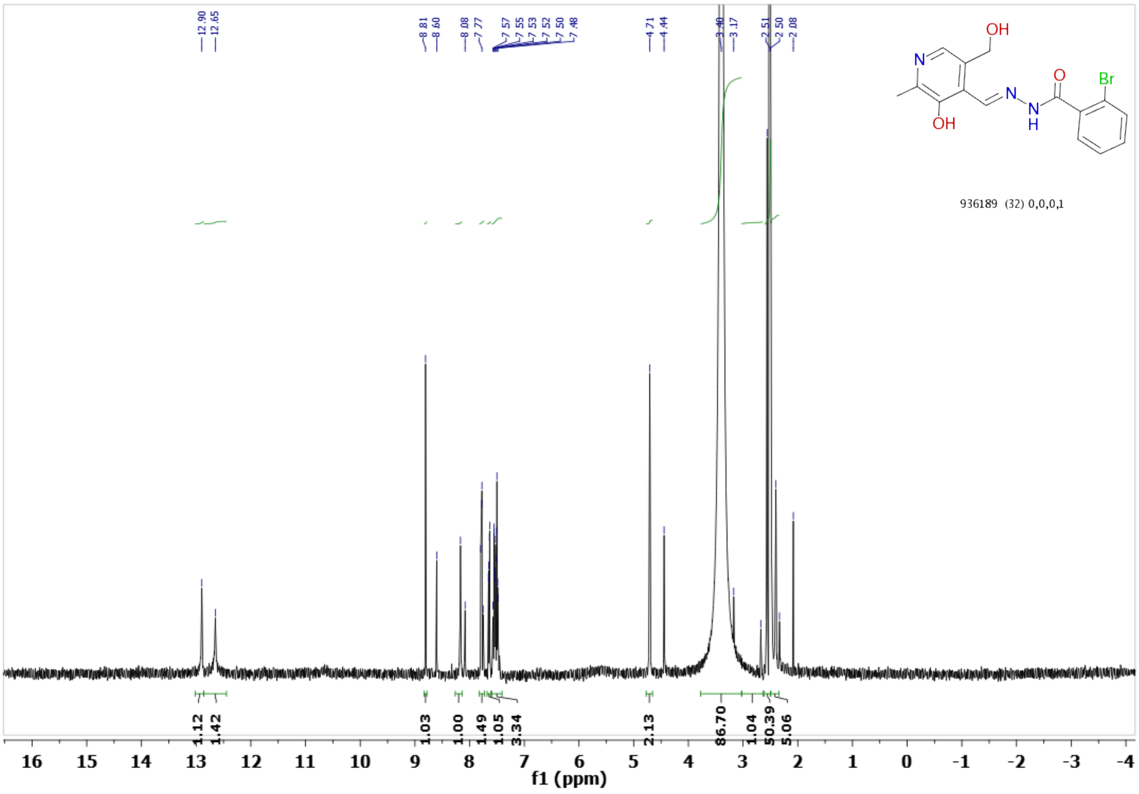

Sample ID: 936189 (C15 H14 Br N3 O3) 364.19 Da (in MeOH/ESIB)

WANG-TINGTING-090414-936189-R2-CV10 1 (0.018) AM (Cen,2, 80.00, Ht,6000.0,0.00,1.00); Sm (Mn, 2x3.00); Sb (1,40.00); Cm (1:96)

TOF MS ES+
7.57e3

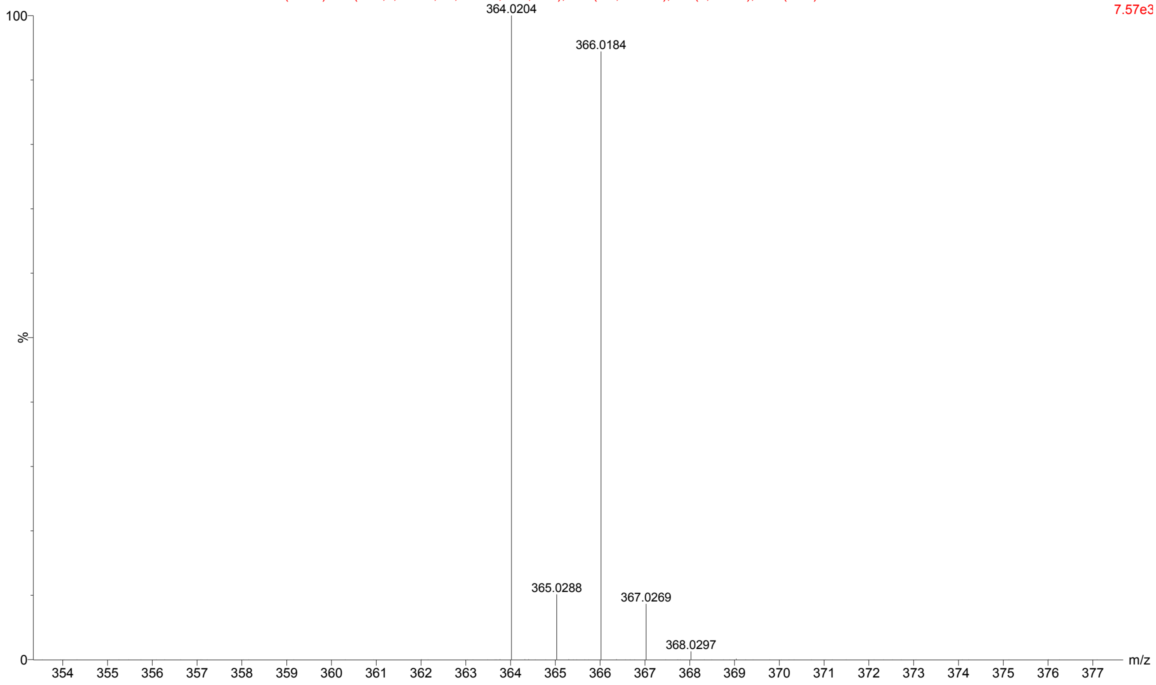

SAR-44

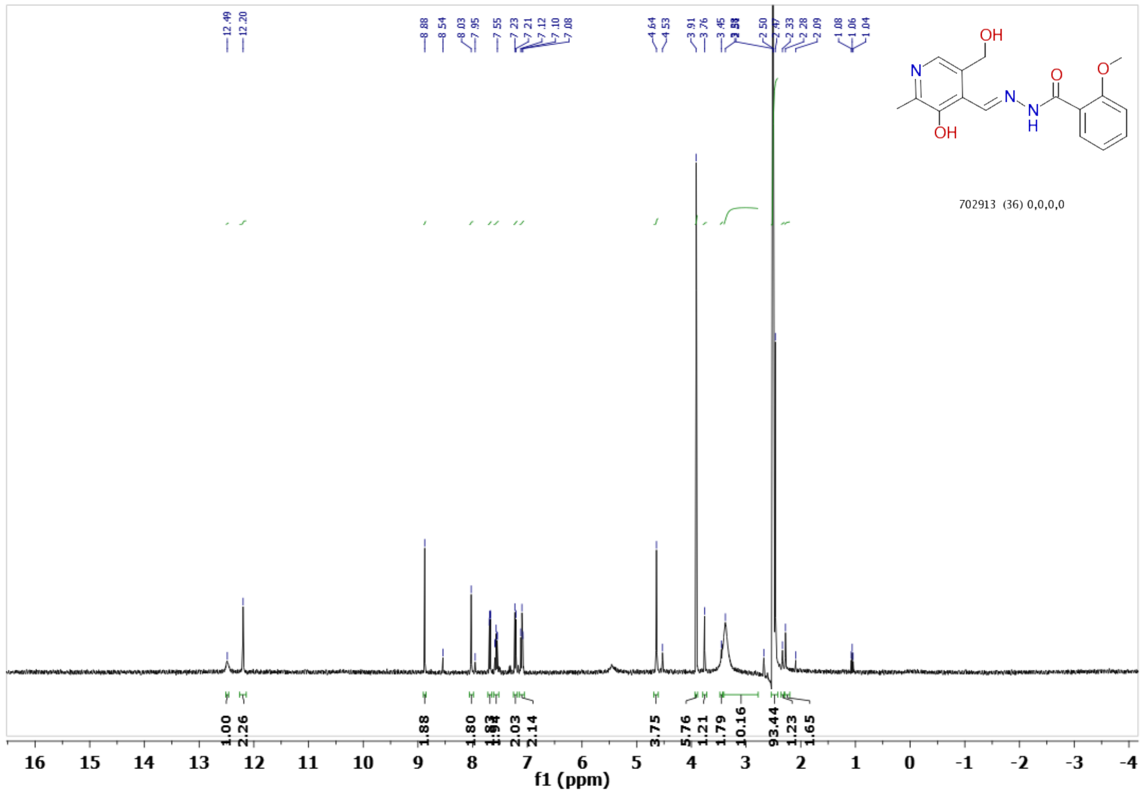

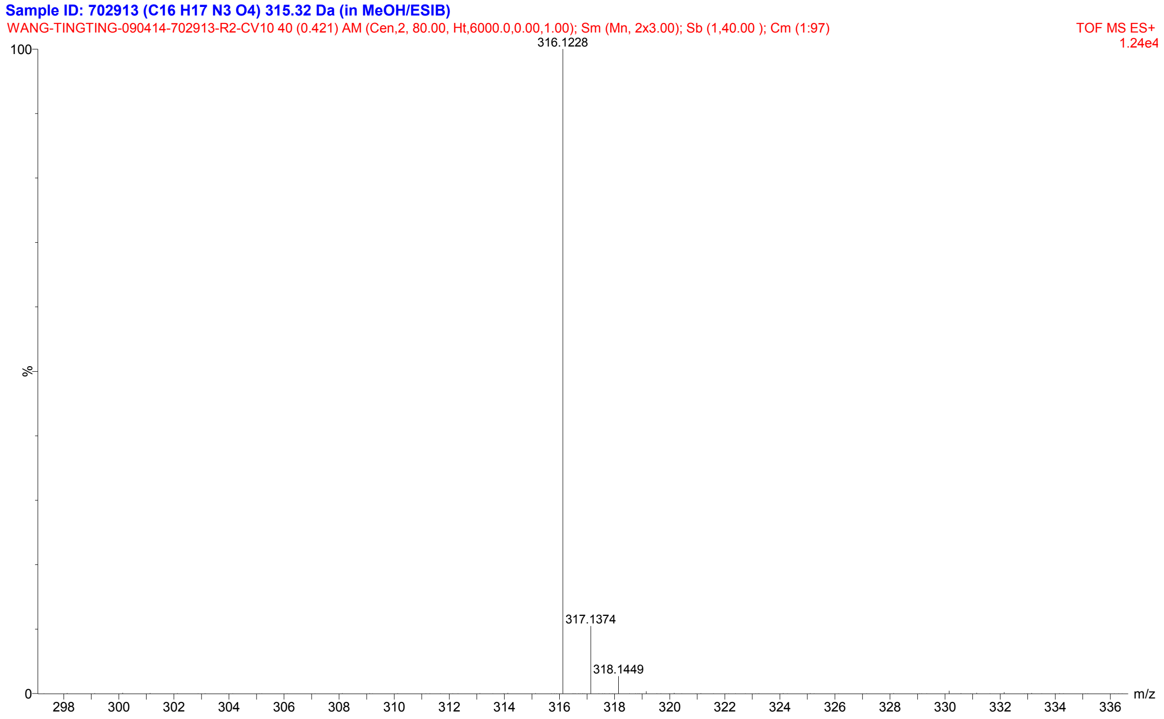
Sample ID: 702913 (C16 H17 N3 O4) 315.32 Da (in MeOH/ESIB)
WANG-TINGTING-090414-702913-R2-CV10 40 (0.421) AM (Cen,2, 80.00, Ht,6000.0,0.00,1.00); Sm (Mn, 2x3.00); Sb (1,40.00); Cm (1:97)
TOF MS ES+
1.24e4
316.1228
317.1374
318.1449
%
m/z

SAR-45

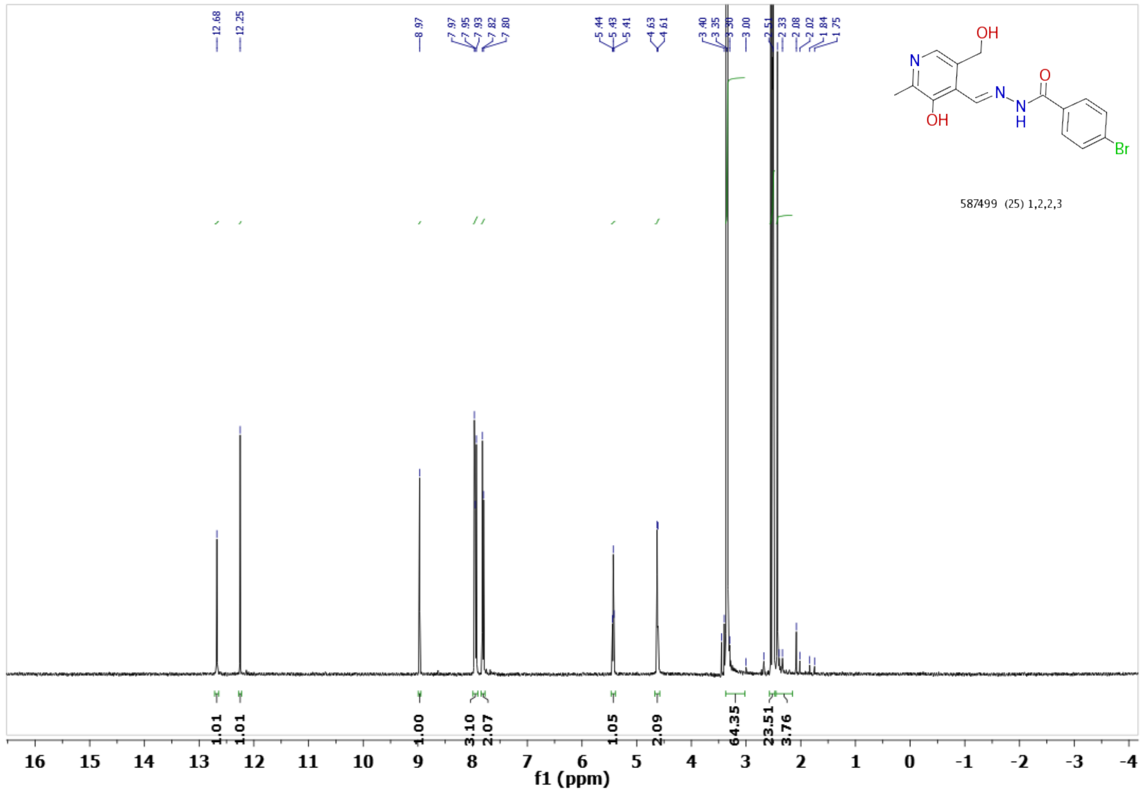

Sample ID: 587499 (C15 H14 Br N3 O3) 364.19 Da (in MeOH/ESIB)

WANG-TINGTING-082914-587499-CV10 76 (0.798) AM (Cen,2, 80.00, Ht,6000.0,0.00,1.00); Sm (Mn, 2x3.00); Sb (1,40.00); Cm (1:96)

TOF MS ES+
1.72e4

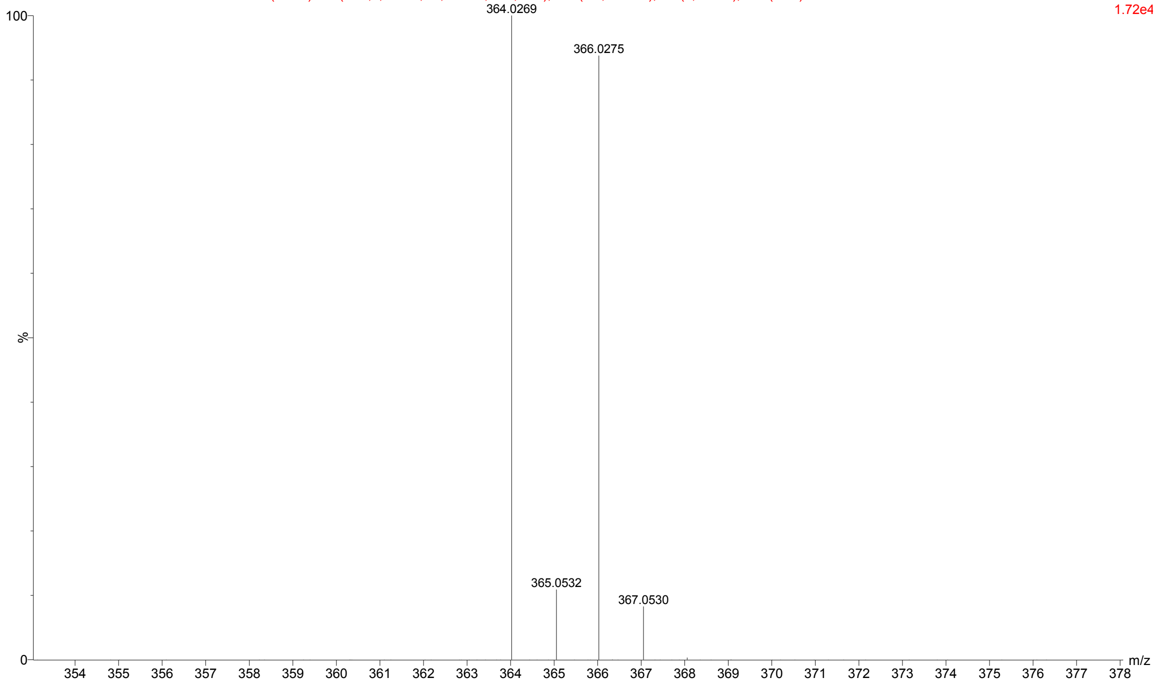

SAR-46

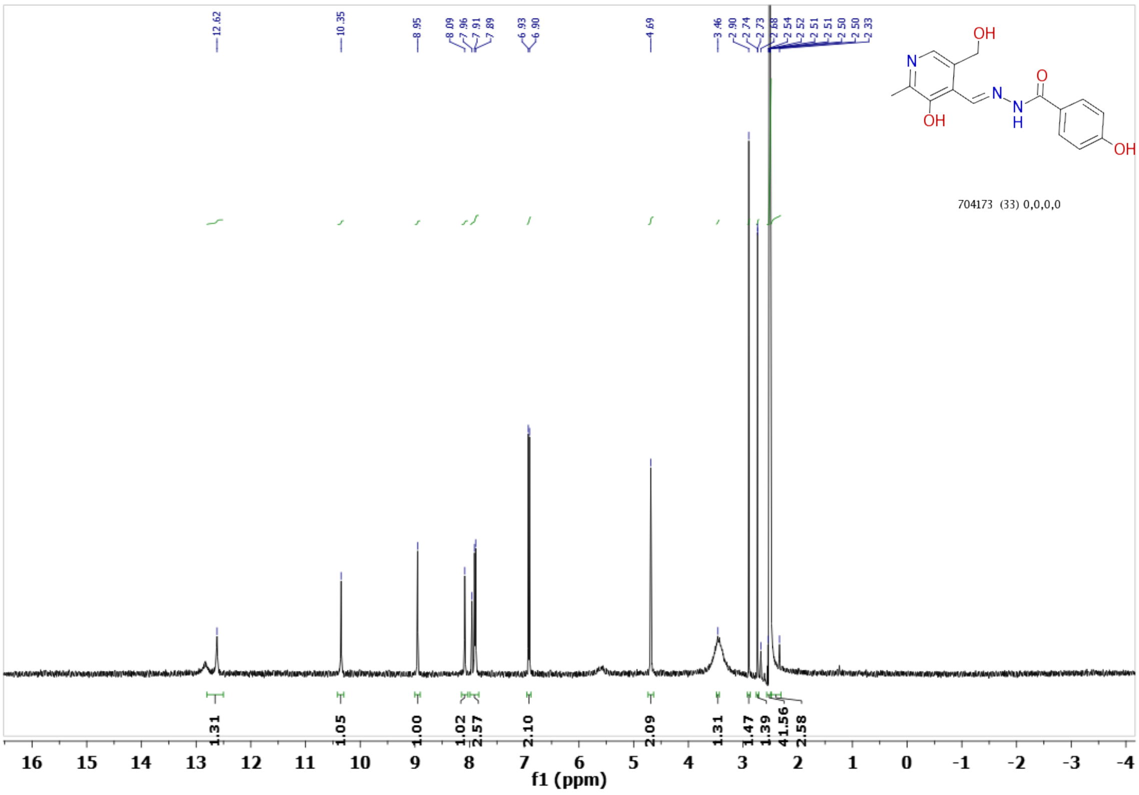

Sample ID: 704173 (C15 H15 N3 O4) 301.29 Da (in MeOH/ESIB)

WANG-TINGTING-082914-704173-R2-CV10 61 (0.640) AM (Cen,2, 80.00, Ht,6000.0,0.00,1.00); Sm (Mn, 2x3.00); Sb (1,40.00); Cm (1:96)

TOF MS ES+
2.30e4

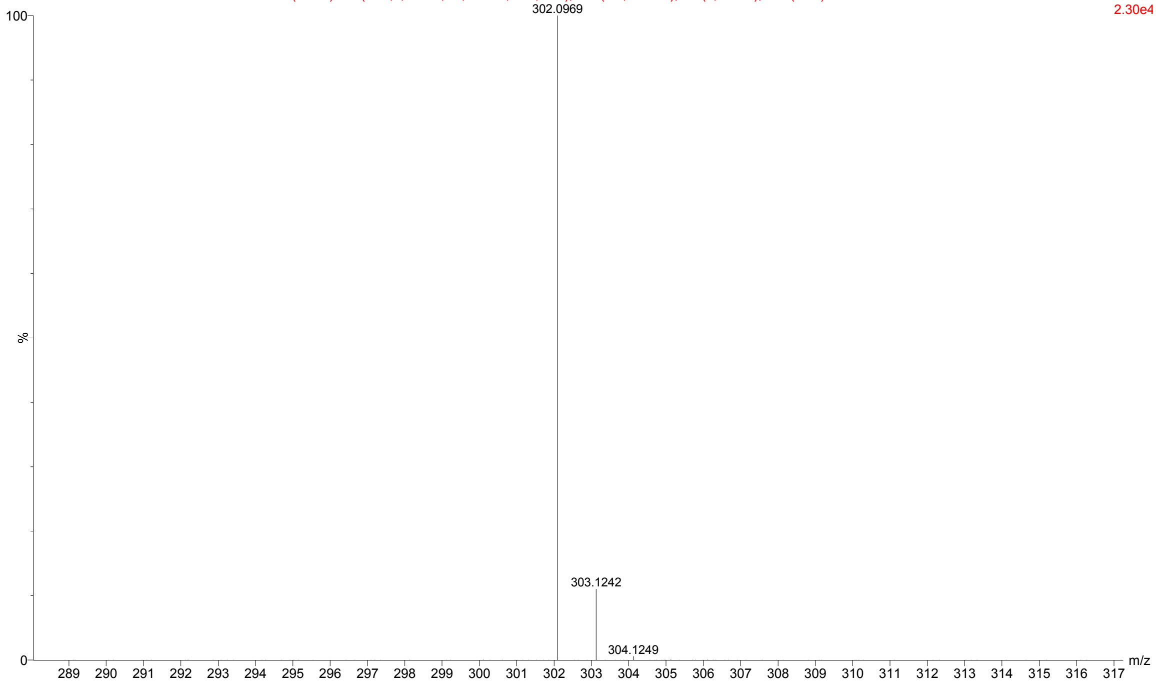

Supplement: Supplementary file 1 — Data S1. NMR and MS analyses of compounds used in the studies. [file prp20003-e00115-sd1.pdf]
